# Ionomic analysis, polyphenols characterization, analgesic, antiinflammatory and antioxidant capacities of *Cistus laurifolius* leaves: in vitro, in vivo, and in silico investigations

**DOI:** 10.1038/s41598-023-50031-5

**Published:** 2023-12-21

**Authors:** Aziz Zouhri, Toufik Bouddine, Naoual El Menyiy, Rabie Kachkoul, Yahya El-mernissi, Farhan Siddique, Rania moubachir, Farid Khallouki, Ahmad Mohammad Salamatullah, Gezahign Fentahun Wondmie, Mohammed Bourhia, Lhoussain Hajji

**Affiliations:** 1 Bioactives and Environmental Health Laboratory, Faculty of Sciences, Moulay Ismail University, B.P. 11201, Meknes, Morocco; 2 Laboratory of Pharmacology, National Agency for Medicinal and Aromatic Plants, 34025 Taounate, Morocco; 3 Laboratory of Biochemistry, Faculty of Medicine and Pharmacy, Sidi Mohammed Ben Abdellah University, Km 22, Road of Sidi Harazem, BP 1893, Fez, Morocco; 4 Research Unit in Applied Chemistry, Faculty of Science and Techniques, Abdelmalek Essaadi University, 32003 Al Hoceima, Morocco; 5 Laboratory of Organic Electronics, Department of Science and Technology, Linköping University, 60174 Norrköping, Sweden; 6 Ethnopharmacology and pharmacognosy Team, Department of Biology Moulay Ismail University of Meknes, BP. 52000 Errachidia, Morocco; 7 Department of Food Science & Nutrition, College of Food and Agricultural Sciences, King Saud University, 11, P.O. Box 2460, 11451 Riyadh, Saudi Arabia; 8 Department of Biology, Bahir Dar University, P.O.Box 79, Bahir Dar, Ethiopia; 9 Department of Chemistry and Biochemistry, Faculty of Medicine and Pharmacy, Ibn Zohr University, 70000 Laayoune, Morocco

**Keywords:** Drug discovery, Chemistry

## Abstract

This study aims to investigate the chemical and mineral composition, antioxidant, analgesic, and anti-inflammatory effects of the aqueous extract of *Cistus laurifolius* var. *atlanticus* Pit. (Cistaceae). Additionally, molecular docking interactions of various ligands with antioxidant protein target urate oxidase (1R4U) and anti-inflammatory protein target cyclooxygenase-2 (3LN1), revealing potential dual activities and highlighting specific residue interactions. The chemical characterization focused at first glance on the mineral composition which showed that *C. laurifolius* extract is a mineral-rich source of potassium (K), magnesium (Mg), manganese (Mn), sodium (Na), phosphorus (P), and zinc (Zn). We next performed, ultra-performance liquid chromatography-tandem mass spectrometry (UPLC-MS/MS) analysis, the latter showed various polyphenols in *C. laurifolius* extract including Gallic acid as the predominant polyphenol. Isoquercetin, Taxifolin and Astragalin were also among the major flavonoids detected. The antioxidant capacity of *C. laurifolius* leaves was tested using 2,2′-azino-bis (3-ethylbenzothiazoline-6-sulfonic acid) (ABTS), 2,2-diphenyl-1- picrylhydrazyl (DPPH) and reducing power (RP) assays. In vitro analysis of the anti-inflammatory property of *C. laurifolius* leaves was conducted by the albumin denaturation test and the in vivo was assessed in the sequel by carrageenan-induced paw edema test. The analgesic activity was evaluated in vivo using tail flick, acetic acid-induced contortion, and plantar tests. The findings showed that the leave extract had a powerful antioxidant activity with an IC_50_ values of 2.92 ± 0.03 µg/mL (DPPH) and 2.59 ± 0.09 µg/mL (in RP test). The studied extract strongly abolished the induced inflammation (82%). Albumin denaturation test recorded an IC_50_ value of 210 µg/mL. Importantly, the oral administration of *C. laurifolius* extract considerably reduced the nociceptive effect of acetic acid in rats, showing a significant analgesic effect in a dose-related manner. Altogether, our results showed that *C. laurifolius* can be a promising source of phytochemicals for drug development potential.

## Introduction

*Cistus laurifolius (C. laurifolius)* is a hermaphrodite shrub belonging to the family Cistaceae, known for its large white flowers. This plant is usually found in the under story and successive scrub of oak and pine forests, typically in Mediterranean mountains at elevations ranging from 800 to 1900 m^1^. The distribution of *C. laurifolius* is scattered, ranging from Morocco to the Black Sea region and Turkey, but with a disjunct distribution between the western and eastern Mediterranean basin^2,3^.

This plant is used in Moroccan traditional medicine as an Aphrodisiac and against cooling^4^, as well as to treat diabetes^5,6^. The plant extract has aroused the interest as promising anti-proliferative^7^, antimicrobial, antiviral^8^, and as antioxidant agent^9–12^. Furthermore, it could also maintain improvements in oxidative, and inflammatory pathways in diabetes^13,14^, prevent acetaminophen-induced liver damage^11^, inhibit Prostaglandins^12^ as well as inhibit skin aging^15^. Such myriad of biological activities are due especially to the presence of a number of phytochemicals in the plants including tannins, sterols, phenolic acids, lignan glycosides and flavonoids^11,12,14,16^.

Oxidative stress is an abnormal condition that can occur within cells or tissues^17^. It arises from the production of reactive oxygen species (ROS) or a decline in antioxidant defenses^18^. ROS can disrupt the cellular redox balance and cause damage to essential biomolecules such as proteins, lipids, and nucleic acids^19^. This can pose a risk to human health, as it has been linked to an increased prevalence of debilitating conditions such as cancer, diabetes, cardiovascular disease, aging, and neurological disorders^20,21^.

Inflammation is a defensive response that occurs in response to injury or infection from pathogens like bacteria or viruses. It is a complex process involving the identification of the affected tissue by cells, removal of the attacking agent, and tissue repair^22,23^. However, excessive inflammation can have negative effects and contribute to the development of chronic or prolonged diseases like atherosclerosis, rheumatoid arthritis, and systemic lupus erythematosus^22^. Innate immune cells, such as neutrophils and macrophages, generate reactive oxygen species (ROS) to eliminate pathogens. However, prolonged inflammation can lead to excessive ROS production, triggering oxidative stress. This oxidative stress can further promote inflammatory processes by activating transcription factors like NF-κB^22^.

As regards of Pain, it refers to an uncomfortable sensation that can range from mild discomfort to a distressing experience, and it is often associated with actual or potential tissue damage. Pain can be triggered by various external or internal noxious stimuli^24^. Sensory receptors are stimulated, transmitting pain signals to the brain for interpretation and response. Some nociceptors are activated only by intense stimulation, while others respond even to harmless stimuli as a warning^25^. However, chronic pain poses a significant challenge in the adult population, as effective drugs for its treatment are limited and may have adverse side effects^26^.

In recent times, there has been a growing interest in the search for natural anti-inflammatory and analgesic agents, as well as antioxidants for use in food and medicinal applications, to replace synthetic antioxidants that may have limitations and side effects such as carcinogenicity^27,28^. The rationale for choosing target proteins is rooted in their known roles in inflammation and oxidative stress, which align with the objectives of our research. Firstly, we selected the protein 3LN1 due to its established anti-inflammatory properties. Extensive studies have demonstrated that 3LN1 plays a crucial role in modulating the inflammatory response by inhibiting specific signaling pathways or interacting with key inflammatory molecules. By studying the interaction of our test compounds with 3LN1, we aim to evaluate their potential anti-inflammatory activity and gain insights into their mechanisms of action. Secondly, we included the protein 1R4U, known for its antioxidant properties. Oxidative stress is implicated in various pathological conditions, and identifying molecules that can effectively scavenge reactive oxygen species is of great interest. The selection of 1R4U enables us to assess the antioxidant capacities of our test compounds and explore their ability to counteract oxidative damage. In this study, our objective is to investigate the antioxidant, anti-inflammatory, and analgesic effects of the aqueous extract of *C. laurifolius* plant, and to identify and quantify its chemical compounds and mineral composition.

## Material and methods

### Plant material

In March 2022, leaves of the *Cistus laurifolius* plant were collected from the Ketama area (N: 34°48′49, W: 4°38′27, Altitude: 1189 m). The plant was identified by botanist Bari Amina and a voucher specimen with the number 0022023KC2 was deposited in the university herbarium. The leaves were carefully washed, dried at room temperature in the absence of light, and subsequently powdered finely.

### Preparation of plant extracts

Maceration method was used to prepare the plant extract. For this purpose, 100 g of raw material was macerated with 1 L of distilled water under stirring at 400 rpm for 10 h at room temperature. The solution was obtained by filtration on Whatman paper n°1. The obtained filtrate was frozen at – 30 °C, then lyophilized (FreeZone ® Dry 4.5. USA). The prepared aqueous extract (yield = 14.34%) was conserved at 4 °C until use.

### Standard compounds and chemical reagents

Reference compounds (all ≥ 98% purity) were purchased from Merck KGaA (Darmstadt, Germany) and Carl Roth GmbH (Darmstadt, Germany) (Table S1). All solvents were of analytical grade and were purchased from Sigma-Aldrich (Saint-Louis, USA). Chemicals, including 2,2′-diphenyl-1-picrylhydrazyl (DPPH), 2,2-azino-bis-3-ethylbenzothiazoline-6-sulfonic acid (ABTS), butylated hydroxytoluene (BHT), ascorbic acid, Folin-Ciocalteu reagent and carrageenan from Sigma-Aldrich (Saint-Louis, USA). The commercial drugs indomethacin, diclofenac sodium and acetylsalicylic acid were purchased from Pharma5 (Bouskoura, Morocco).

### Mineral elements content

The mineral composition of *C. laurifolius* leaves including Al, B, Ca, Cr, Cu, Fe, K, Mg, Mn, Na, Ni, P, Si, Sn, V and Zn, was determined using method ICP-AES (ICPE-9000 from Shimazu), as previously described in^29^.

### Flavonoid content (TFC), total phenolic content (TPC), and proanthocyanidin content (PC)

The Folin-Ciocalteu method, as previously described by Mǎrghitaş et al.^30^, was used for the quantification of phenolic content (TPC) in the *C. laurifolius* extract.

Gallic acid was used as the standard for the calibration curve and the results are expressed as mg gallic acid equivalent per g dry weight of extract (mg GAE/g extract).

The estimation of total flavonoid content (TFC) was conducted following the protocol outlined by Amezouar et al.^31^. Quercetin was used as a standard to construct the calibration curve. The results were expressed as mg equivalent of quercetin per g dry weight of extract (mg EQ/g extract).

The procedure outlined by Sayah et al.^32^ was used to determine the proanthocyanidin (PC) content. Catechin was used as a standard and results are expressed as catechin equivalent per gram dry weight of extract (mg EC/g extract).

### Identification and quantification of phenolic constituents by UPLC-ESI–MS/MS

The analysis of the aqueous extract of the plant was conducted using reverse-phase UPLC (Ultra-Performance Liquid Chromatography) on an Acquity UPLC system coupled with a Waters Xevo TQ-S triple quadrupole system (Waters, United States), following the methodology described in previous studies^33^. Detection was achieved via an electrospray ionization (ESI) interface in multiple reaction monitoring (MRM) and negative ionization mode, as indicated in Table S1.

The identification of phenolic compounds was confirmed by comparing the retention times and mass spectra of the peaks of our extract with those of the corresponding standards (chromatograms displaying the peak profiles of the target compounds are provided in the supplementary file).

### Antioxidant activity

The DPPH (DPPH•) radical scavenging activity of the tested extracts was evaluated according to previous work^33,34^. Briefly, 25 μL of extract at various dilutions were combined with 825 μL of DPPH reagent. The reaction mixture was thoroughly vortexed and incubated in the dark at room temperature for 30 min. The resulting coloration was measured at 517 nm using a spectrophotometer (UV-1700APC, China). The inhibition percentage was calculated using the following formula (1) and compared with the BHT standard.

1 $${\text{\% }}of\, inhibition = \frac{{\left( {control - samle} \right)}}{{\left( {control} \right)}} \times 100$$

The method outlined by Miguel et al.^35^ was used to determine the inhibitory effect of *C. laurifolius* extracts on the cation-based ABTS (ABTS^**•+**^) radical (2.2-azino-bis-3 ethylbenzothiazoline-6-sulfonic acid). An aqueous solution of ABTS (7 mM) was mixed with 2.5 mM potassium persulfate to generate ABTS^**•+**^. The mixture was allowed to sit at room temperature in the dark for 16 h. Subsequently, 25 μL of the extract at varying concentrations was combined with 825 μL of ABTS^**•+**^ reagent. After 6 min, the absorbance was measured at 734 nm. Ascorbic acid was used as the antioxidant standard. The % of inhibition was calculated using Eq. (2).

2 $${\text{\% }}of\, inhibition = \frac{{\left( {A0 - A1} \right)}}{A0} \times 100$$

(A0): absorbance at t0.

(A1): absorbance after 6 min.

The reducing potential (RP) of the extracts was determined following the procedure outlined by Bougandoura et al*.* (2013)^36^, with ascorbic acid used as a reference standard. The IC_50_ (µg/mL) values for the different assays were calculated based on the concentrations that resulted in 20–80% inhibition.

#### Total antioxidant capacity (TAC)

The phosphomolybdate (PPM) assay was conducted as follows: the phosphomolybdate reagent was prepared by mixing sulfuric acid (25 mL in 225 distilled water), sodium phosphate (3.28 g in 250 mL H_2_O) and ammonium molybdate (3.7 g in 250 ml H_2_O). Briefly, 25 μL of *C. laurifolius* extract and standard were mixed with 1 mL of phosphomolybdate reagent. Following an incubation period of 90 min at 96 °C, the absorbance was measured at 700 nm^31^. Total Antioxidant Capacity (TAC) was calculated using a calibration range established with ascorbic acid, and the results were expressed as milligrams of ascorbic acid equivalent per gram of raw plant (mg AAE/gE).

### Experimental animal protocol

This study utilized adult Wistar rats, which were housed in standard environmental conditions (22 ± 3 °C, 55 ± 5% humidity, and 12 h light/dark cycles) and had easy access to water. Prior to the experiment, both control (water) and experimental rats (given extracts or drugs) were fasted for 16 h. The animal house of NAMAP, Morocco, provided the rats, and all experimental procedures were conducted according to the guidelines established in the “Guide for the Care and Use of Laboratory Animals” by the National Academy of Sciences^37^.

### Anti-inflammatory properties

The anti-inflammatory potency was evaluated using the carrageenan-induced rat paw edema test, following the method described by Winter et al.^38^. Groups of 6 rats were formed for each treatment. *C. laurifolius* aqueous extract (500 mg/kg bw) was given orally 30 min before carrageenan injection. Indomethacin (10 mg/kg bw) and distilled water (5 mL/kg) were used as positive and negative controls respectively. Carrageenan (1%. w/v) prepared in (NaCl 0.9%) was used to induce edema paw. Paw volume was determined before carrageenan injection and after 1 h, 2 h, 3 h, 4 h, 5 h and 6 h of carrageenan injection using LE 7500 plethysmometer.

The anti-inflammatory potency was estimated as percent inhibition using the Eq. (3):

3 $$Inhibition\left( {\text{\% }} \right) = \frac{{\left( {Volume\; of\; the\; control - Volume\; of \;the \;treated \;groups} \right)}}{Volume\; of\; the\; control} \times 100$$

### Inhibition of albumin denaturation

To assess the in vitro anti-inflammatory efficacy of the aqueous extract of *C. laurifolius* leaves, the albumin denaturation assay according to^39^. In a nutshell, for the protein denaturation assay, 0.5 mL of bovine serum albumin (BSA) solution (0.2%) prepared in Tris buffer (pH 6.8) was mixed with 0.5 mL of various concentrations of *C. laurifolius* extracts or a standard (diclofenac sodium). The samples were initially incubated at 37 °C for 15 min and then immediately placed at 72 °C for 5 min. After cooling the tubes, the absorbance was measured at 660 nm, and the percentage of protein denaturation was calculated using the provided equation.

4 $$Inhibition\left( {\text{\% }} \right) = \frac{{Control - \left( {Sample - White} \right)}}{Control} \times 100$$

### Assessment of the analgesic effect

#### Writhing test

The acetic acid-induced contortion test was performed as previously reported by Sayah et al.^40^. Weight rats (180–200 g) were divided into three lots of 6 rats: lot 1 (control) was treated with 0.9% saline; lot 2 was previously treated with aspirin (150 mg/kg bw) and lot 3 received *C. laurifolius* extract (500 mg/kg bw). Contortions were induced in the study by intraperitoneal injections of a 3% acetic acid solution at a dose of 3.75 mL/kg bw, administered 30 min after the respective treatments.

Rats were arranged separately in specific cages and the contortions number were counted during an observation period of 10 min starting immediately after the injection of acetic acid. The inhibition (%) of abdominal constrictions was determined as follows (5):

5 $$Inhibition \left( {\text{\% }} \right) = \left( {1 - \frac{Contortion\; numbers \;the\; treated}{{Contortion\; numbers\; the\; control}}} \right) \times 100$$

#### Tail flick test

The tail flick test was performed in accordance with the method described by Sood et al.^41^. The response of the tail to radiant heat sensation was recorded using an ANALGESY-METER LE 7106, and a cut-off time of 20 s was maintained. The tail flick test was conducted for each group before treatment and at 30, 60, 90, and 120 min after drug administration.

#### Plantar test

The thermal plantar test was conducted using the UGO BASILE model 37370, following the method described by El Youbi et al.^42^. The paw withdrawal latency was measured automatically at 30, 60, 90, and 120 min after treatment.

#### Molecular docking

In this study, a molecular docking methodology was employed to identify potential ligands targeting anti-inflammatory and antioxidant proteins. The crystal structures of the proteins of interest, cyclooxygenase-2 (PDB ID: 3LN1)^43^ and urate oxidase (PDB ID: 1R4U)^44^, were obtained from the RCSB protein data bank. To prepare the protein structures for docking, various software tools were utilized. BIOVIA Discovery Studio Visualizer (Accelrys Software Inc., 2005) was employed to remove heteroatoms, co-crystal ligands, and solvent molecules, resulting in a clean protein structure. Auto-dock tools^45^ were then utilized to optimize the protein structure by assigning appropriate polar hydrogen and Kollman charges. The optimized protein structures were saved in the pdbqt file format. The ligands of interest were designed using Chemdraw Ultra^46^, energy minimized in Chem 3D Pro^47^, and converted into the pdbqt file format using the OpenBabel GUI program^48^. Autodock4, a widely used docking software, was employed for the molecular docking simulations^49,50^. Ligand drugs were docked independently into the active site of each protein target. To analyze and visualize the ligand–protein interactions, BIOVIA Discovery Studio Visualizer was used. The docking results were validated by calculating the root-mean-square deviation (RMSD) value and by re-docking the co-crystal ligand^51,52^. Acceptance of docking poses required RMSD values of less than 2.0 between the docked ligands and experimental ligands^53^.

### Statistical analysis

The results are presented as the mean ± standard error. GraphPad Prism 8.02 software was utilized for all statistical analyses. Comparison between groups was conducted using analysis of variance (ANOVA) followed by Tukey’s test for multiple comparisons. Statistical significance was considered at P ≤ 0.05.

### Ethics approval and consent to participate

Animal housing and experimental procedures were conducted in accordance with the European Union directive (2010/63/EU) and received approval from the Institutional Ethics Committee for the Care and Use of Laboratory Animals at the Faculty of Sciences, Meknes, University Moulay Ismail, Morocco, under the reference number 04/2019/LBEAS. No approval is needed to collect *Cistus laurifolius* in Morocco for research purposes. The collection of plant material complies with relevant institutional, national, and international guidelines and legislation. This study was in accordance with ARRIVE guidelines.

## Results and discussion

### Mineral composition

Quantified minerals in *C. laurifolius* leaves are presented in Table 1. The result obtained in this study showed that the leaves are a good source of minerals such as K, Ca, Mg and P. The concentrations are 661; 375; 77.5 and 44.8 mg/kg respectively. Among such minerals that fulfill physiological needs, we quote calcium which plays a crucial role as a structural element in bones and teeth, while also serving as a signaling molecule in vital physiological processes such as vascular contraction, blood clotting, muscle contraction, and nerve transmission. On the other side, magnesium plays a role in protein and nucleic acid synthesis and is critical for maintaining proper vascular tone and insulin sensitivity. Iron plays a variety of roles mainly oxygen transport, red blood cell formation as well as being a cofactor for enzymes. Zinc is a critical component of a variety of metalloenzymes involved in carbohydrate, lipid, protein and nucleic acid synthesis and degradation and in the metabolism of other micronutrients^54^.

**Table 1:** *Cistus laurifolius* mineral composition (mg/kg of powder).

Mineral	Content (mg/kg)	Mineral	Content (mg/kg)
K	661.0 ± 5.67	Mn	2.320 ± 0.230
Al	5.470 ± 0.27	Na	2.470 ± 0.130
B	0.732 ± 0.05	Ni	0.203 ± 0.090
Ca	375.0 ± 3.33	V	0.059 ± 0.003
P	44.80 ± 1.21	Zn	0.708 ± 0.050
Cr	0.204 ± 0.07	Si	12.00 ± 1.010
Cu	0.377 ± 0.12		
Fe	0.930 ± 0.05		
Sn	0.080 ± 0.01		
Mg	77.50 ± 1.98		

Values are expressed as mean ± SEM (n = 3).

### Contents of flavonoids, proanthocyanidins, and total phenolic compounds

TFC, PC, and TPC of the aqueous extract of *C. laurifolius* plant leaves are shown in the Table 2.

**Table 2:** TPC, TFC, and PC of *C.* *laurifolius* aqueous extract.

Extract	TPC (mg GAE/g of extract)	TFC (mg QE/g of extract)	PC (mg CE/g of extract)
aqueous extract	339.81 ± 0.65	28.94 ± 3.91	187.90 ± 3.93

Values are expressed as mean ± SEM (n = 3).

The quantitative analysis results (Table 2) demonstrate the high content of total polyphenols in the aqueous extract of *C. laurifolius*, with a concentration of 339.81 ± 0.65 mg GAE/g.

Concerning flavonoids, the value obtained was around 28.94 ± 3.91 mg EQ/g. Moreover, proanthocyanidins, which are condensed tannins with diverse pharmacological properties, were found at a high level with a value of 187.90 ± 3.93 mg EQ/g. The comparison of these outcomes found in this study with the literature, especially those working on Turkey samples, shows that the contents of this extract exceed those found by Orhan et al.^14^, who reported polyphenol concentrations of 323.9 ± 7.1 and 289.9 ± 12.9 g GAE/g in the ethanolic and aqueous extracts, as well as that of İnan et al.^55^ for proanthocyanidins. While, the flavonoid content found in the work of Akkol et al.^10^ was higher compared to ours with values of 270.80 and 255.85 mg/g in n-BuOH and EtOAc extracts respectively.

### Phenolic compound quantification from aqueous extract of *C. laurifolius*

We employed the MRM (Multiple Reaction Monitoring) technique to quantify the phenolic compounds of our extract, a series of dilutions of the phenolic stock solutions were prepared to perform the calibration range with a linearity correlation coefficient at R^2^ > 0.991. The results were expressed in mg/kg of plant extract and represented in Table 3.

**Table 3:** UPLC-ESI–MS/MS quantification of the phenolic compositions in the aqueous extract of *Cistus laurifolius.*

Compound number	Compounds	Chemical class	MS/MS fragments	Retention time (min)	Content (mg/kg)
I	Gallic acid	Phenolic acids	125 79	4.33	4597.701 ± 18.02
II	Protocatechuic acid	Phenolic acids	109	6.25	255.058 ± 9.71
III	Isoquercetin	Flavonoids	300 271	13.18	249.958 ± 9.81
IV	4-OH-phenylacetic acid	Phenylacetic acids	107	7.3	197.104 ± 2.64
V	P- Coumaric acid	Hydroxycinnamic acid derivatives	119	11.5	171.579 ± 7.97
VI	Vanillic acid	Phenolic acids	152	8.92	72.975 ± 1.062
VII	Avicularin	Flavonoids	300 271	13.91	70.39 ± 0.219
VIII	Quinic acid	Quinates precursors	85 93	2.16	44.997 ± 0.608
IX	Taxifolin	Flavonoids	285 125	12.45	35.624 ± 1.674
X	Astragalin	Flavonoids	284 255	14.03	33.842 ± 2.136
XI	Aromadendrin	Flavonoids	259 125	14.15	20.827 ± 0.300
XII	Ferulic acid	Hydroxycinnamic acid derivatives	134 178	11.94	19.24 ± 0.121
XIII	Rutin	Flavonoids	300 271	12.78	18.895 ± 0.454
XIV	Quercetin	Flavonoids	151 179	16.53	18.751 ± 1.134
XV	Phloridzin	Flavonoids	273 167	13.77	17.589 ± 0.270
XVI	Gentisic acid	Phenolic acids	109	8.09	14.741 ± 0.552
XVII	Epicatechin	Flavonoids	245 109	9.80	14.038 ± 0.208
XVIII	Catechin	Flavonoids	245 109	8.82	8.891 ± 0.443
XIX	Procyanidin B2	Flavonoids	407 289	9.05	6.033 ± 0.545
XX	Quercetrin	Flavonoids	300 271	14.23	5.891 ± 0.441
XXI	Hydroferulic acid	Hydroxycinnamic acid derivatives	121 93	10.09	5.213 ± 0.074
XXII	Kaempferol	Flavonoids	93 146	17.99	4.742 ± 0.140
XXIII	Caffeic acid	Hydroxycinnamic acid derivatives	135 107	9.58	3.804 ± 0.024
XXIV	Naringenin	Flavonoids	151 119	16.74	3.576 ± 0.175
XXV	Salicylic acid	Phenolic acids	93	12.43	2.433 ± 0.105
XXVI	Apigenin	Flavonoids	117 149	17.74	2.005 ± 0.068
XXVII	Luteolin	Flavonoids	133 107	16.48	1.351 ± 0.141
XXVIII	3,4,5 trimethoxycinnamic acid	Phenylpropanoids	102 132	14.71	0.943 ± 0.050
XXIX	Isorhamnetin	Flavonoids	300 151	17.92	0.837 ± 0.133
XXX	Quercetin-3-*O*-glucuronide	Flavonoids	301 151	13.49	0.798 ± 0.152
XXXI	Sinapinic acid	Hydroxycinnamic acid derivatives	208 164	14.71	0.56 ± 0.127
XXXII	Resveratrol	Stilbenes	142 158	15.49	0.374 ± 0.028
XXXIII	Apigetrin	Flavonoids	268 107	14	0.271 ± 0.070
XXXIV	Phloretin	Dihydrochalcone	167 123	16.85	0.134 ± 0.019

Values are expressed as mean ± SEM (n = 3).

The plant extract is very rich in phenolic acid, especially gallic acid, Protocatechuic acid, p- Coumaric acid and Vanillic acid with an amount of 4597.701 ± 18.028; 255.058 ± 9.713; 171.579 ± 7.972 and 72.975 ± 1.062 mg/kg respectively. The flavonoids diversity showed a significant content of Quercetin and its 3-*O*-glucopyranoside form (Isoquercetin) with average amounts of 18.751 ± 1.134 mg/kg and 249.958 ± 9.81 mg/kg respectively. Other main flavonol subclass include Avicularin, Astragalin and Rutin are the most quantified species of the flavonol subclass, and their content were amounted to 70.39 ± 0.219; 33.842 ± 2.136 and 18.895 ± 0.454 mg/kg respectively. Furthermore, other flavanol including Kaempferol, Isorhamnetin and Quercetin-3-*O*-glucuronide were found with moderate levels (4.742 ± 0.140; 0.837 ± 0.133 and 0.798 ± 0.1524 mg/kg respectively). Two flavanonols were quantitated with medium amounts, namely Taxifolin (35.624 ± 1.674 mg/kg) and Aromadendrin (20.827 ± 0.300 mg/kg). The flavanol subclass was represented by two species in moderate quantities, these were respectively Epicatechin (14.038 ± 0.208 mg/kg) and its epimer Catechin (8.891 ± 0.443 mg/kg). In sum, gallic acid and p-coumaric acid contents achieved in the current study are consistent with what was previously reported^13^. Compared to this last study, essentially the same metabolites were found although quantified with higher levels than ours. However Vanillic acid and Catechin which were revealed with a remarkable amount our study, were not detected previously in this species^13^.

### Antioxidant activity

The antioxidant capacity of the extract was assessed in vitro using ABTS, DPPH, and RP assays, while the TAC was determined using the phosphomolybdate test (PPM). Ascorbic acid and BHT were used as positive controls due to their well-known antioxidant properties. The median inhibitory concentrations (IC_50_) are listed in Table 4 to assess the antiradical efficacy of the plant extract.

**Table 4:** (DPPH, ABTS, RP and molybdate) of *C. laurifolius* aqueous extracts and controls.

	IC_50_ (μg/ml)			Molybdate (mg AAE/gE)
	DPPH	ABTS	RP	
Aqueous extract	2.92 ± 0.03****	2.48 ± 0.11***	2.59 ± 0.09	94.62 ± 1.69
BHT	4.43 ± 0.075	–	–	–
Ascorbic acid	–	2.32 ± 0.03	2.55 ± 0.08	–

^***^P < 0.001; ^****^P < 0.0001 vs, control.

According to the result of the DPPH method (Table 4), the plant extract shows a high capacity to reduce free radicals and is significantly better than the positive control (BHT) known by its antioxidant effect, this is most likely due to the presence of gallic acid in appreciable amount in the water extract. Indeed, this capacity is demonstrated by a lower IC_50_ of about 2.92 ± 0.03 μg/mL (P < 0.05 vs. BHT), compared to 4.43 ± 0.075 μg/mL for BHT. Besides, both ABTS and RP methods also exhibit a very high antiradical effect comparable to those of Ascorbic acid with values of 2.48 ± 0.11 μg/mL (P < 0.05 vs. Ascorbic acid) and 2.59 ± 0.09 μg/mL (P < 0.05 vs. Ascorbic acid) respectively. In addition, a strong total antioxidant property was revealed by the molybdate test with a value of 94.62 ± 1.69 mg EAA/g extract. The results indicate that the plant extracts are very effective in scavenging free radicals, by converting them into more stable products. In the DPPH test, the mechanism involves hydrogen donation to free radicals, leading to their reduction to nonreactive species.

The plant extract also exhibits activity in reducing the Fe3+/ferricyanide complex to Fe2+ form^56,57^. Furthermore, the observed antiradical efficacy of these extracts is likely attributed to the presence of polyphenols, such as phenolic acids and flavonoids, which are quantified in the extract. Yet, gallic acid has a DPPH radical scavenging activity of 92.57 ± 0.10 at 1000 µg/mL, while the Kaempferol derivative namely 3,7-*O*-dimethylkaempferol showed a value of 85.09 ± 0.16% at 2000 µg/mL^10^.

### Anti-inflammatory effect

**Figure 1:**
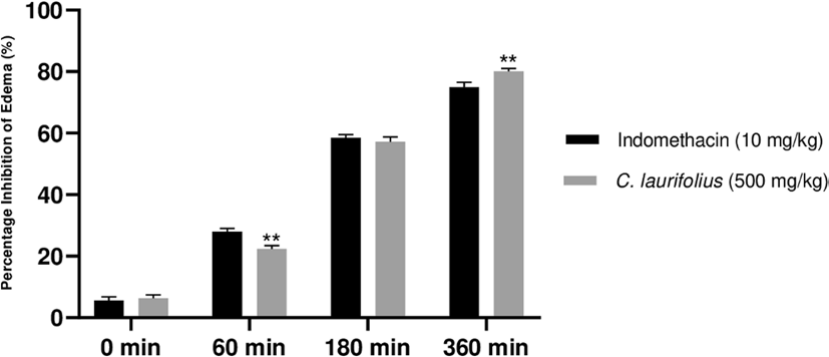
Anti-inflammatory efficacy of aqueous extracts of *C. laurifolius*. **P < 0.001.

**Figure 2:**
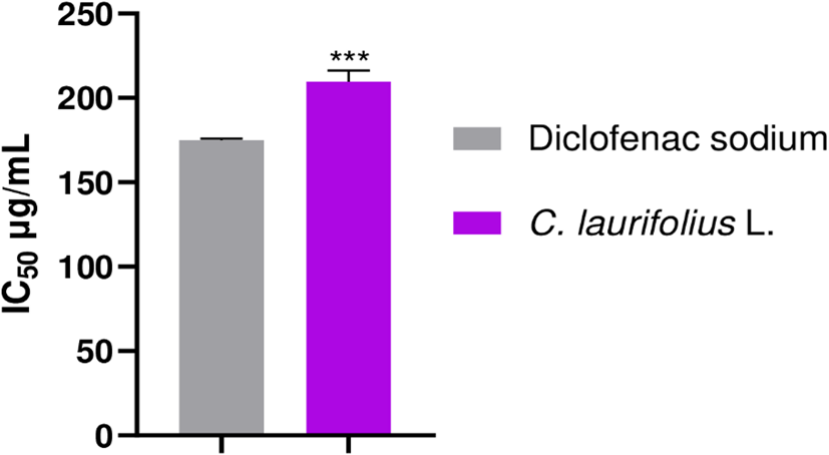
IC_50_ values of inhibition of albumin denaturation of aqueous extract of *C. laurifolius* and diclofenac sodium. The results are considered significantly different for ^***^P < 0.001.

The anti-inflammatory efficacy of aqueous extract of *C. laurifolius* was tested both in vivo, using the carrageenan-induced rat paw edema test, and in vitro, using the albumin denaturation test. The findings are depicted in Figs. 1 and 2.

According to Fig. 1 corresponding to the carrageenan-induced rat paw oedema test, the plant extract with a dose of 500 mg/kg shows a significant effect (P < 0.05) when compared to the control, indomethacin: a drug usually used against inflammation. The percentage inhibition of extract after 60 min is less effective compared to the control with a value of 25% (P < 0.05) against 30% for the control, but after 360 min it goes to a value of 82% (P < 0.05), while the control reaches just 77%. Regarding the albumin denaturation test (Fig. 2), the efficacy of the extract is somewhat close to that of the drug Diclofenac sodium (a non-steroidal anti-inflammatory drug), which has an IC_50_ value of 180 µg/mL, whereas that of the extract is 210 µg/mL.

Additionally, our results after 180 and 360 min of treatment with carrageenan were superior to those reported by Küpeli et al.^58^, who worked on Aqueous extract, EtOH extracts and the fraction of hydroethanolic extract. Moreover, the anti-inflammatory effect of *C. laurifolius* has been verified by other methods in the literature, the acetic acid-induced increased vascular permeability model showing a significant effect of the CHCl_3_ and EtOAc fractions according to the study of^58^. The methanol plant leaves extract, as well as various fractions, inhibit inflammatory cytokines such as TNF-α, IL-1 and IL-1β^59^. Yet, this activity is probably due to the presence of phenolic compounds and their derivatives, furthermore, Toker et al.^60^ found a potent anti-inflammatory activity of kaempferol-3,7-*O*-α-dirhamnoside and quercetin-3,7-*O*-α-dirhamnoside. Indeed, quercetin and its derivative as well as kaempferol, showed anti-inflammatory activity via inhibition of COX2 and iNOS^61,62^.

### Analgesic effects

**Figure 3:**
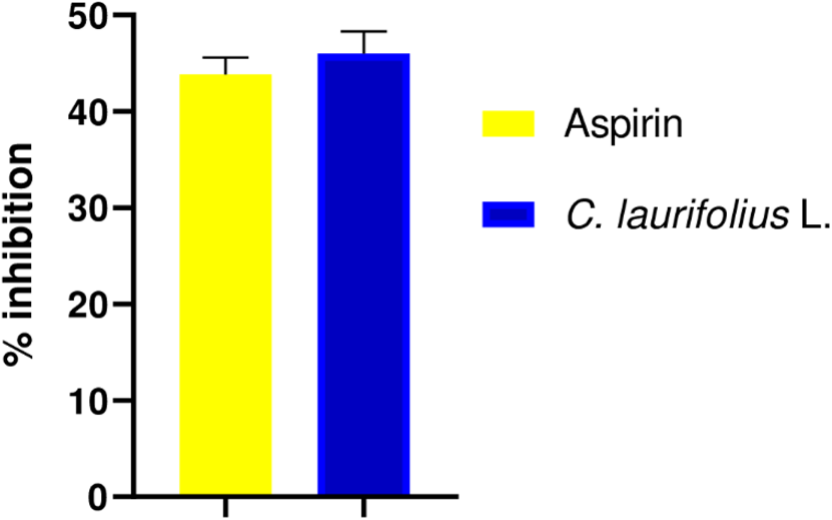
Effect of *C. laurifolius* L. aqueous extract on acetic acid-induced writhing in rats.

**Figure 4:**
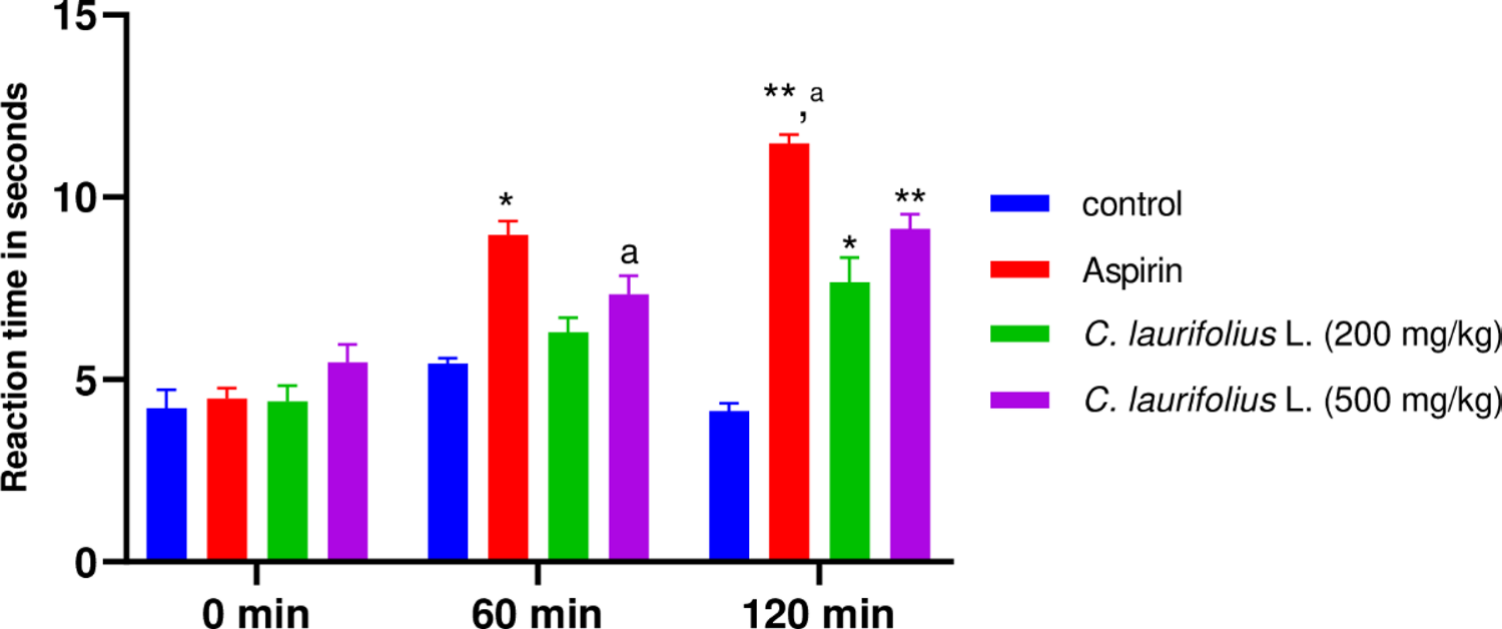
Latency of the tail-flick of aqueous extract of *C. laurifolius*. Statistically significant difference: ^*^P < 0.05; ^**^P < 0.01 vs. control. ^*a*^*P* < 0.05 vs. *C. laurifolius* L. (200 mg/kg).

**Figure 5:**
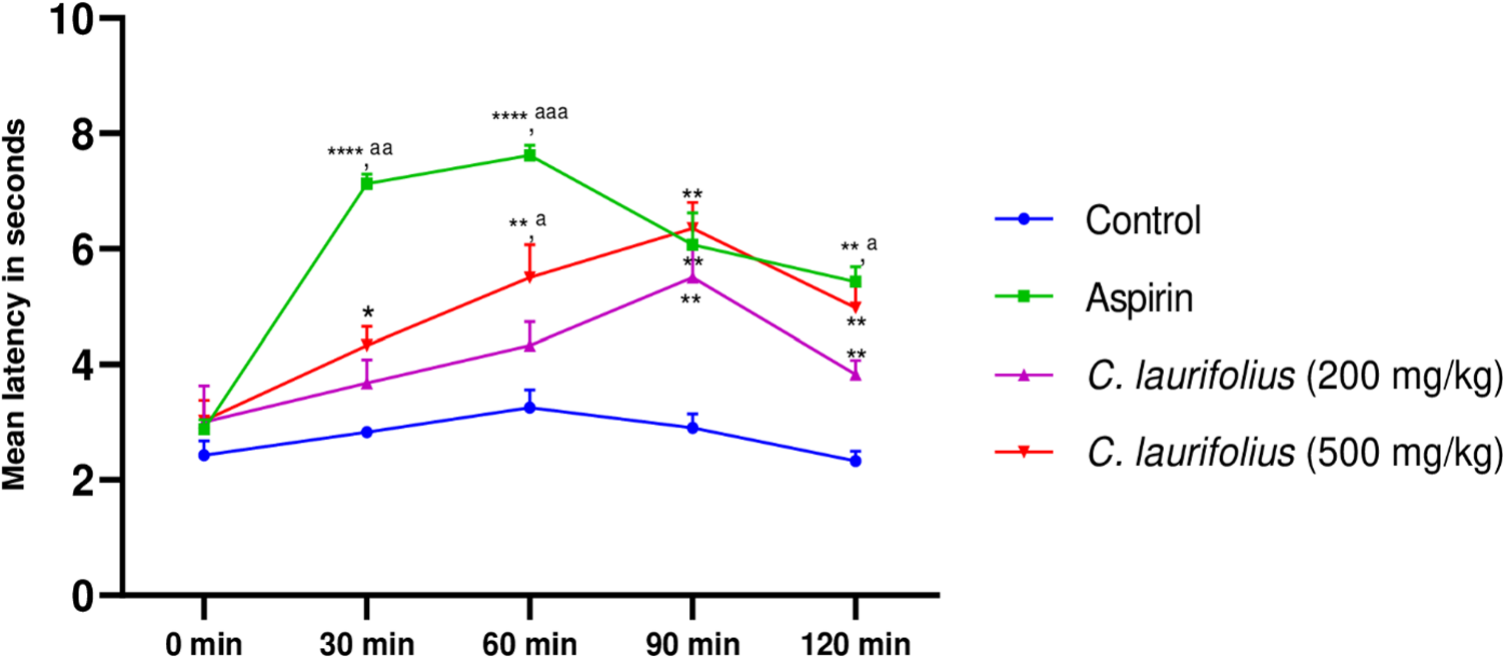
Effect of *C. laurifolius* aqueous extract in the plantar test. Statistically significant difference: ^*^P < 0.05; ^**^P < 0.01; ^***^P < 0.001 vs. control. ^*a*^*P* < 0.05; ^*aa*^*P* < 0.01; ^*aaa*^*P* < 0.001 vs. *Cistus laurifolius* (200 mg/kg).

The analgesic effect of plant extract was studied by three methods namely Writhing, Tail flick, and Plantar tests, the finding are shown in Figs. 3, 4, and 5 respectively.

Oral administration of the aqueous extract of the plant *C. laurifolius* at a dose of 500 mg/kg (Fig. 3), considerably reduced the nociceptive effect of acetic acid in rats compared to aspirin: a well-known peripheral analgesic drug, used as a positive control in the current research. Furthermore, the writhing inhibition percentage of the extract was 48% compared to 44% for aspirin.

According to the outcome of the Tail-flick test represented in Fig. 4, the plant extract shows a significant analgesic effect in a dose-related manner. This activity is demonstrated by the increase in the reaction time of the rats to radiant heat, in fact, after 60 min of drug administration, the time reaction was 5.5 and 7 s for the extract doses of 200 and 500 mg/kg, while aspirin has a slightly longer time (9 s). Nevertheless, reaction time is high after 120 min of drug administration and reaches the values of 7 and 9 s (P < 0.05) for both doses respectively, these values remain slightly lower than those of aspirin (12 s).

On the other hand, the plantar test also revealed a significant effect of the plant extract and is also dose-dependent, the maximum effect of the extract for both doses (200 and 500) are observed after 1.5 h of extract administration, whereas, aspirin takes only one hour to reach the maximum effect, pain tolerance time is estimated at 5 and 6 s for the extract doses of 200 and 500 mg/kg respectively, compared to 7.7 s for aspirin.

The plant extract examined in this study demonstrated significant peripheral and central analgesic effects. Remarkably, the peripheral analgesic effect of the extract surpassed that of aspirin, as evidenced by the acetic acid-induced writhing test. Acetic acid is known to induce nociception, leading to pain characterized by abdominal muscle contraction, forelimb expansion, and body elongation^63^. This is thought to be mediated through the activation of local peritoneal receptors and prostaglandin pathways^64^. Moreover, acetic acid stimulates the release of endogenous pain mediators, including histamine, serotonin, and bradykinin, which are implicated in the cardinal signs of inflammation^65^. Furthermore, our plant extract demonstrated a significant inhibition of acetic acid-induced torsion, indicating a potential blockage or inhibition of the prostaglandin pathway in the pain perception cascade, or possibly through the inhibition of the cyclooxygenase enzymatic pathway^25,66^. In addition, the plant extract has a central analgesic effect which is reflected in the prolongation of the thermal reaction time, which explains the increased pain tolerance in the rats. The observed analgesic activity of the extracts may be attributed to their phenolic compound content, including phenolic acids and flavonoids. These compounds have been shown to inhibit the production of arachidonic acid, prostaglandins, and leukotrienes, as well as reduce intracellular Ca^2+^ levels. Moreover, they can interact with 5-HT2A and 5-HT3 receptors, which are believed to be involved in the mechanism of analgesic activity^67,68^. In addition, they are responsible for the free radical scavenging activity which may be involved in pain stimulation^67^.

Our results demonstrate that the extract possesses strong antioxidant activity related to various phenolic compounds and exhibits significant anti-inflammatory and analgesic effects in vitro and in vivo. Furthermore, the use of multiple assays and models to evaluate these effects enhances the reliability and robustness of our findings.

However, there are also some limitations to our study. Indeed, we only used the aqueous extraction method, and it is possible that other extraction methods could result in different chemical profiles and bioactivities. Additionally, while we used a variety of tests and models to evaluate the bioactivities of the extract, further studies are needed to fully elucidate the mechanisms underlying these effects.

### Molecular docking

**Figure 6:**
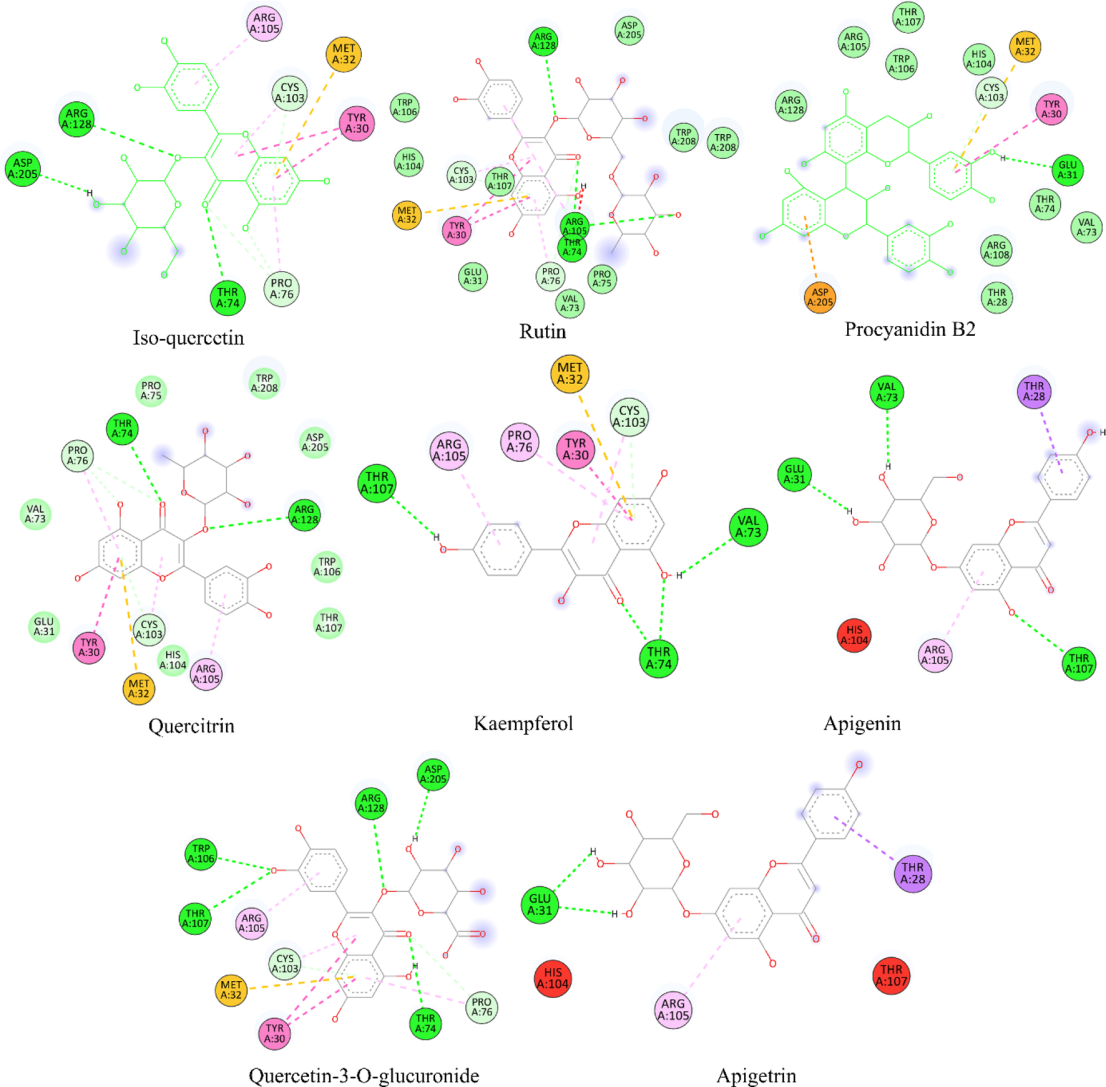
2D interactive view for anti-oxidative protein 1R4U by molecular docking.

The molecular docking results for the investigated ligands with protein target 1R4U are presented in Table 5. These results provide valuable insights into the binding interactions between the ligands and the target protein, along with their corresponding binding scores and specific residues involved in the interactions. Isoquercetin and Rutin, both exhibiting a binding score of − 8.8 kcal/mol, showed interactions with several residues in the binding pocket. For Isoquercetin, the key interaction residues included THR74, ARG128, ASP205, PRO76, CYS103, MET32, TYR30, and ARG105. Similarly, Rutin demonstrated interactions with THR74, ARG105, ARG128, PRO76, CYS103, MET32, TYR30, and ARG105. These interactions occurred at specific distances, ranging from 2.33 to 5.63 Å. Procyanidin B2, with a slightly higher binding score of − 9.4 kcal/mol, interacted with GLU31, ASP205, CYS103, MET32, TYR30, and PRO76 at varying distances, suggesting strong binding interactions. Quercetrin exhibited a binding score of − 9.3 kcal/mol and interacted with THR74, ARG128, PRO76, CYS103, MET32, TYR30, and ARG105. Multiple interactions were observed with TYR30 and PRO76 residues, indicating their significant role in binding Quercetrin. Kaempferol, with a binding score of − 8.5 kcal/mol, interacted with THR74, THR107, VAL73, CYS103, MET32, TYR30, CYS103, PRO76, and ARG105. Notably, THR74 and THR107 residues were involved in multiple interactions, suggesting their importance in the binding process^69^. Apigenin, having a binding score of − 8.6 kcal/mol, formed interactions with THR107, VAL73, GLU31, THR28, and ARG105 residues, highlighting their contribution to the binding affinity^70^. Lastly, Quercetin-3-*O*-glucuronide exhibited a binding score of -9.0 kcal/mol and displayed interactions with THR74, TRP106, THR107, ARG128, ASP205, PRO76, CYS103, MET32, TYR30, and ARG105 residues. Similar to Isoquercetin and Rutin, TYR30 and PRO76 residues played a crucial role in the binding of Quercetin-3-*O*-glucuronide. Gallic acid interacts with GLU31, THR74, HIS104, CYS103, MET32, TYR30, and PRO76 residues of the target protein. These interactions involve a combination of hydrogen bonding and hydrophobic interactions. Hydrogen bonding: GLU31, THR74, HIS104, and TYR30 may form hydrogen bonds with Gallic acid, where the hydroxyl groups of Gallic acid interact with the amino acid side chains. Hydrophobic interactions: CYS103, MET32, and PRO76 are likely to engage in hydrophobic interactions with Gallic acid, where the nonpolar parts of Gallic acid interact with nonpolar amino acid side chains. Protocatechuic acid interacts with GLU31, PRO76, CYS103, MET32, and TYR30 residues of the target protein. These interactions also involve a combination of hydrogen bonding and hydrophobic interactions. Hydrogen bonding: GLU31 and TYR30 may form hydrogen bonds with Protocatechuic acid, like the interactions observed with Gallic acid. Hydrophobic interactions: PRO76, CYS103, and MET32 likely engage in hydrophobic interactions with Protocatechuic acid, similar to the interactions observed with Gallic acid. All the interactions as 2D interactive view are also shown in Fig. 6 and detailed list of docking results are presented in supplementary material Table S2 for 1R4U.

**Table 5:** Molecular Docking score, hydrogen binding, hydrophobic and electrostatic interactions with distances in Angstrom for investigated ligands using protein target 1R4U.

Ligand code	Chemical name	1R4U antioxidant protein		
		Binding score Kcal/mol	Binding pocket interaction residues and distance Å	Type of interactions
3	Isoquercetin	− 8.8	THR74(2.40) ARG128(2.97) ASP205(2.21) PRO76(3.79) PRO76(3.16) CYS103(3.67) MET32(5.26) TYR30 (5.58) TYR30 (4.99) CYS103(4.33) PRO76(5.46) ARG105(4.28)	H-Bond H-Bond H-Bond H-Bond H-Bond Hydrophobic Hydrophobic Hydrophobic Hydrophobic Alkyl Alkyl Pi-alkyl
13	Rutin	− 8.8	THR74(2.33) ARG105(2.78) ARG128(2.94) PRO76(3.52) CYS103(3.61) MET32(5.26) TYR30(5.63) TYR30 (5.04) CYS103(4.30) PRO76(5.39) ARG105(4.47)	H-Bond H-Bond H-Bond H-Bond H-Bond Alkyl Alkyl Alkyl Hydrophobic Hydrophobic Hydrophobic
19	Procyanidin B2	− 9.4	GLU31(2.21) ASP205(4.12) CYS103(3.87) MET32(5.85) TYR30 (4.92) PRO76(5.46)	H-Bond Hydrophobic H-Bond Alkyl Pi-alkyl Pi-alkyl
20	Quercetrin	− 9.3	THR74(2.49) ARG128(2.87) PRO76(3.67) PRO76(3.35) CYS103(3.59) MET32(5.29) TYR30 (5.07) CYS103(4.14) PRO76(5.41) ARG105(4.18)	H-Bond H-Bond H-Bond H-Bond H-Bond Hydrophobic Hydrophobic Alkyl Van der waals Hydrophobic
22	Kaempferol	− 8.5	THR74(2.44) THR74(2.54) THR107(2.31) VAL73(2.45) CYS103(3.69) MET32(5.41) TYR30 (4.99) CYS103(4.43) PRO76(5.48) ARG105(4.19)	H-Bond H-Bond H-Bond H-Bond Hydrophobic Alkyl Hydrophobic Hydrophobic Alkyl Hydrophobic
26	Apigenin	− 8.6	THR107(2.94) VAL73(2.56) GLU31(2.28) THR28(3.87) ARG105(4.82)	H-Bond H-Bond H-Bond Hydrophobic Hydrophobic
30	Quercetin-3-*O*-glucuronide	− 9.0	THR74(2.47) TRP106(2.67) THR107(2.86) ARG128(2.86) ASP205(2.36) PRO76(3.63) CYS103(3.60) MET32(5.28) TYR30 (5.80) TYR30 (5.06) CYS103(4.15) PRO76(5.39) ARG105(4.20)	H-Bond H-Bond H-Bond H-Bond H-Bond Hydrophobic Hydrophobic Van der waals Van der waals Van der waals Alkyl Van der waals Alkyl
33	Apigetrin	− 8.7	GLU31(2.02) GLU31(2.17) THR28(3.85) ARG105(4.81)	H-Bond H-Bond Alkyl Alkyl

**Figure 7:**
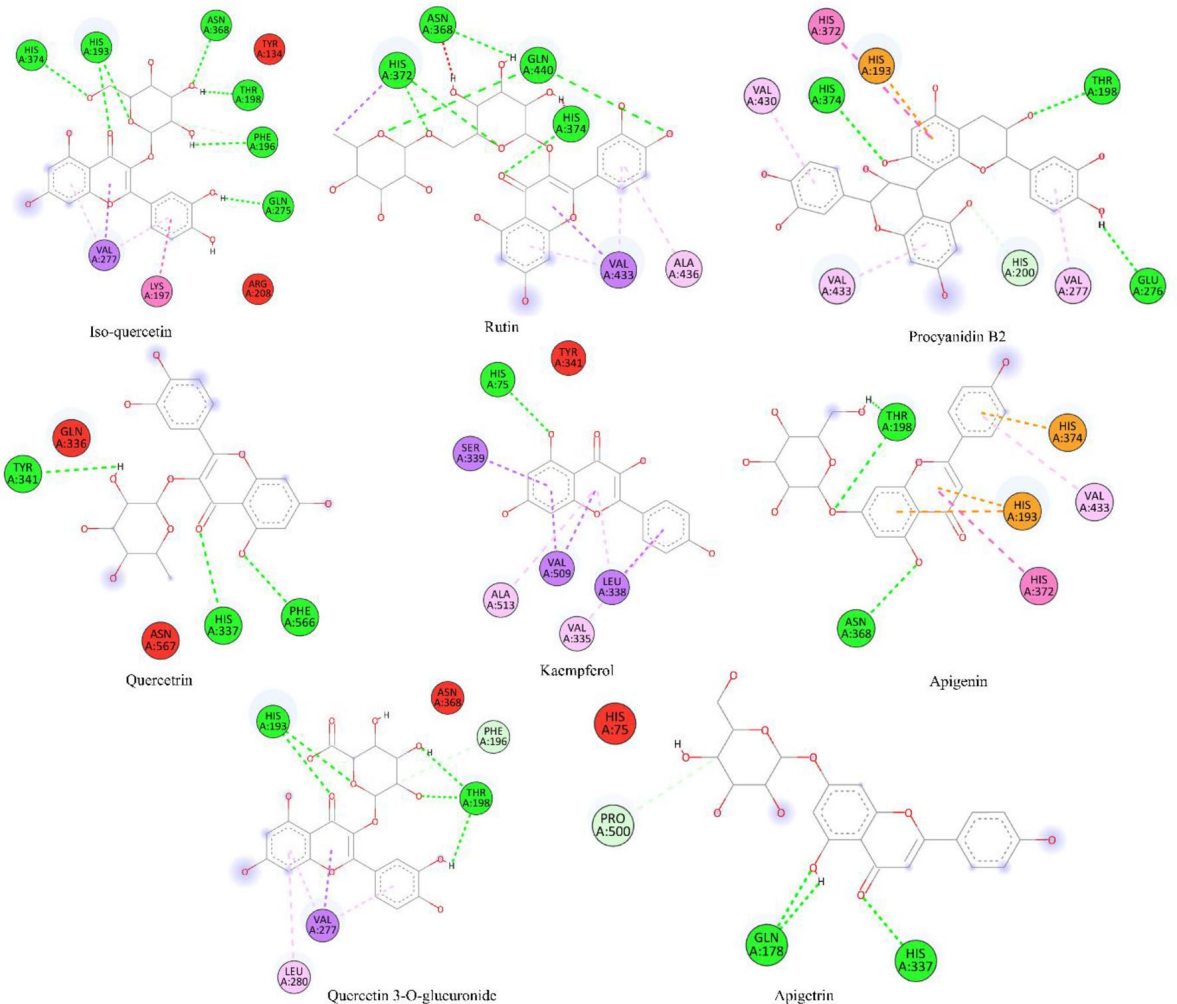
2D interactive view for anti-inflammatory protein 3LN1 by molecular docking.

Table 6 presents the molecular docking results for the investigated ligands with protein target 3LN1, which is associated with anti-inflammatory properties. These results provide information about the binding scores and specific interactions between the ligands and the target protein. Isoquercetin, with a binding score of − 8.1 kcal/mol, formed interactions with multiple residues, including HIS193, ASN368, HIS374, GLN275, PHE196, THR198, VAL277, and LYS197. Notably, THR198 and VAL277 were involved in multiple interactions, suggesting their importance in the binding process. Rutin demonstrated a higher binding score of − 9.2 kcal/mol and exhibited interactions with HIS372, HIS374, GLN440, ASN368, VAL433, and ALA436 residues. Similar to Isoquercetin, VAL433 played a significant role in the binding interactions. Procyanidin B2, with a binding score of − 8.9 kcal/mol, interacted with THR198, HIS374, GLU276, HIS200, HIS193, and HIS372 residues. These interactions suggest potential contributions to the ligand–protein binding affinity. Quercetrin, with a binding score of − 8.2 kcal/mol, formed interactions with HIS337, PHE566, and TYR341 residues. Although the interactions were fewer compared to other ligands, they still play a role in the overall binding process. Kaempferol displayed a binding score of − 8.7 kcal/mol and exhibited interactions with HIS75, LEU338, SER339, VAL509, and ALA513 residues. Notably, LEU338 and VAL509 were involved in multiple interactions, suggesting their significance in ligand binding. Apigenin, with a binding score of -8.4 kcal/mol, formed interactions with THR198, ASN368, HIS193, HIS374, and HIS372 residues. These interactions, particularly with HIS372, contribute to the binding affinity of Apigenin. Quercetin-3-*O*-glucuronide exhibited a binding score of − 8.5 kcal/mol and demonstrated interactions with HIS193, THR198, PHE196, VAL277, and LEU280 residues. The involvement of multiple residues suggests a complex binding pattern for this ligand. Finally, Apigetrin displayed a binding score of − 8.5 kcal/mol and formed interactions with GLN178, HIS337, and PRO500 residues. All the interactions as 2D interactive view are also shown in Fig. 7 and detailed list of docking results are presented in supplementary material Table S3 for 3LN1.

**Table 6:** Molecular docking score, hydrogen binding, hydrophobic and electrostatic interactions with distances in Angstrom for investigated ligands using protein target 3LN1.

Ligand code	Chemical name	3LN1 anti-inflammatory Protein target		
		Binding score	Interactions	Type of interactions
3	Isoquercetin	− 8.1	HIS193(2.78) HIS193(2.18) ASN368(2.27) HIS374(2.70) GLN275(2.30) PHE196(2.73) THR198(2.99) PHE196(2.18) VAL277(3.25) LYS197(3.50) THR198(5.20) VAL277(5.33) VAL277(4.66)	H-Bond H-Bond H-Bond H-Bond H-Bond H-Bond H-Bond H-Bond Hydrophobic Alkyl Alkyl Pi-alkyl Pi-alkyl
13	Rutin	− 9.2	HIS372(2.72) HIS372(2.23) HIS374(1.79) GLN440(2.47) GLN440(2.32) ASN368(2.46) VAL433(3.96) VAL433(3.77) HIS372(3.80) VAL433(5.20) VAL433(4.84) ALA436(5.07)	H-Bond H-Bond H-Bond H-Bond H-Bond H-Bond Hydrophobic Hydrophobic Hydrophobic Hydrophobic Hydrophobic Alkyl
19	Procyanidin B2	− 8.9	THR198(2.61) HIS374(2.37) GLU276(2.52) HIS200(3.60) HIS193(3.82) HIS193 (4.73) HIS372 (5.38) VAL433(4.95) VAL430(5.17) VAL277(3.86)	H-Bond H-Bond H-Bond Alkyl Pi-alkyl Pi-alkyl Pi-alkyl Hydrophobic Pi-alkyl Hydrophobic
20	Quercetrin	− 8.2	HIS337(2.41) PHE566(2.28) TYR341(2.26)	H-Bond H-Bond H-Bond
22	Kaempferol	− 8.7	HIS75(2.17) LEU338(3.70) SER339(3.59) VAL509(3.85) VAL509(3.32) LEU338(4.94) ALA513(5.35) VAL335(4.95)	H-Bond Hydrophobic Hydrophobic Hydrophobic Hydrophobic Alkyl Hydrophobic Hydrophobic
26	Apigenin	− 8.4	THR198(2.30) ASN368(3.05) THR198(2.21) HIS193(4.36) HIS193(3.51) HIS374(3.26) HIS372 (5.65) VAL433(5.10)	H-Bond H-Bond H-Bond Hydrophobic Hydrophobic Hydrophobic Alkyl Alkyl
30	Quercetin-3-*O*-glucuronide	− 8.5	HIS193(2.83) HIS193(2.48) THR198(2.36) THR198(1.99) THR198(2.01) PHE196(3.34) VAL277(3.54) VAL277(5.29) LEU280(5.38) VAL277(4.65)	H-Bond H-Bond H-Bond H-Bond H-Bond Hydrophobic Hydrophobic Van der waals Van der waals Van der waals
33	Apigetrin	− 8.5	GLN178(2.71) HIS337(2.28) GLN178(2.13) HIS337(3.19) PRO500(3.15)	H-Bond H-Bond H-Bond Hydrophobic Hydrophobic

The molecular docking results for both the antioxidant protein target 1R4U and the anti-inflammatory protein target 3LN1 provide valuable insights into the binding interactions of the investigated ligands. In terms of antioxidant activity, the ligands Isoquercetin, Rutin, Procyanidin B2, Quercetrin, Kaempferol, Apigenin, and Quercetin-3-*O*-glucuronide were evaluated against the protein target 1R4U. These ligands demonstrated binding scores ranging from − 8.5 to − 9.4 kcal/mol. They formed interactions with specific residues such as THR74, ARG105, ARG128, ASP205, PRO76, CYS103, MET32, TYR30, and ARG105, among others. The presence of hydrogen bonds, hydrophobic interactions, and electrostatic interactions with varying distances suggests diverse binding modes and potential contributions to the antioxidant activity of the ligands. On the other hand, the ligands Isoquercetin, Rutin, Procyanidin B2, Quercetrin, Kaempferol, Apigenin, Quercetin-3-*O*-glucuronide, and Apigetrin were evaluated against the anti-inflammatory protein target 3LN1. Gallic acid interacts with TYR371, MET508, and ALA513 residues of the target protein. These interactions are likely mediated by hydrogen bonding, where the hydroxyl groups of Gallic acid form favorable bonds with specific amino acid side chains (e.g., hydroxyl groups of Tyr and Ser). Gallic acid also interacts with VAL335, GLY512, and LEU338 residues. These interactions are predominantly hydrophobic in nature, where the nonpolar parts of Gallic acid interact with nonpolar amino acid side chains. Protocatechuic acid interacts with THR192 and ALA188 residues of the target protein. The interaction with THR192 may involve hydrogen bonding, where the hydroxyl group of Protocatechuic acid forms a bond with the amino acid side chain. The interaction with HIS374 is likely a combination of hydrogen bonding and π-π stacking. The hydroxyl group of Protocatechuic acid may form a hydrogen bond with the side chain of HIS374, while the aromatic ring of Protocatechuic acid may stack on top of the aromatic ring of HIS374. These ligands demonstrated binding scores ranging from − 8.1 to − 9.2 kcal/mol. The interactions occurred with residues such as HIS193, HIS372, HIS374, ASN368, GLN440, GLU276, THR198, HIS200, and HIS337, among others. These interactions include hydrogen bonds and hydrophobic interactions, highlighting the potential involvement of specific residues in modulating the anti-inflammatory activity of the ligands.

When comparing the results, it is evident that some ligands demonstrated consistent binding affinity in both antioxidant and anti-inflammatory protein targets. Isoquercetin and Rutin exhibited similar binding scores in both targets (− 8.8 kcal/mol for 1R4U and − 8.1 kcal/mol for 3LN1 and − 9.2 kcal/mol for 1R4U and − 9.2 kcal/mol for 3LN1, respectively). This suggests that these ligands have the potential to exert combined antioxidant and anti-inflammatory effects^71^. Furthermore, there were variations in the residues involved in the binding interactions between the two protein targets. For instance, THR74, ARG128, and ASP205 were specific residues in the antioxidant protein target 1R4U, while HIS193, HIS372, and GLN440 were specific residues in the anti-inflammatory protein target 3LN1. These differences in interaction patterns highlight the specificity of ligand–protein interactions and the importance of understanding target-specific binding modes for designing ligands with desired antioxidant and anti-inflammatory properties^72^. In general, while some ligands exhibited consistent binding affinity in both targets, there were also variations in the specific residues involved. These findings emphasize the importance of considering target specificity when designing ligands with dual antioxidant and anti-inflammatory activities or when targeting specific pathways related to oxidative stress and inflammation.

## Conclusion

In this study, we examined the efficacy of an aqueous extract derived from the leaves of *C. laurifolius* in terms of its ability to scavenge free radicals, reduce inflammation, and relieve pain. The results of our study revealed that the plant extract possessed potent antioxidant capabilities, as evidenced by its low IC_50_ values in DPPH (2.92 µg/mL), ABTS (2.48 µg/mL), and RP (2.59 µg/mL) assays. Additionally, the extract exhibited remarkable anti-inflammatory properties (82%) that slightly surpassed those of indomethacin at the tested dose of 500 mg/kg, a commonly used anti-inflammatory drug. Moreover, the extract demonstrated both peripheral and central analgesic effects, reducing the nociceptive effect of acetic acid (48%) and increasing pain tolerance.

These pharmacological benefits of the plant extract can be attributed to the high levels of phenolic compounds identified in the leaf extract using colorimetric and UPLC-ESI–MS/MS methods. Furthermore, the molecular docking analysis of the investigated ligands against antioxidant protein target 1R4U and anti-inflammatory protein target 3LN1 provided valuable insights into their binding interactions, highlighting specific residues and interactions involved in the binding process. These findings shed light on the potential dual antioxidant and anti-inflammatory properties of certain ligands and contribute to our understanding of ligand–protein interactions.

The results of this study suggest that *C. laurifolius* has potential as a preventive and/or therapeutic agent for oxidative stress, inflammation, and pain relief. These findings have implications for future drug development efforts targeting oxidative stress and inflammation-related pathways.

### Supplementary Information

Supplementary Information 1.

Supplementary Tables.

## Data Availability

All data generated or analysed during this study are included in this published article.

## Abbreviations

AAE: Ascorbic acid equivalent.
ABTS: 2,2′-Azino-bis (3-ethylbenzothiazoline-6-sulfonic acid).
BHT: Butyl hydroxy toluene.
DPPH: 2,2-Diphenyl-1- picrylhydrazyl.
EC: Equivalent of catechin.
EQ: Equivalent of quercetin.
ESI: Electrospray ionization interface.
GAE: Gallic acid equivalent.
MRM: Multiple reaction monitoring.
PC: Proanthocyanidin content.
PPM: Phosphomolybdate.
RMSD: Root-mean-square deviation.
ROS: Reactive oxygen species.
RP: Reducing potential.
TAC: Total antioxidant capacity.
TFC: Total flavonoid content.
TPC: Total Phenolic content.
UPLC: Ultra-performance liquid chromatography
