# Supplementary material for: Ionomic analysis, polyphenols characterization, analgesic, antiinflammatory and antioxidant capacities of Cistus laurifolius leaves: in vitro, in vivo, and in silico investigations
Source: Sci Rep. 2023 Dec 21;13:22890. doi: 10.1038/s41598-023-50031-5 (PMC10739726; doi:10.1038/s41598-023-50031-5)

Dataset: W:\QACL\personeel\Stijn\MEET@ALL\Polyfenolen\TargetLynx\Resultaten\20220803 Toufik.qld

Last Altered: Monday, August 08, 2022 08:07:15 Romance (zomertijd)
Printed: Thursday, November 17, 2022 11:26:49 Romance (standaardtijd)

Name: 20220803_037, Date: 04-Aug-2022, Time: 05:26:14, ID: , Description: P 2

Daidzin

20220803_037 Smooth(SG,2x3) F20:MRM of 2 channels,ES-
P 2 253 > 224
1.986e+006

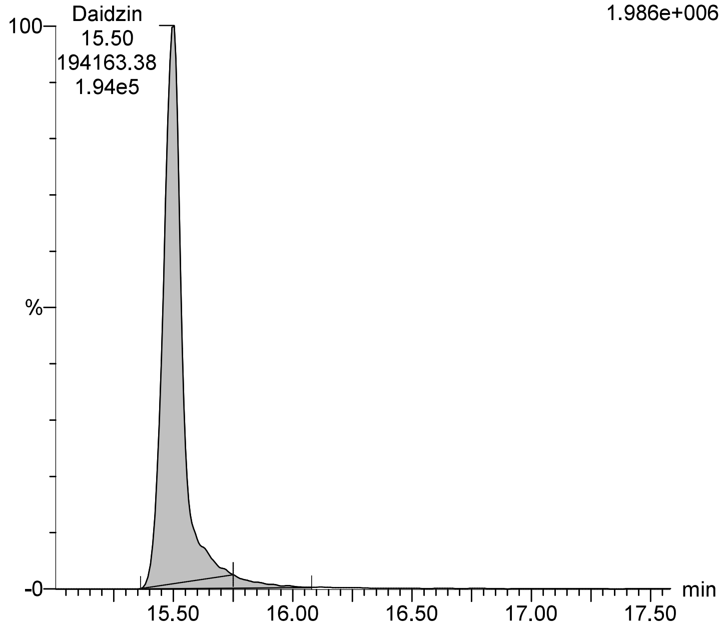

Pyrocatechol

20220803_037 Smooth(SG,2x3) F1:MRM of 2 channels,ES-
P 2 109.2 > 108.896
1.929e+006

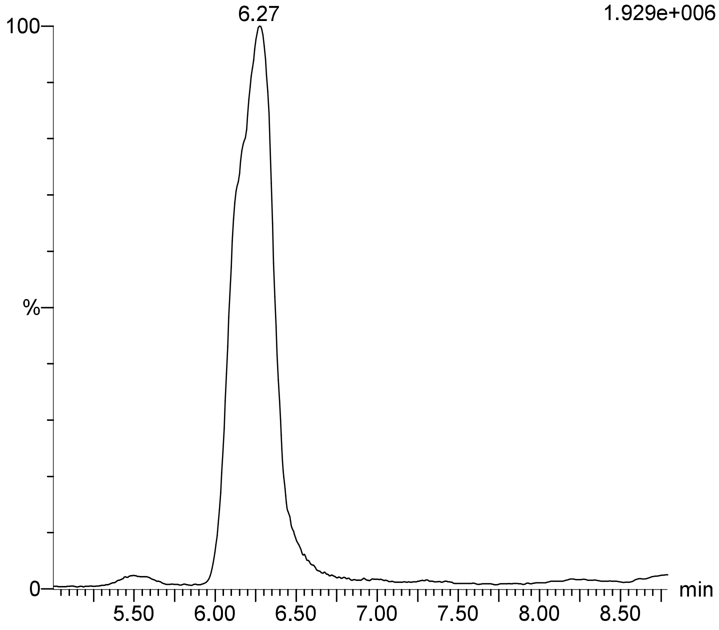

Pyrocatechol

20220803_037 Smooth(SG,2x3) F1:MRM of 2 channels,ES-
P 2 109.2 > 80.93
8.687e+004

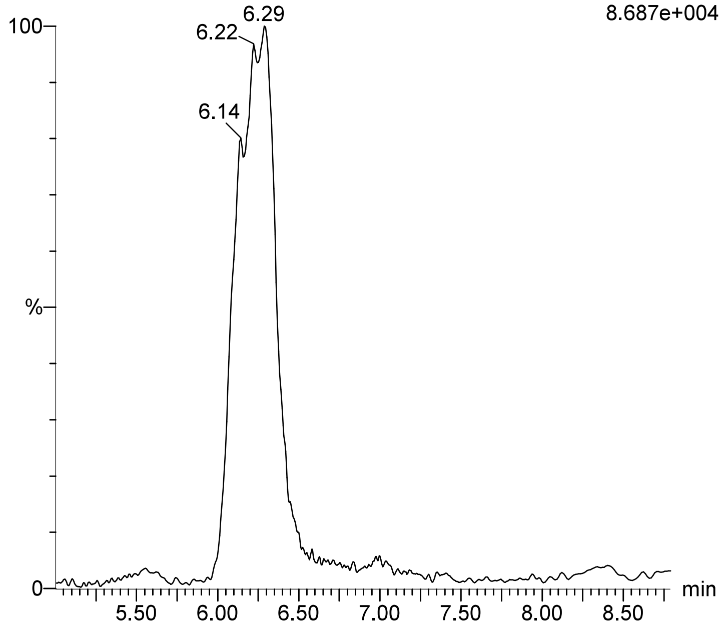

Salicylic acid

20220803_037 Smooth(SG,2x3) F2:MRM of 2 channels,ES-
P 2 137 > 93
5.532e+005

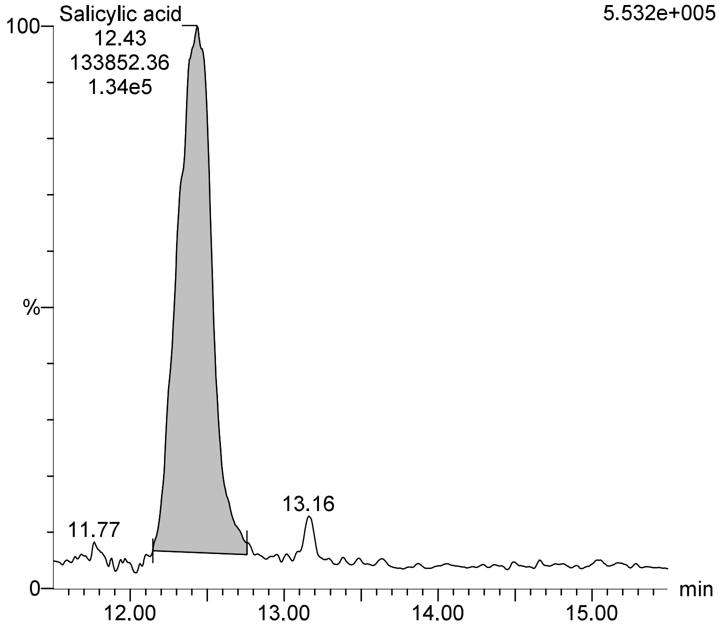

Dataset: W:\QACL\personeel\Stijn\MEET@ALL\Polyfenolen\TargetLynx\Resultaten\20220803 Toufik.qld

Last Altered: Monday, August 08, 2022 08:07:15 Romance (zomertijd)
Printed: Thursday, November 17, 2022 11:26:49 Romance (standaardtijd)

Name: 20220803_037, Date: 04-Aug-2022, Time: 05:26:14, ID: , Description: P 2

Salicylic acid

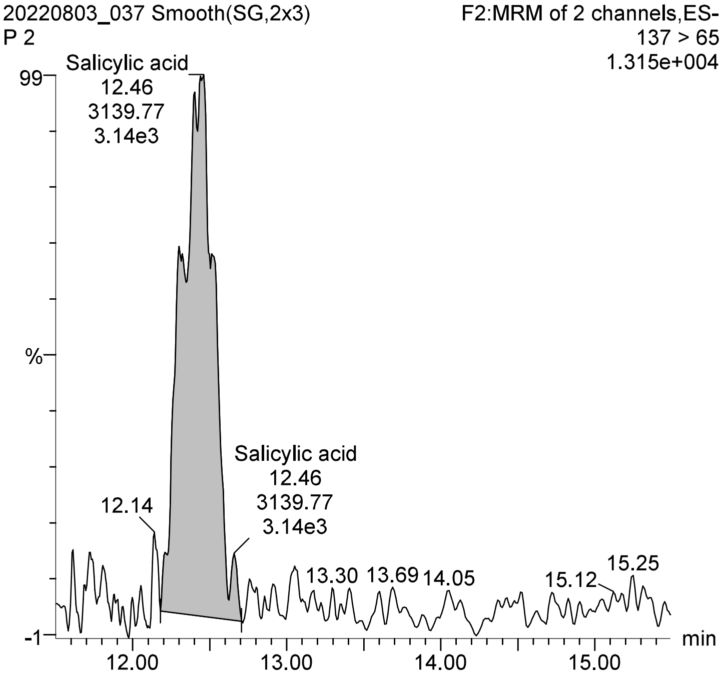

4-OH-phenylacetic acid

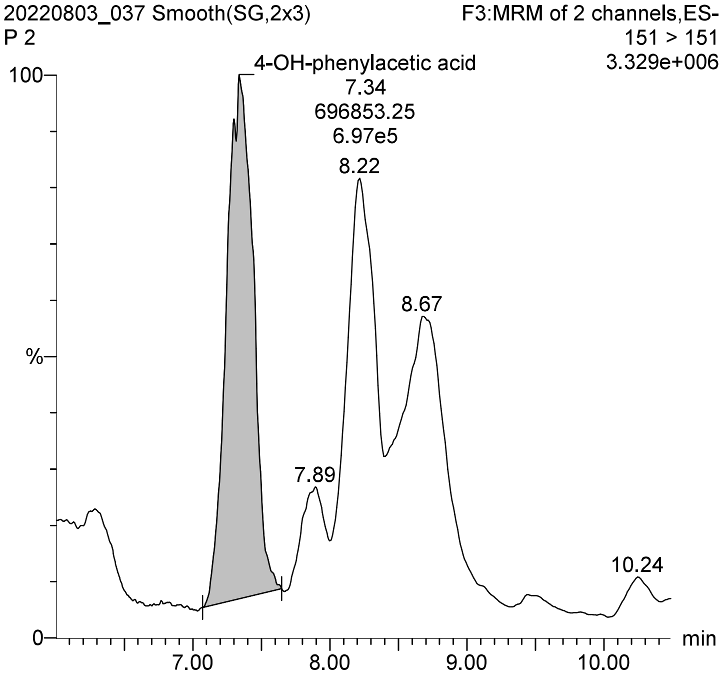

4-OH-phenylacetic acid

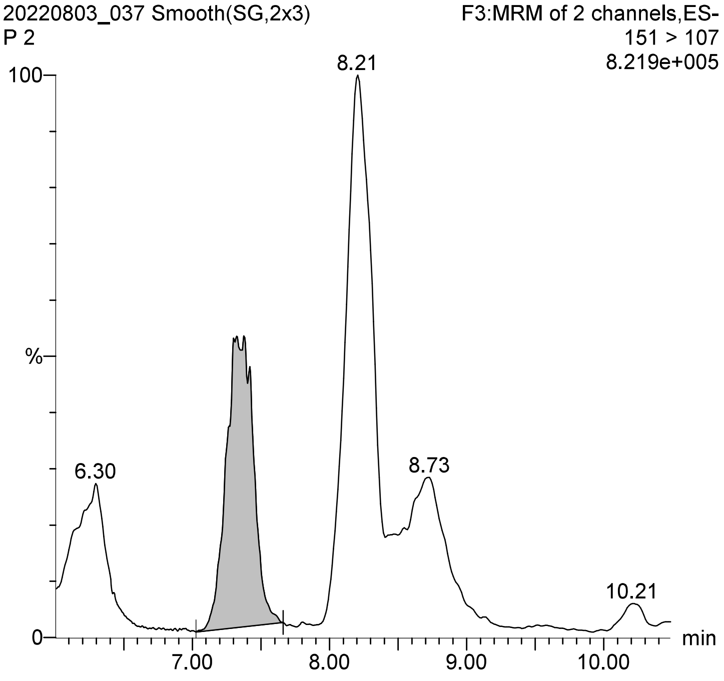

Protocatechuic acid

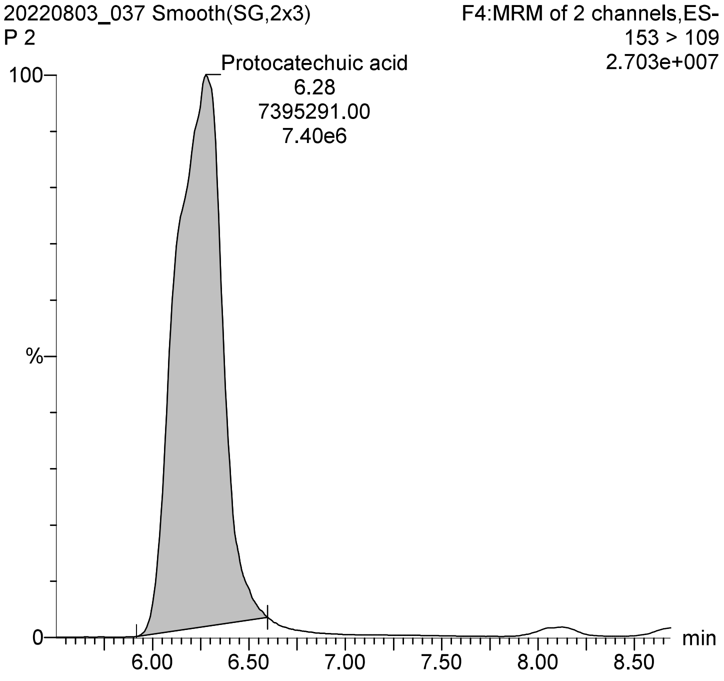

Dataset: W:\QACL\personeel\Stijn\MEET@ALL\Polyfenolen\TargetLynx\Resultaten\20220803 Toufik.qld

Last Altered: Monday, August 08, 2022 08:07:15 Romance (zomertijd)
Printed: Thursday, November 17, 2022 11:26:49 Romance (standaardtijd)

Name: 20220803_037, Date: 04-Aug-2022, Time: 05:26:14, ID: , Description: P 2

Protocatechuic acid

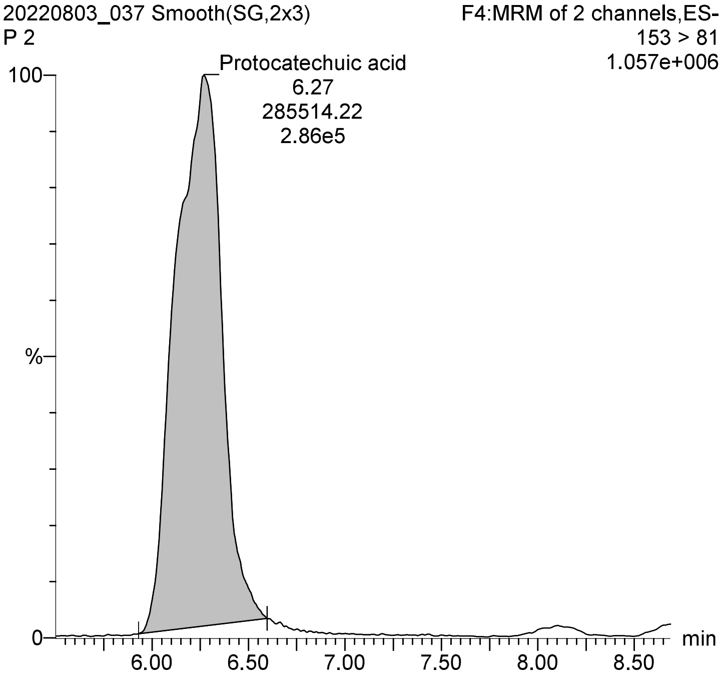

Gentisic

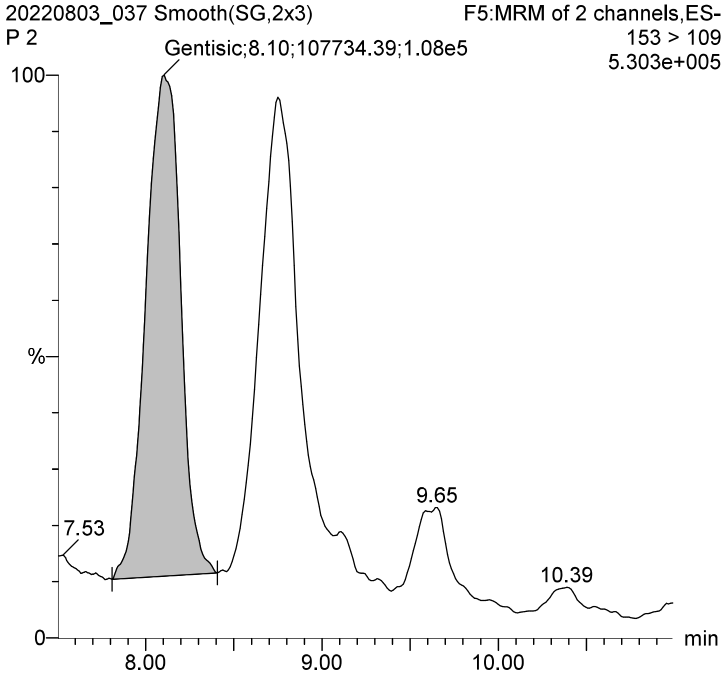

Gentisic

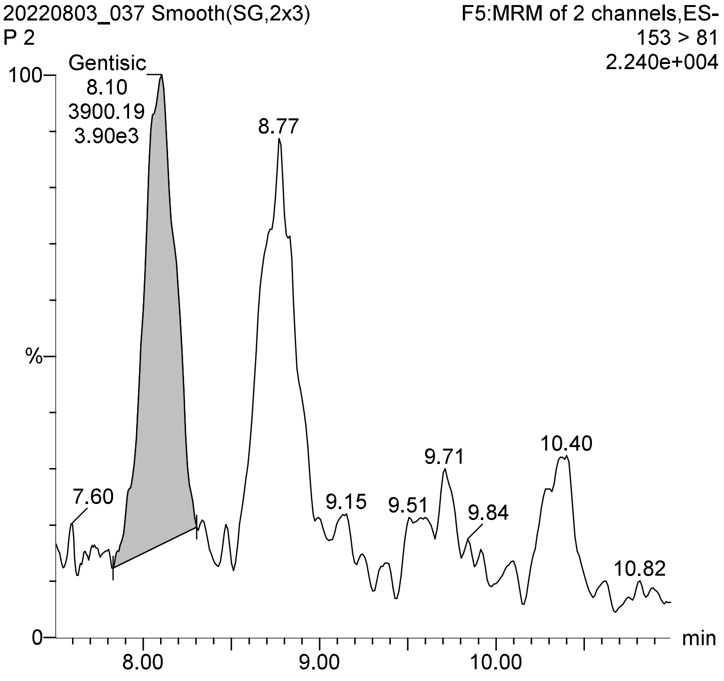

p-Coumaric acid

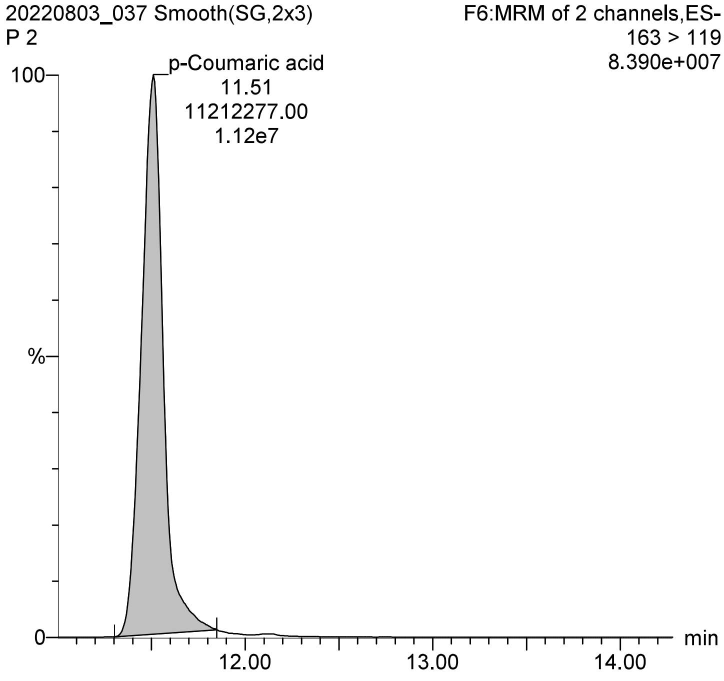

Dataset: W:\QACL\personeel\Stijn\MEET@ALL\Polyfenolen\TargetLynx\Resultaten\20220803 Toufik.qld

Last Altered: Monday, August 08, 2022 08:07:15 Romance (zomertijd)
Printed: Thursday, November 17, 2022 11:26:49 Romance (standaardtijd)

Name: 20220803_037, Date: 04-Aug-2022, Time: 05:26:14, ID: , Description: P 2

p-Coumaric acid

20220803_037 Smooth(SG,2x3) F6:MRM of 2 channels,ES-
P 2 163 > 93
3.662e+006

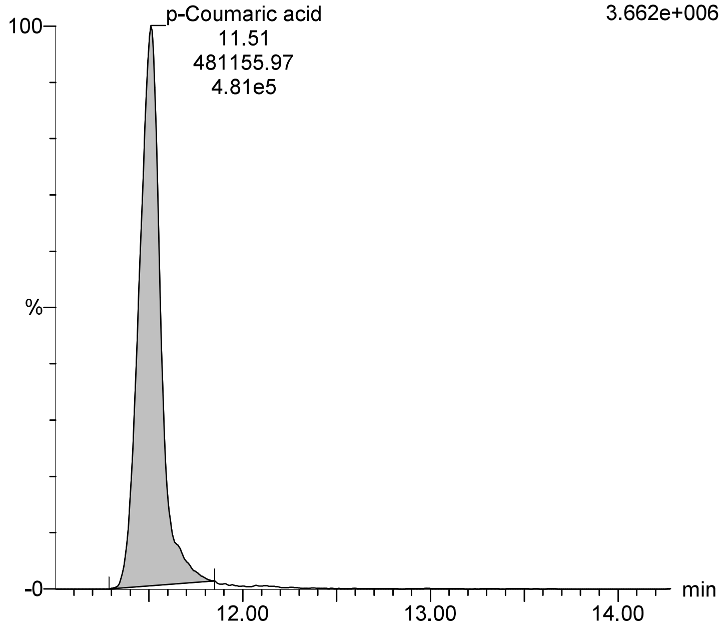

o-Coumaric acid

20220803_037 Smooth(SG,2x3) F6:MRM of 2 channels,ES-
P 2 163 > 119
8.390e+007

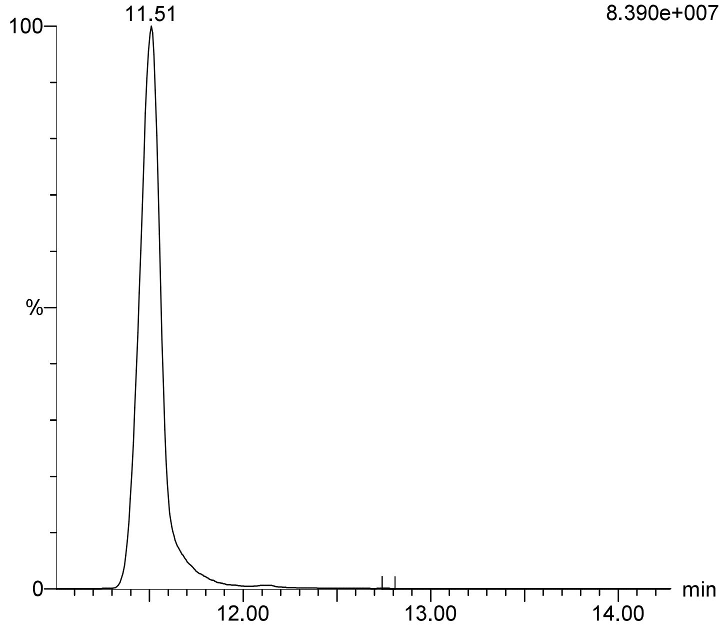

o-Coumaric acid

20220803_037 Smooth(SG,2x3) F6:MRM of 2 channels,ES-
P 2 163 > 93
3.662e+006

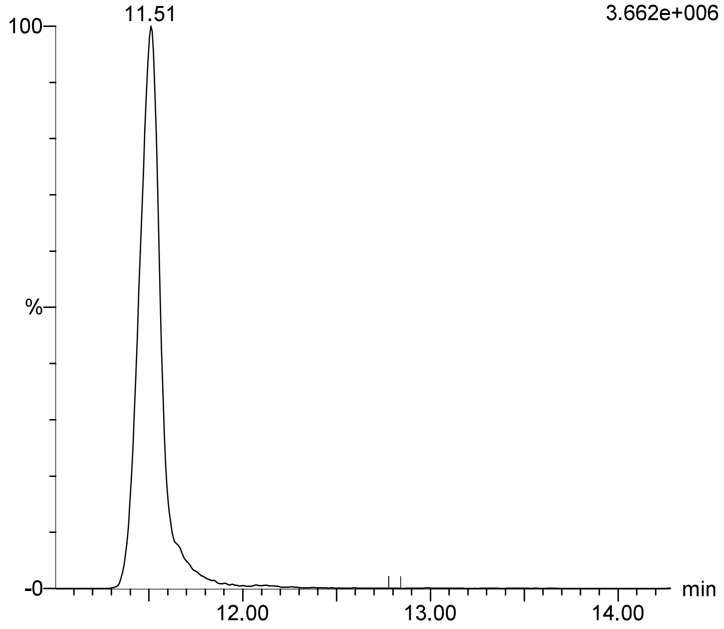

Vanillic acid

20220803_037 Smooth(SG,2x3) F8:MRM of 2 channels,ES-
P 2 167 > 152
1.100e+006

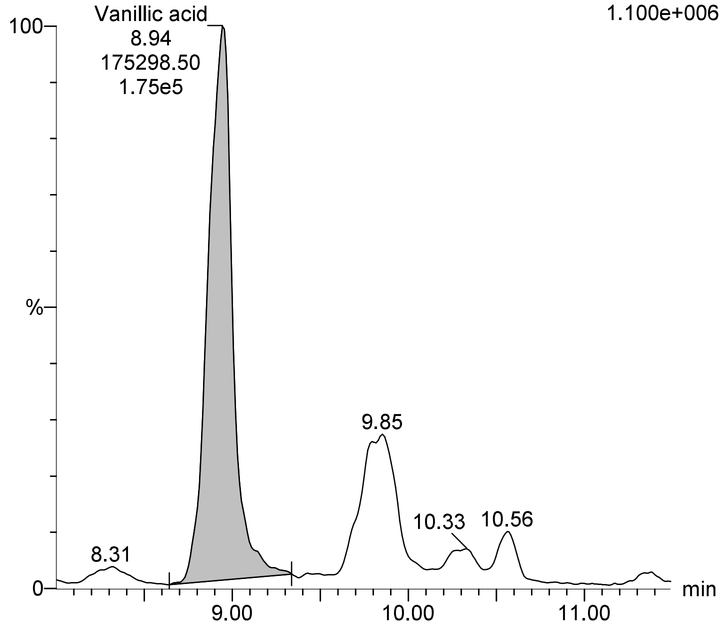

Dataset: W:\QACL\personeel\Stijn\MEET@ALL\Polyfenolen\TargetLynx\Resultaten\20220803 Toufik.qld

Last Altered: Monday, August 08, 2022 08:07:15 Romance (zomertijd)
Printed: Thursday, November 17, 2022 11:26:49 Romance (standaardtijd)

Name: 20220803_037, Date: 04-Aug-2022, Time: 05:26:14, ID: , Description: P 2

Vanillic acid

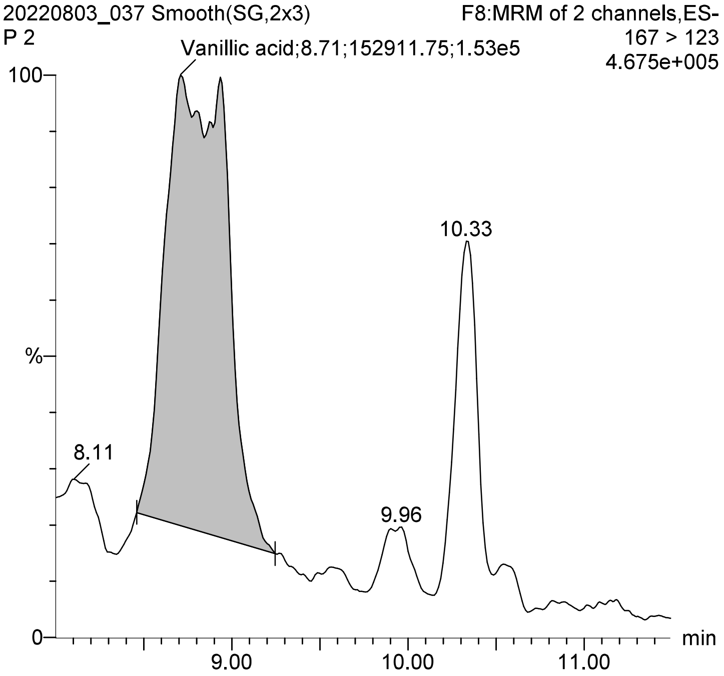

Gallic acid

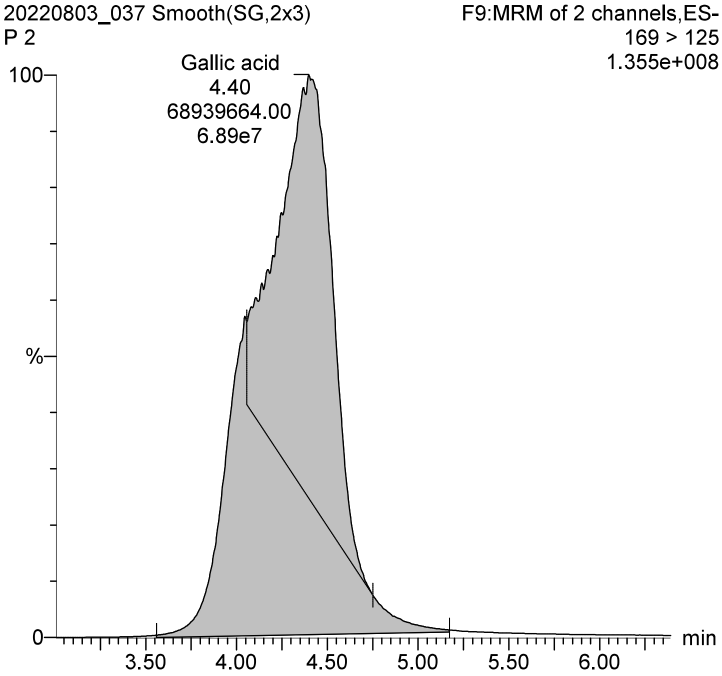

Gallic acid

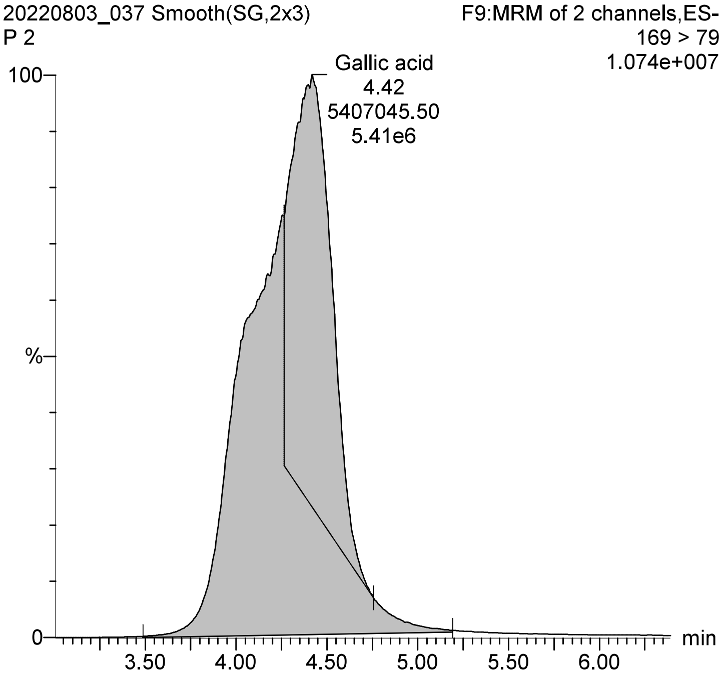

Caffeic acid

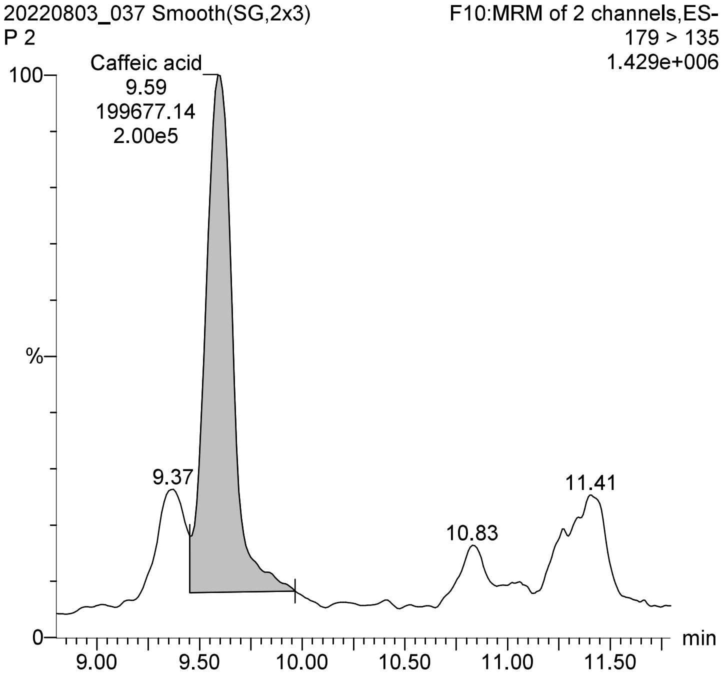

Dataset: W:\QACL\personeel\Stijn\MEET@ALL\Polyfenolen\TargetLynx\Resultaten\20220803 Toufik.qld

Last Altered: Monday, August 08, 2022 08:07:15 Romance (zomertijd)
Printed: Thursday, November 17, 2022 11:26:49 Romance (standaardtijd)

Name: 20220803_037, Date: 04-Aug-2022, Time: 05:26:14, ID: , Description: P 2

Caffeic acid

20220803_037 Smooth(SG,2x3) F10:MRM of 2 channels,ES-
P 2 179 > 107
3.438e+004

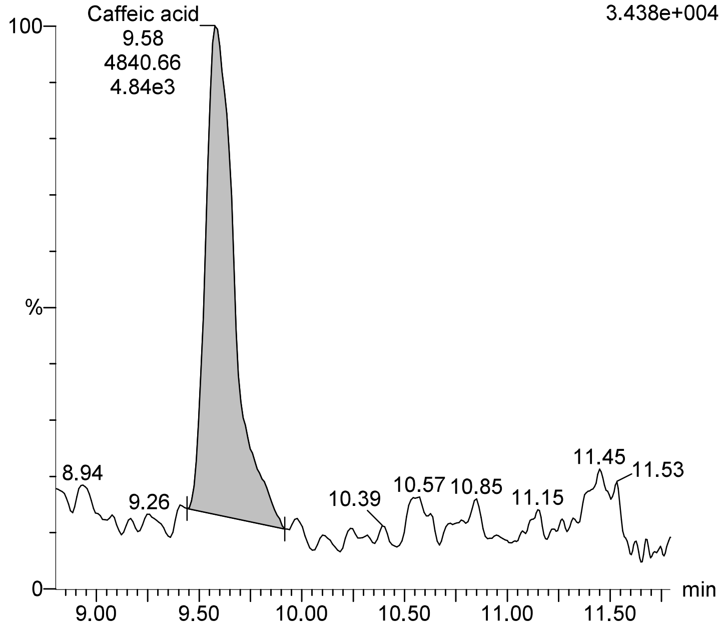

Hydrocaffeic acid

20220803_037 Smooth(SG,2x3) F11:MRM of 2 channels,ES-
P 2 181 > 137
4.005e+005

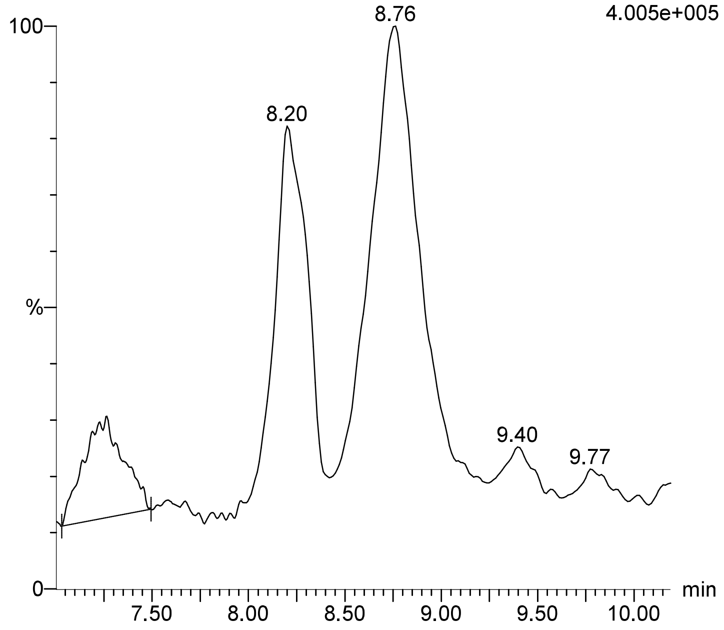

Hydrocaffeic acid

20220803_037 Smooth(SG,2x3) F11:MRM of 2 channels,ES-
P 2 181 > 109
1.462e+004

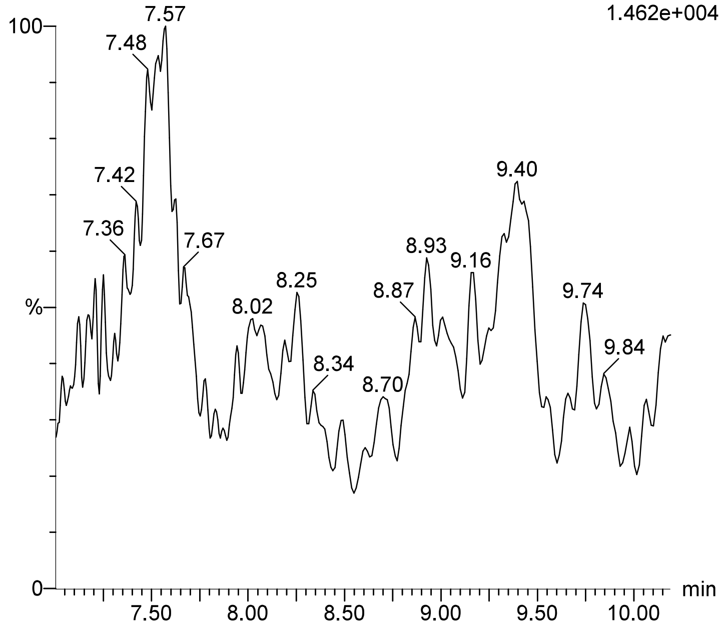

Quinic acid

20220803_037 Smooth(SG,2x3) F12:MRM of 2 channels,ES-
P 2 191 > 85
2.548e+006

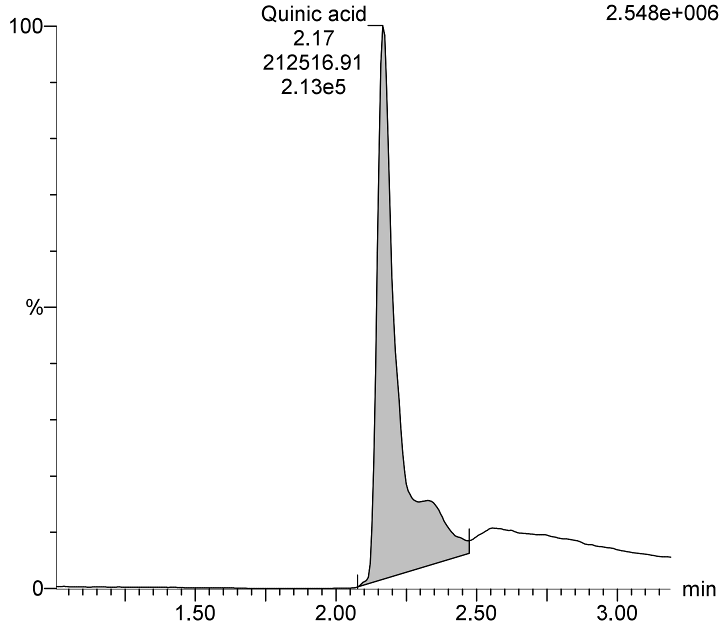

Dataset: W:\QACL\personeel\Stijn\MEET@ALL\Polyfenolen\TargetLynx\Resultaten\20220803 Toufik.qld

Last Altered: Monday, August 08, 2022 08:07:15 Romance (zomertijd)
Printed: Thursday, November 17, 2022 11:26:49 Romance (standaardtijd)

Name: 20220803_037, Date: 04-Aug-2022, Time: 05:26:14, ID: , Description: P 2

Quinic acid

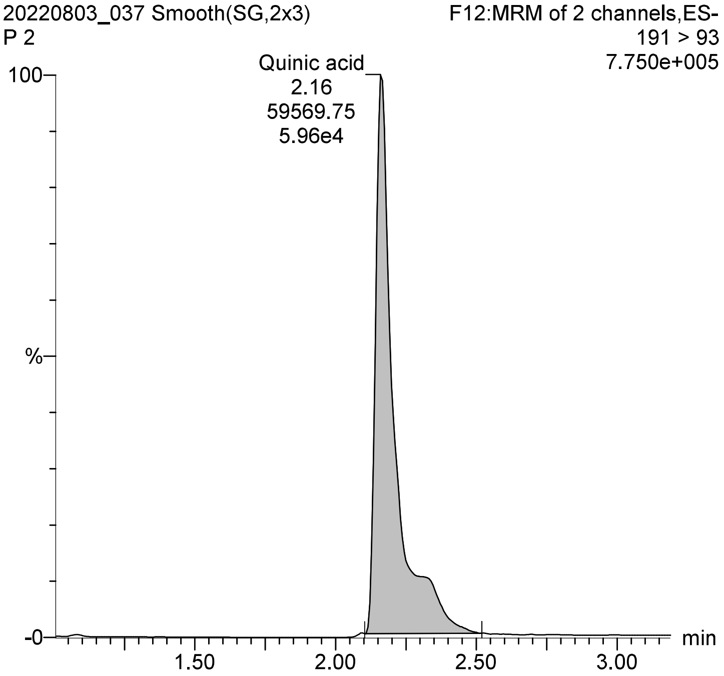

Ferulic acid

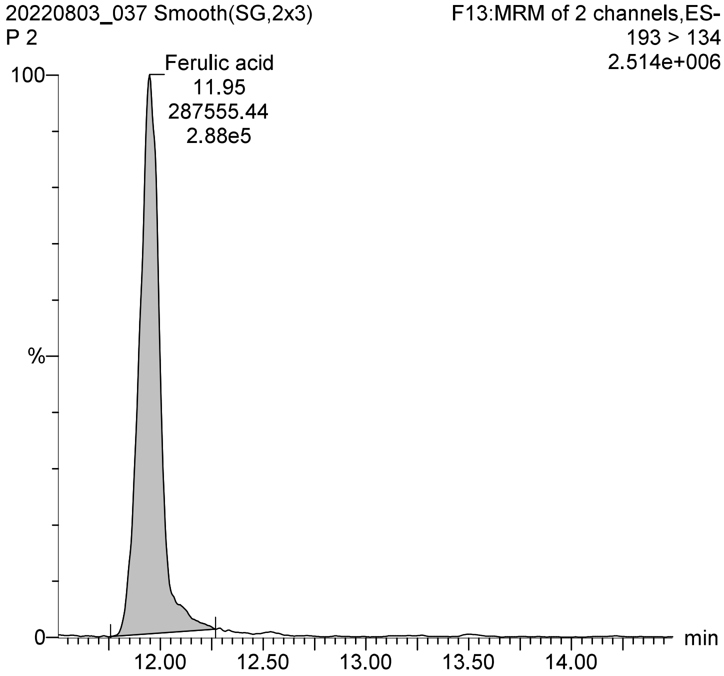

Ferulic acid

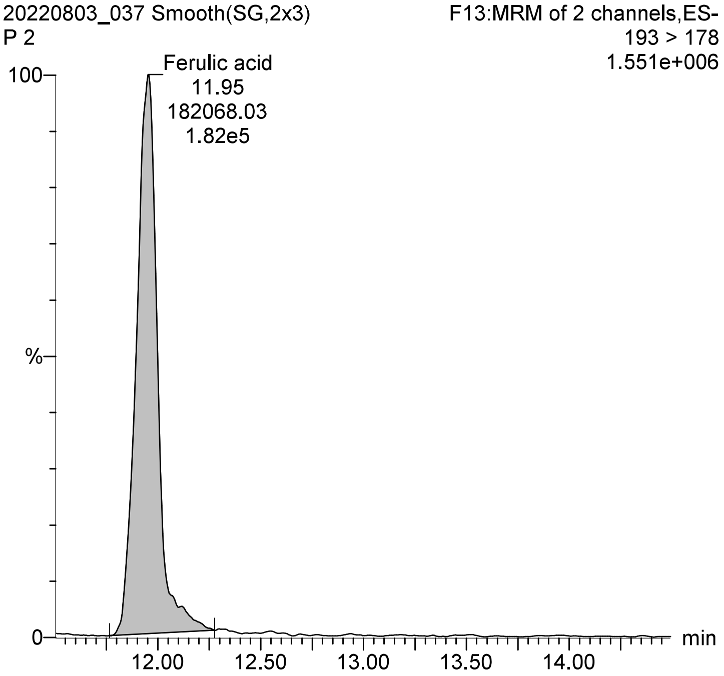

Hydroferulic acid

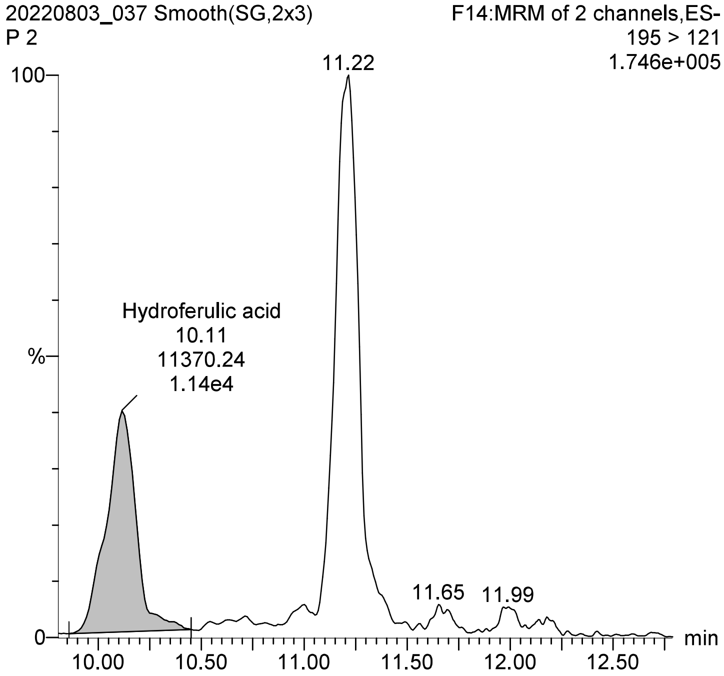

Dataset: W:\QACL\personeel\Stijn\MEET@ALL\Polyfenolen\TargetLynx\Resultaten\20220803 Toufik.qld

Last Altered: Monday, August 08, 2022 08:07:15 Romance (zomertijd)
Printed: Thursday, November 17, 2022 11:26:49 Romance (standaardtijd)

Name: 20220803_037, Date: 04-Aug-2022, Time: 05:26:14, ID: , Description: P 2

Hydroferulic acid

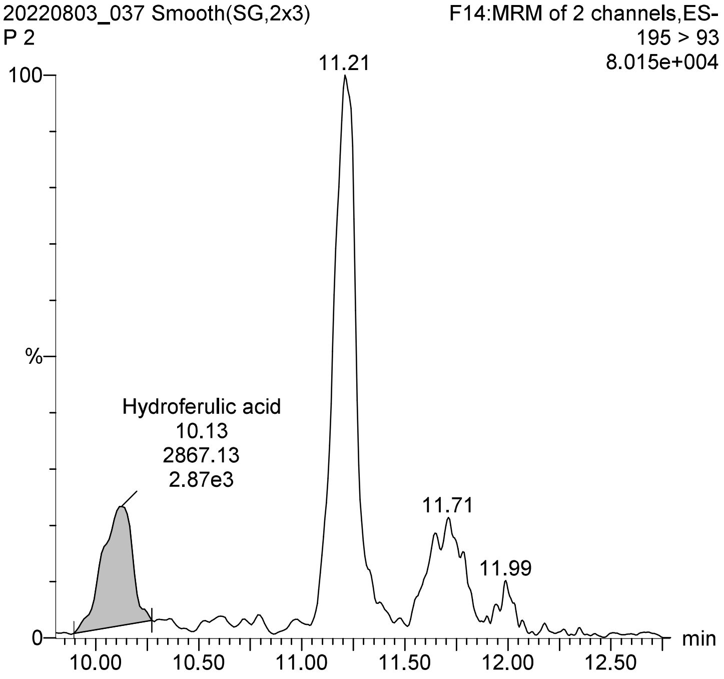

Propyl gallate

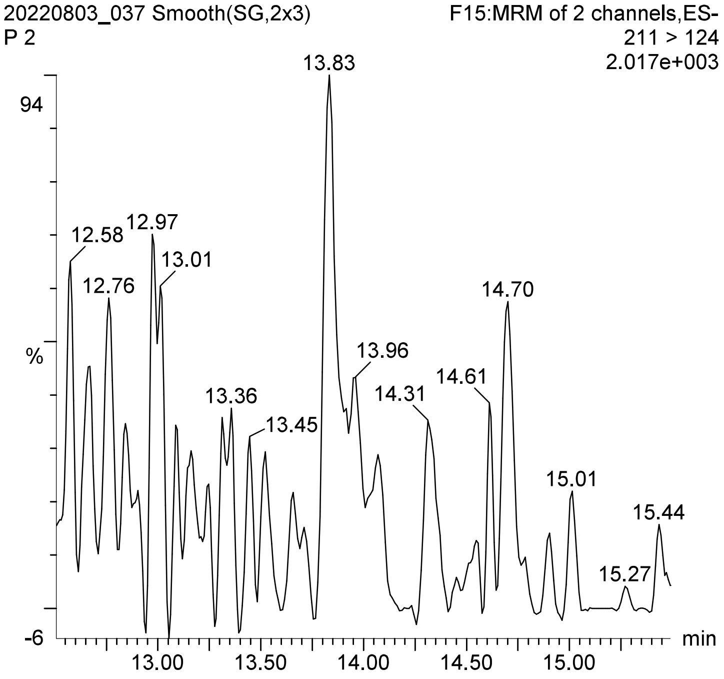

Propyl gallate

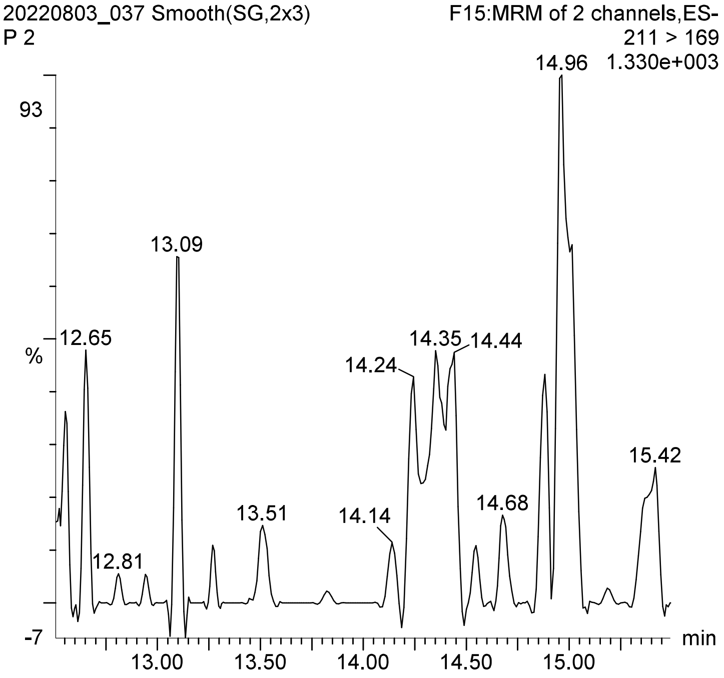

Sinapinic acid

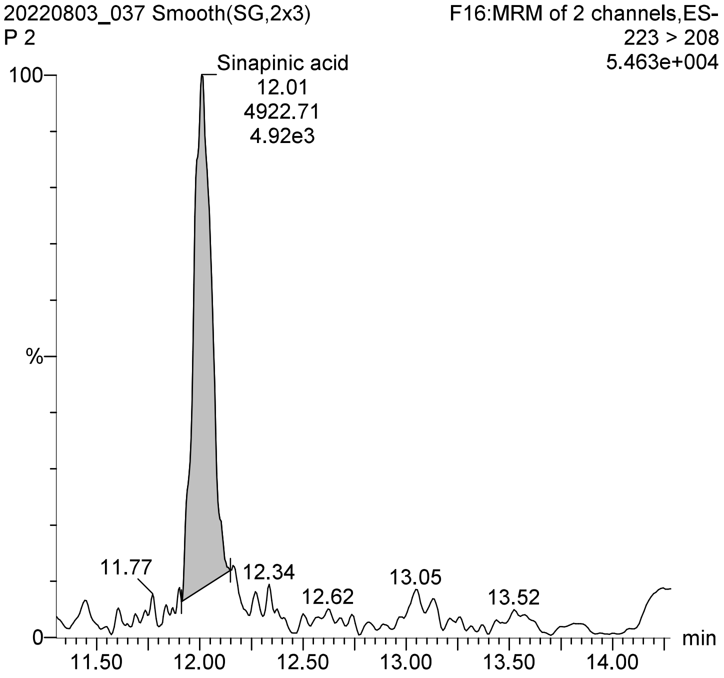

Dataset: W:\QACL\personeel\Stijn\MEET@ALL\Polyfenolen\TargetLynx\Resultaten\20220803 Toufik.qld

Last Altered: Monday, August 08, 2022 08:07:15 Romance (zomertijd)
Printed: Thursday, November 17, 2022 11:26:49 Romance (standaardtijd)

Name: 20220803_037, Date: 04-Aug-2022, Time: 05:26:14, ID: , Description: P 2

Sinapinic acid

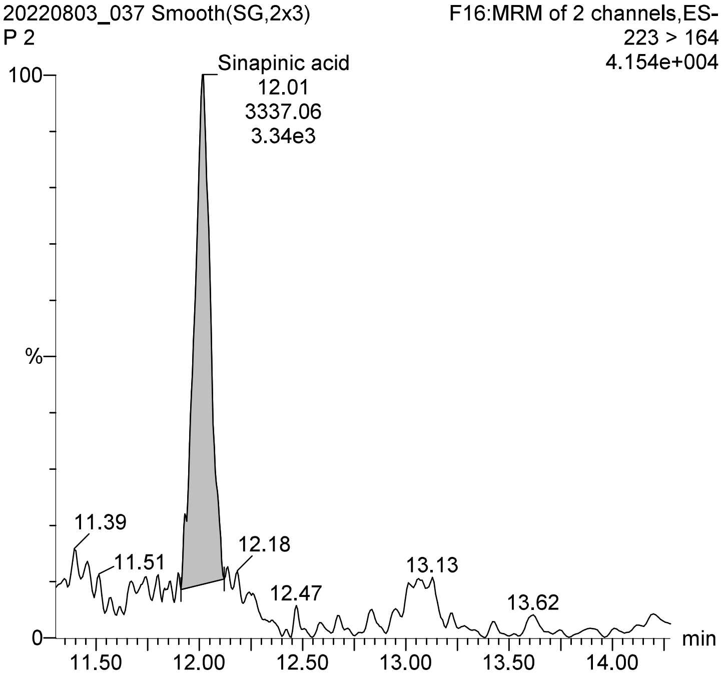

Resveratrol

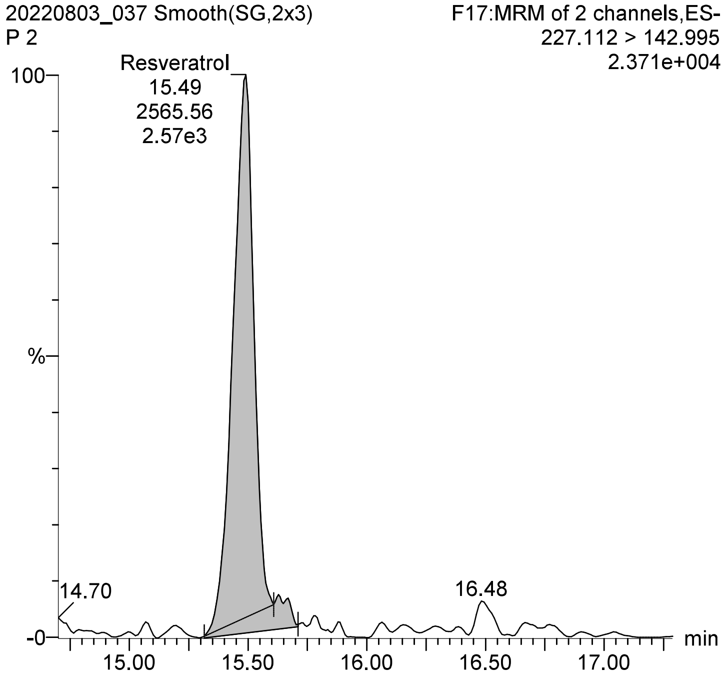

Resveratrol

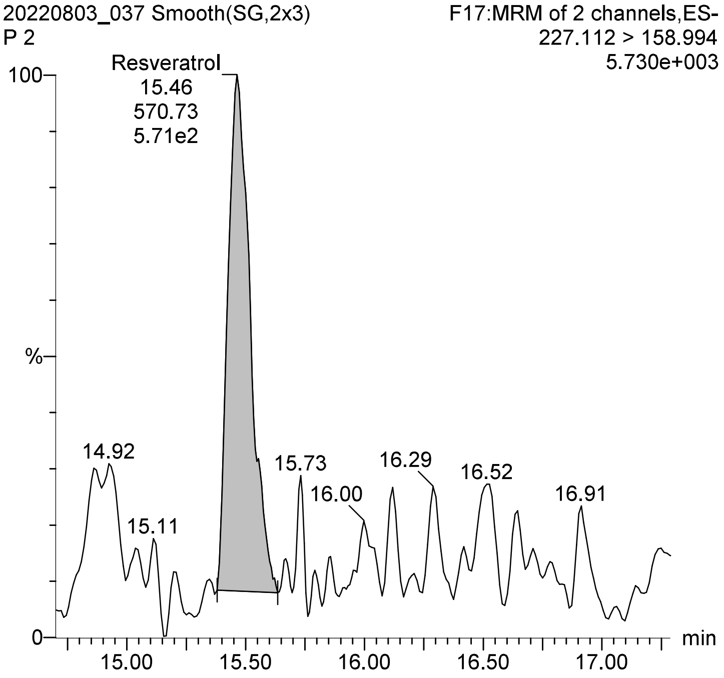

3,4,5 trimethoxycinnamic acid

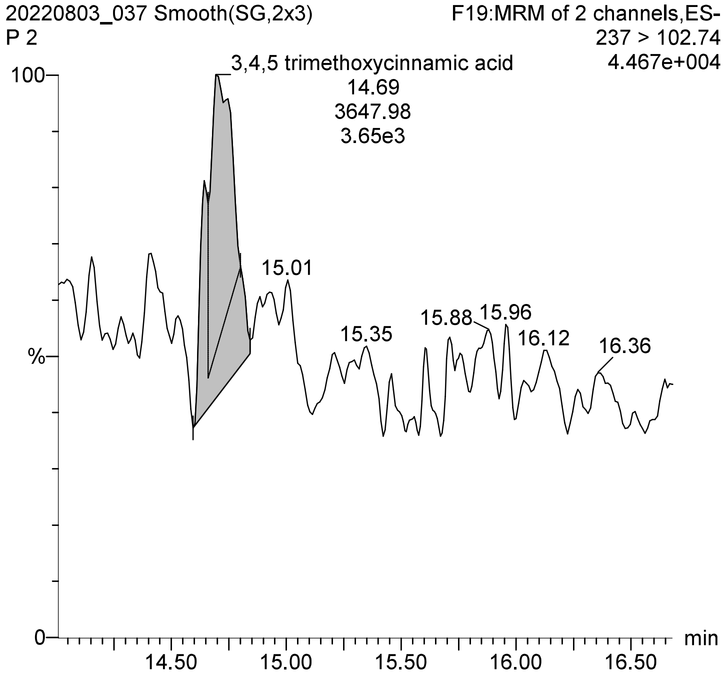

Dataset: W:\QACL\personeel\Stijn\MEET@ALL\Polyfenolen\TargetLynx\Resultaten\20220803 Toufik.qld

Last Altered: Monday, August 08, 2022 08:07:15 Romance (zomertijd)
Printed: Thursday, November 17, 2022 11:26:49 Romance (standaardtijd)

Name: 20220803_037, Date: 04-Aug-2022, Time: 05:26:14, ID: , Description: P 2

3,4,5 trimethoxycinnamic acid

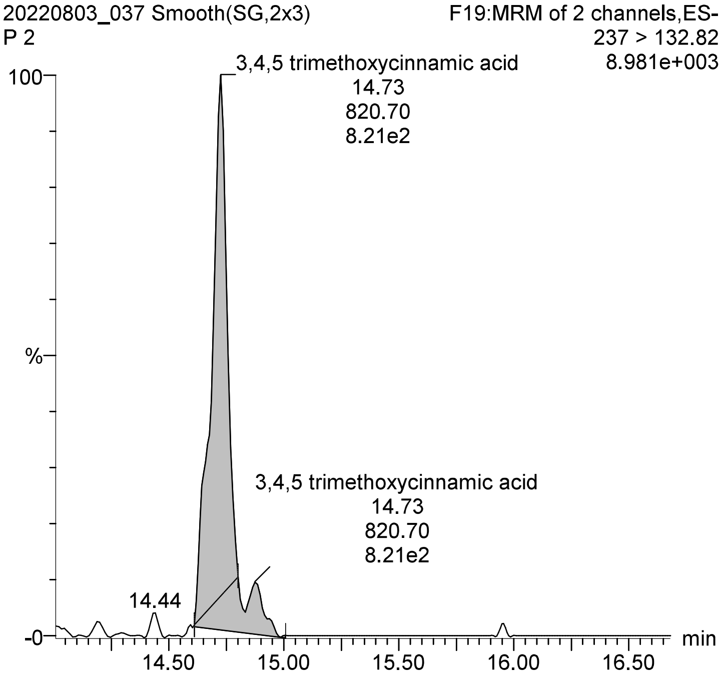

Galangin

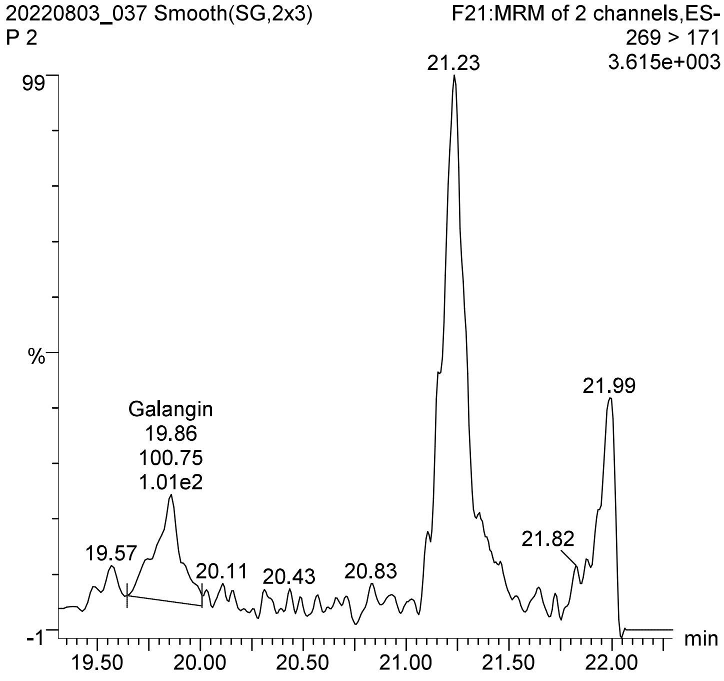

Galangin

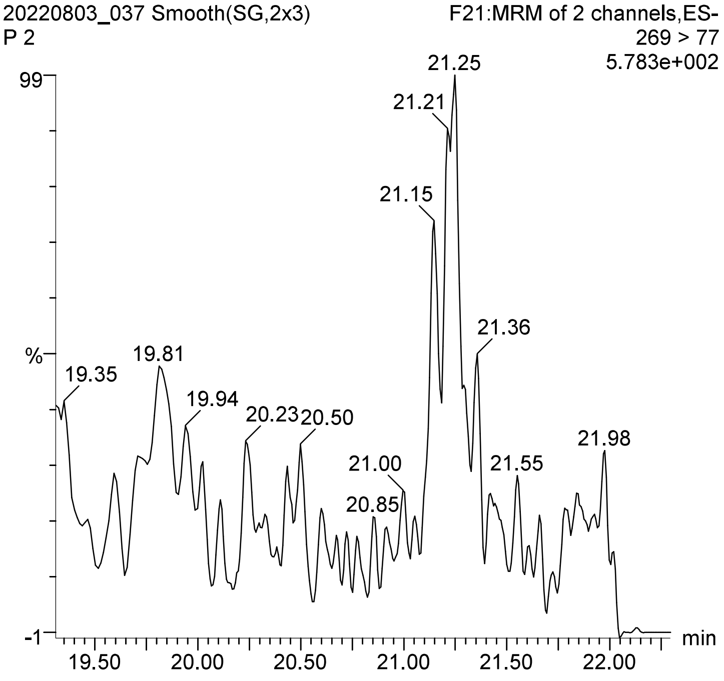

Apigenin

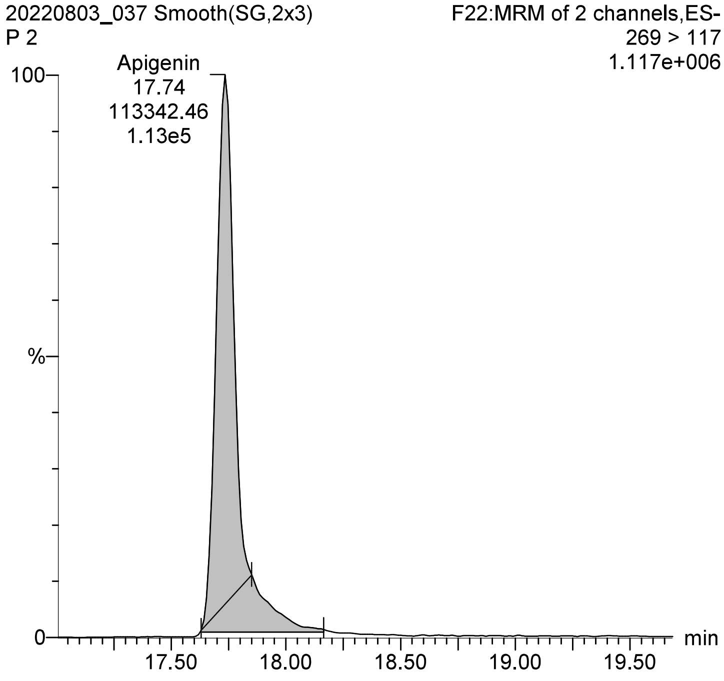

Dataset: W:\QACL\personeel\Stijn\MEET@ALL\Polyfenolen\TargetLynx\Resultaten\20220803 Toufik.qld

Last Altered: Monday, August 08, 2022 08:07:15 Romance (zomertijd)
Printed: Thursday, November 17, 2022 11:26:49 Romance (standaardtijd)

Name: 20220803_037, Date: 04-Aug-2022, Time: 05:26:14, ID: , Description: P 2

Apigenin

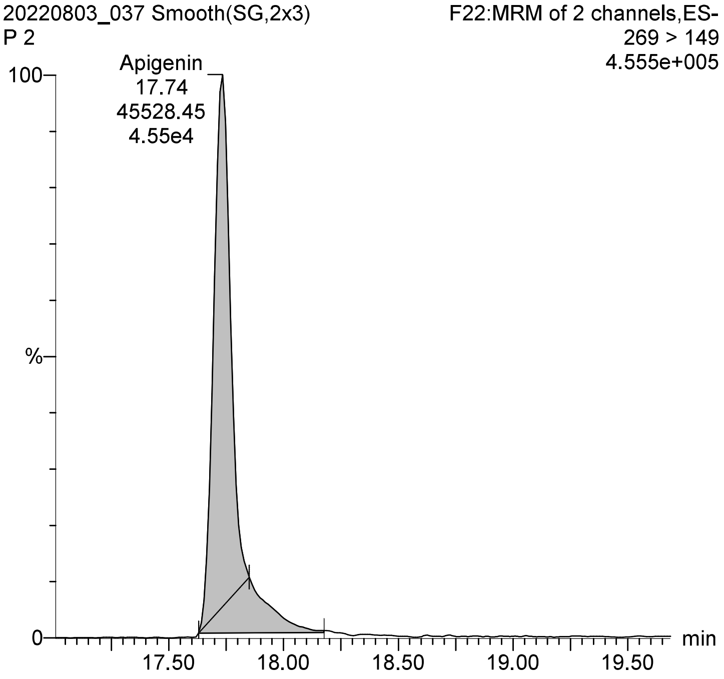

Naringenin

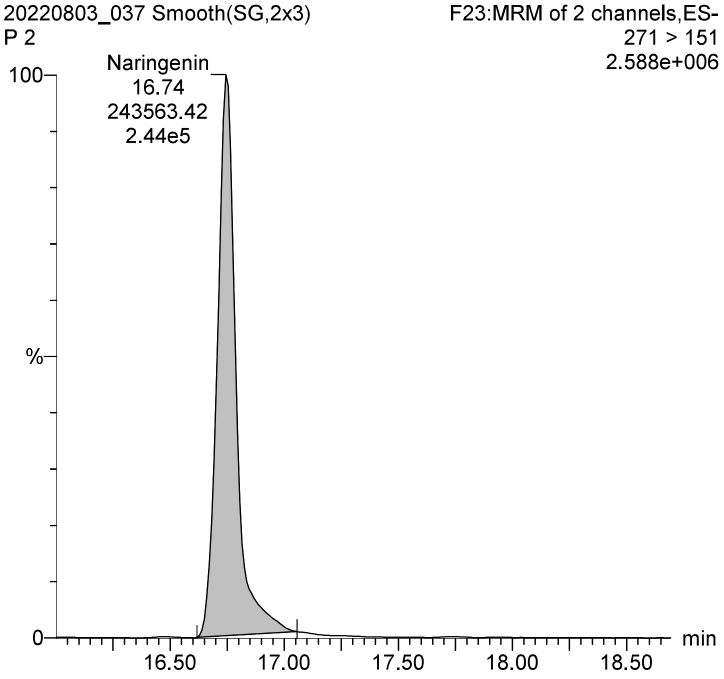

Naringenin

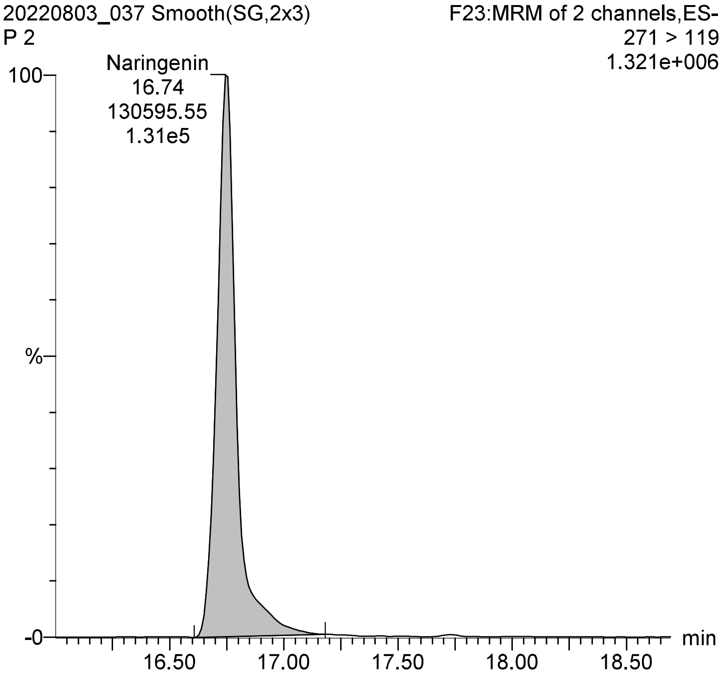

Phloretin

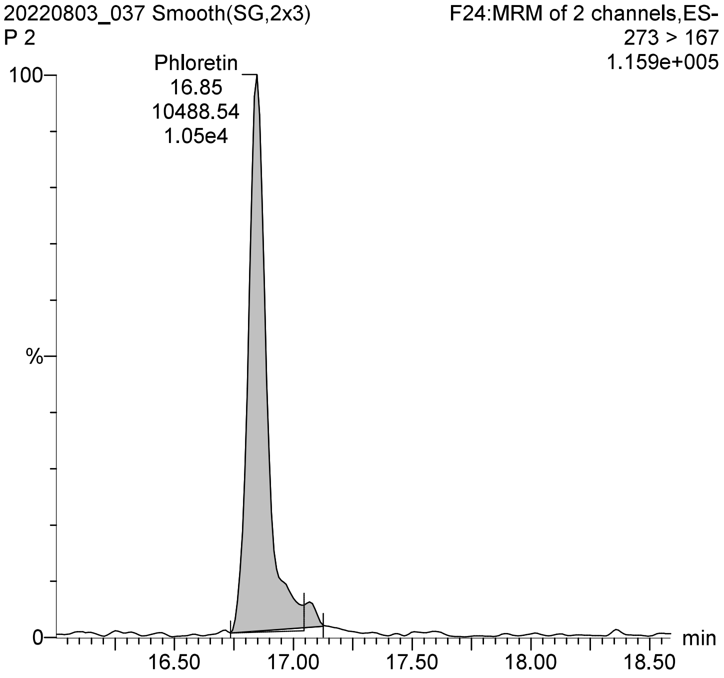

Dataset: W:\QACL\personeel\Stijn\MEET@ALL\Polyfenolen\TargetLynx\Resultaten\20220803 Toufik.qld

Last Altered: Monday, August 08, 2022 08:07:15 Romance (zomertijd)
Printed: Thursday, November 17, 2022 11:26:49 Romance (standaardtijd)

Name: 20220803_037, Date: 04-Aug-2022, Time: 05:26:14, ID: , Description: P 2

Phloretin

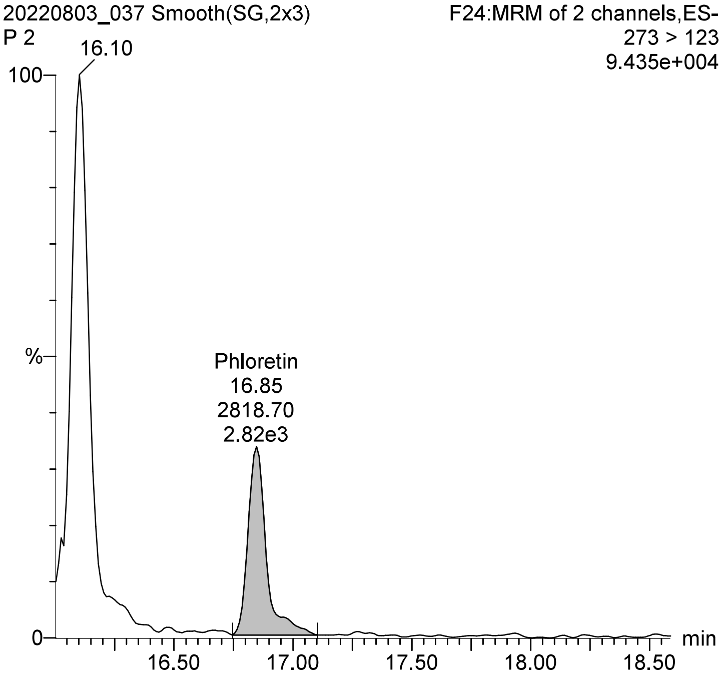

Kaempferol

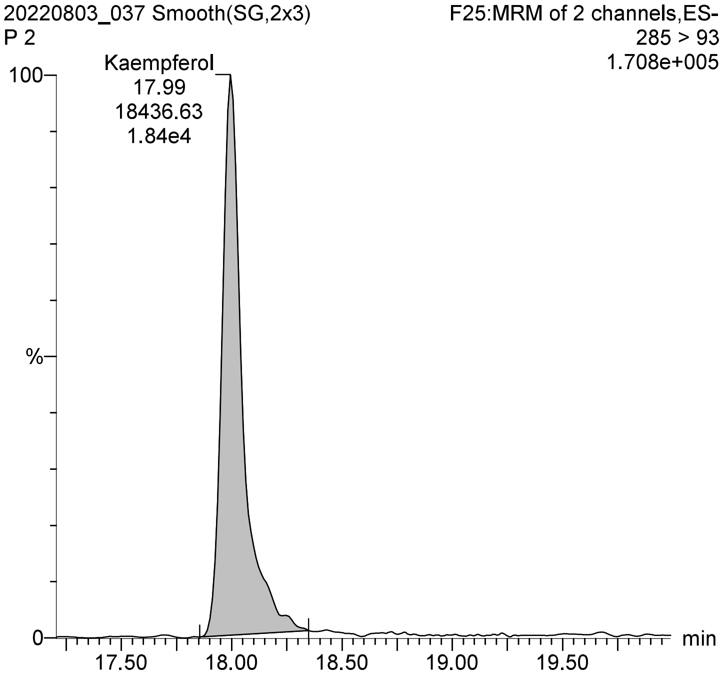

Kaempferol

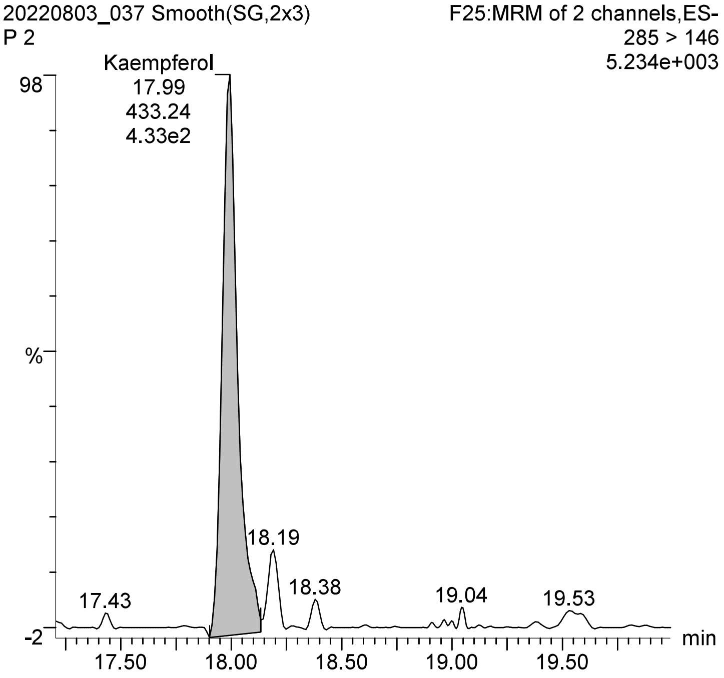

Luteolin

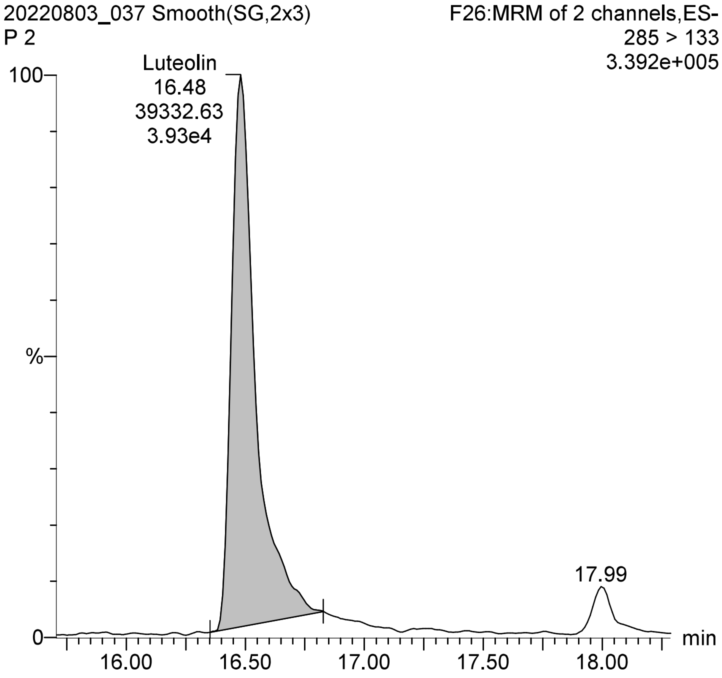

Dataset: W:\QACL\personeel\Stijn\MEET@ALL\Polyfenolen\TargetLynx\Resultaten\20220803 Toufik.qld

Last Altered: Monday, August 08, 2022 08:07:15 Romance (zomertijd)
Printed: Thursday, November 17, 2022 11:26:49 Romance (standaardtijd)

Name: 20220803_037, Date: 04-Aug-2022, Time: 05:26:14, ID: , Description: P 2

Luteolin

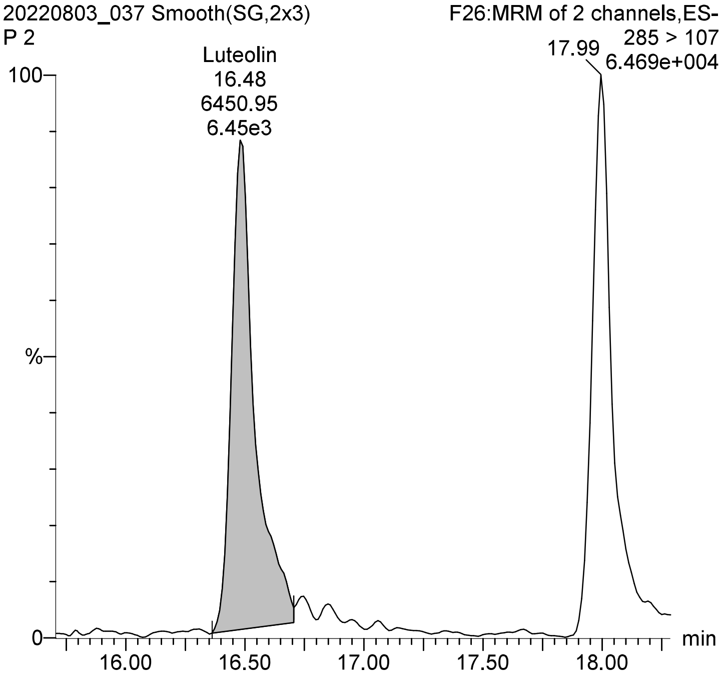

Aromadendrin

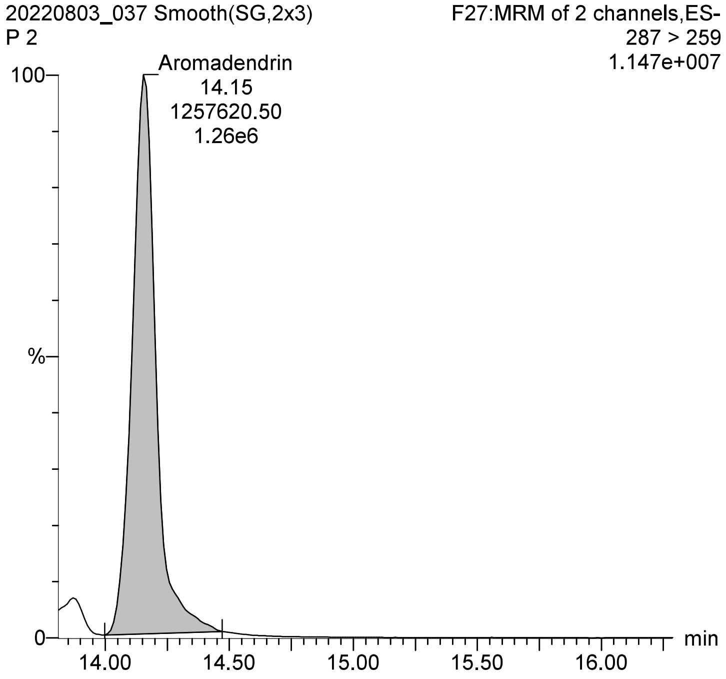

Aromadendrin

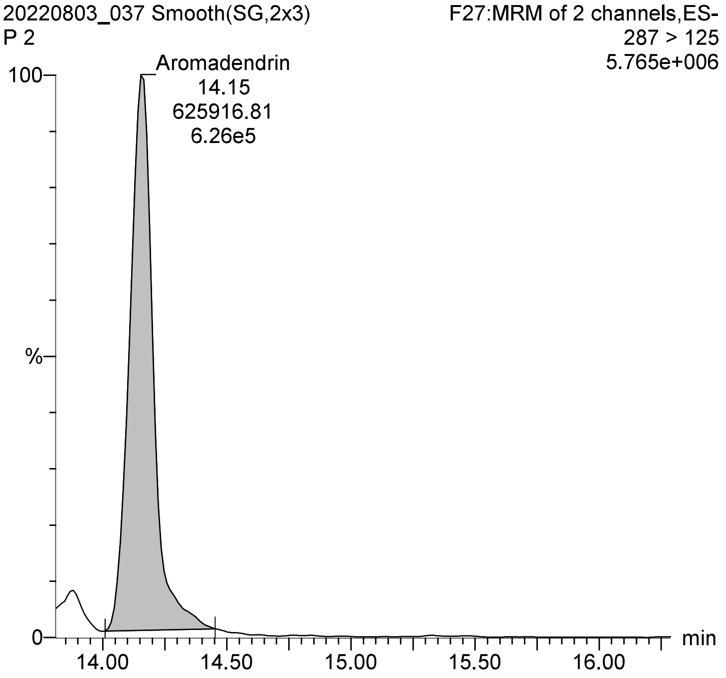

Epicatechin

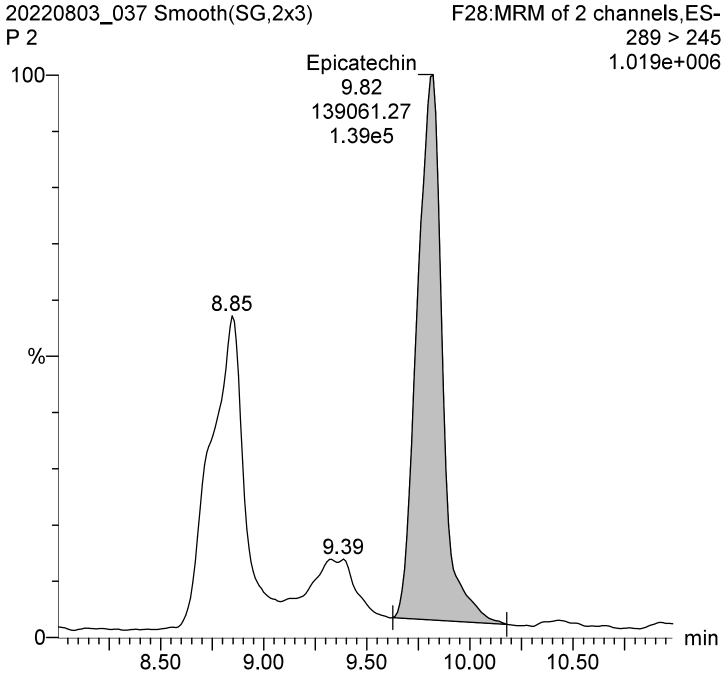

Dataset: W:\QACL\personeel\Stijn\MEET@ALL\Polyfenolen\TargetLynx\Resultaten\20220803 Toufik.qld

Last Altered: Monday, August 08, 2022 08:07:15 Romance (zomertijd)
Printed: Thursday, November 17, 2022 11:26:49 Romance (standaardtijd)

Name: 20220803_037, Date: 04-Aug-2022, Time: 05:26:14, ID: , Description: P 2

Epicatechin

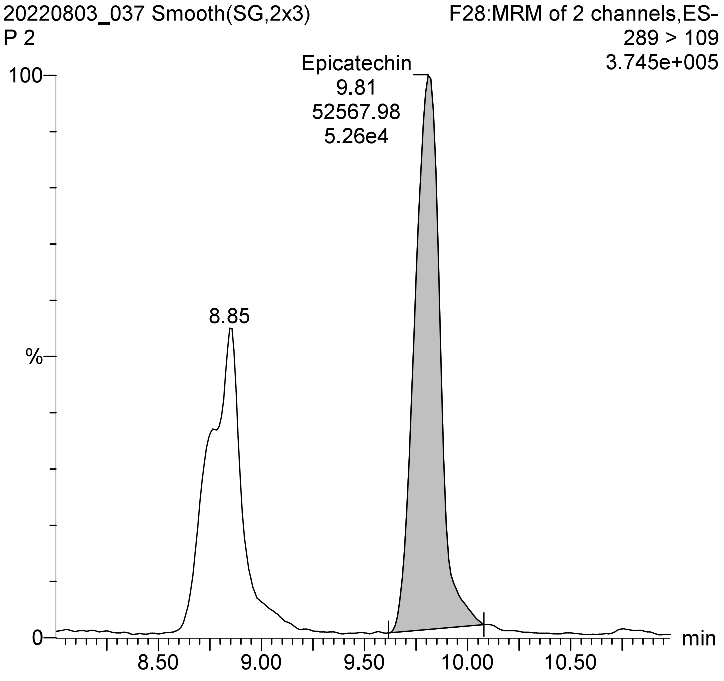

Catechin

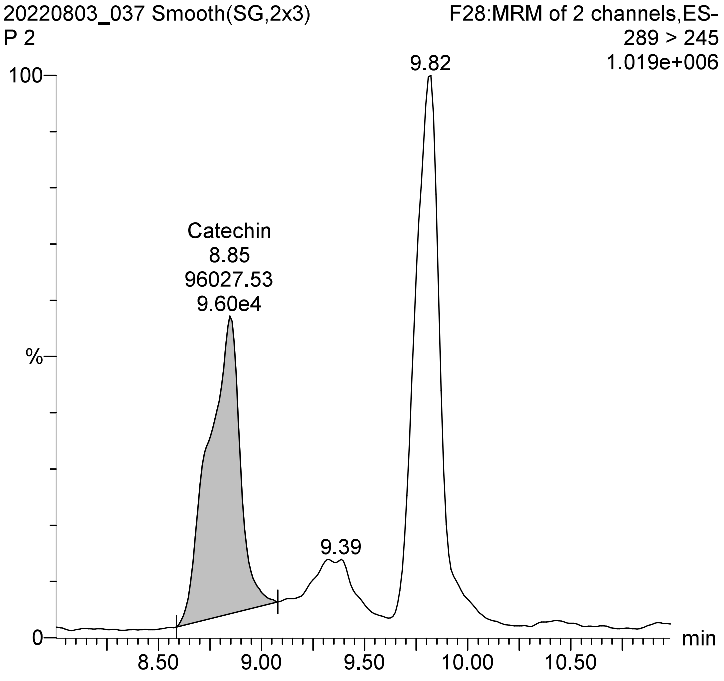

Catechin

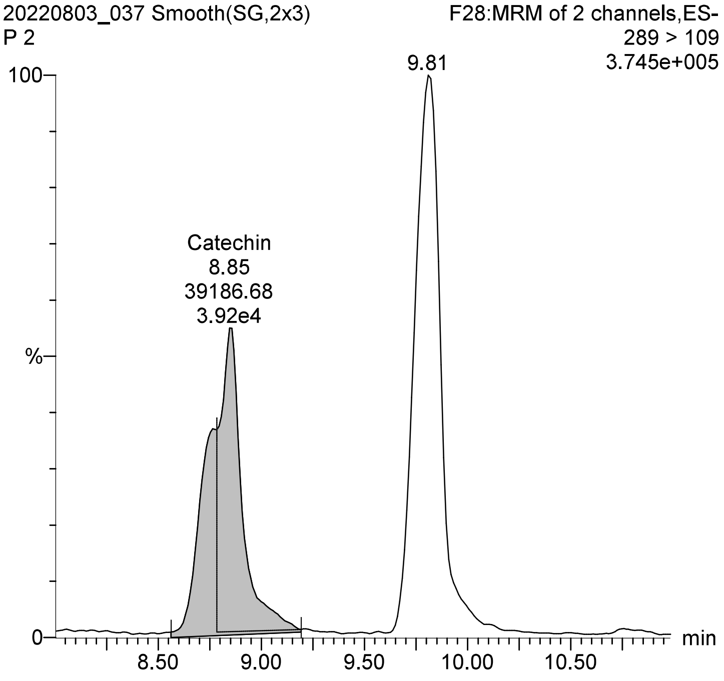

Quercetin

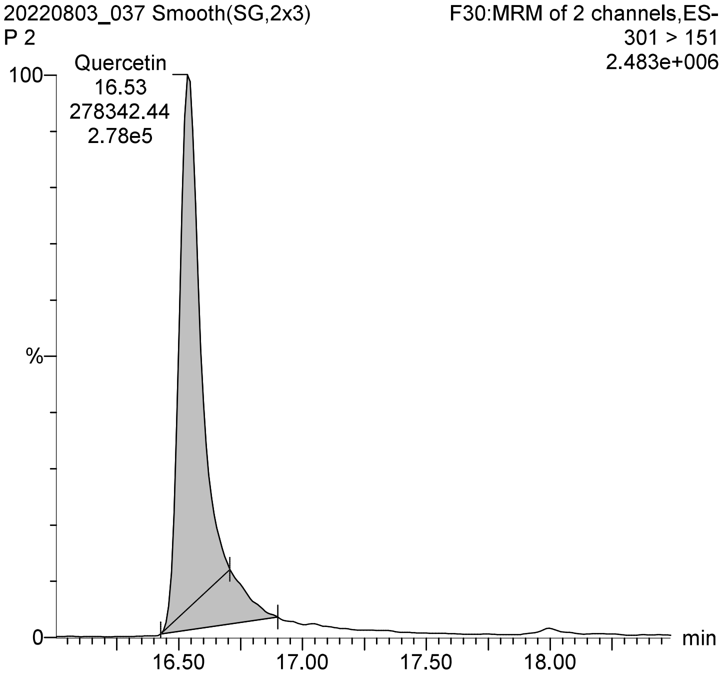

Dataset: W:\QACL\personeel\Stijn\MEET@ALL\Polyfenolen\TargetLynx\Resultaten\20220803 Toufik.qld

Last Altered: Monday, August 08, 2022 08:07:15 Romance (zomertijd)
Printed: Thursday, November 17, 2022 11:26:49 Romance (standaardtijd)

Name: 20220803_037, Date: 04-Aug-2022, Time: 05:26:14, ID: , Description: P 2

Quercetin

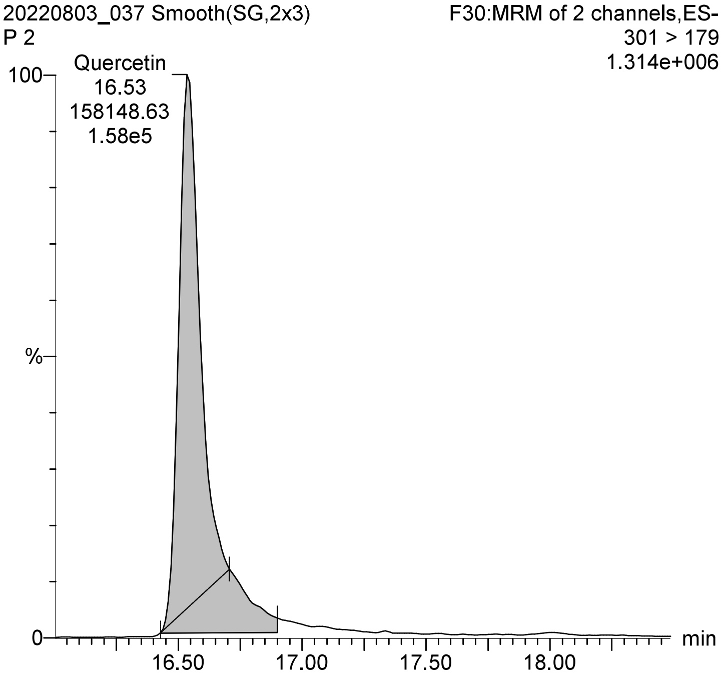

Hesperetin

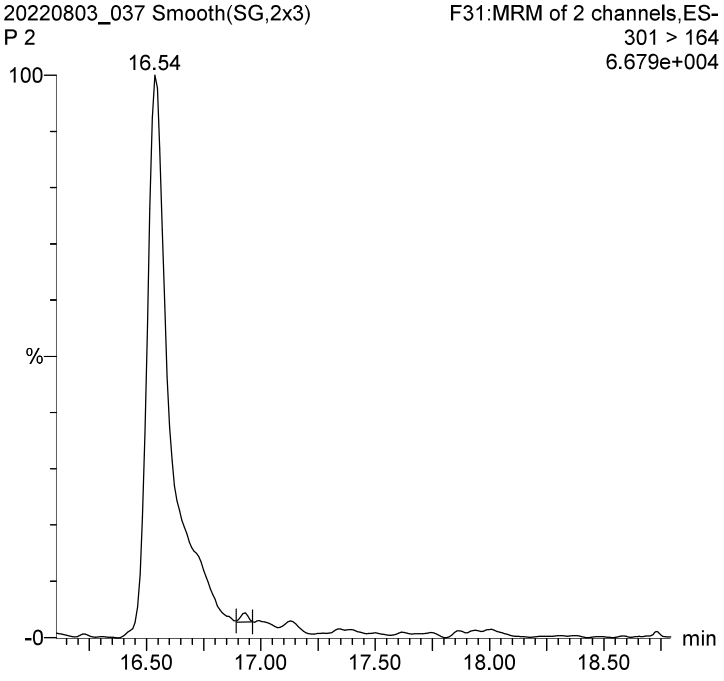

Hesperetin

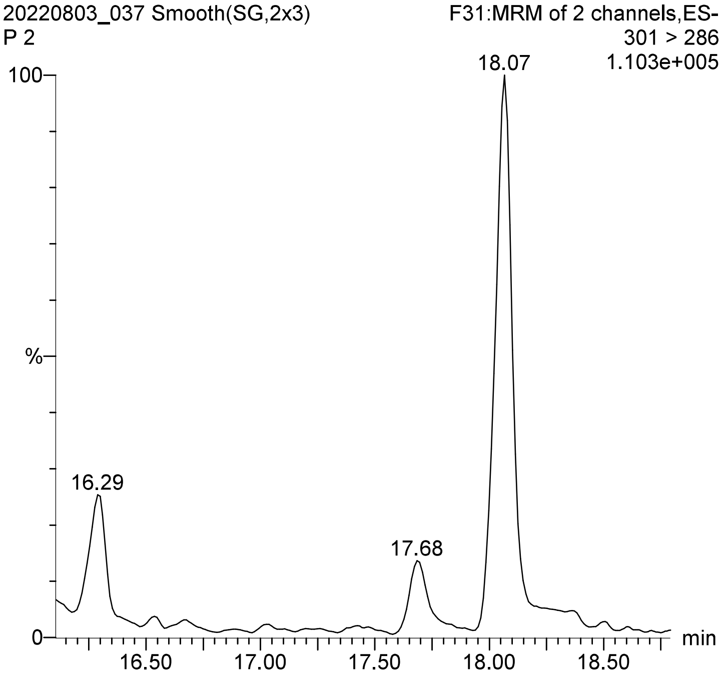

Taxifolin

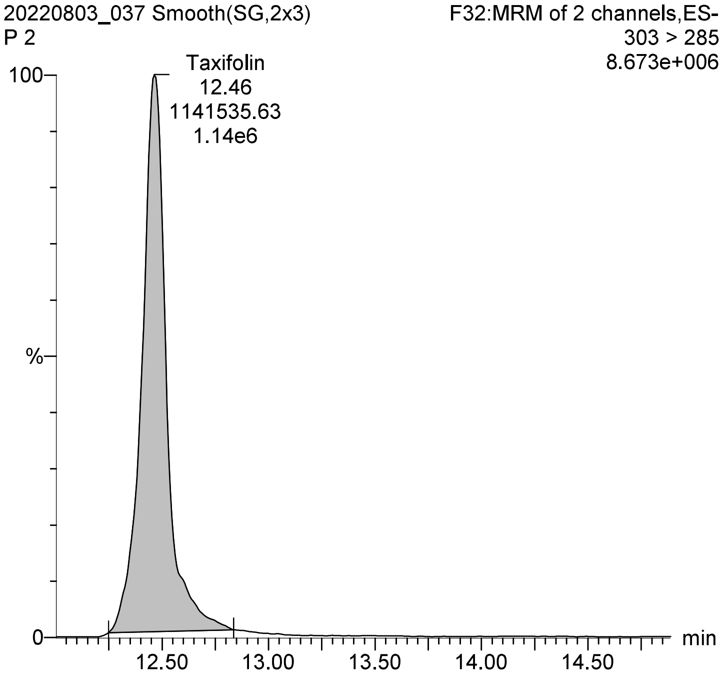

Dataset: W:\QACL\personeel\Stijn\MEET@ALL\Polyfenolen\TargetLynx\Resultaten\20220803 Toufik.qld

Last Altered: Monday, August 08, 2022 08:07:15 Romance (zomertijd)
Printed: Thursday, November 17, 2022 11:26:49 Romance (standaardtijd)

Name: 20220803_037, Date: 04-Aug-2022, Time: 05:26:14, ID: , Description: P 2

Taxifolin

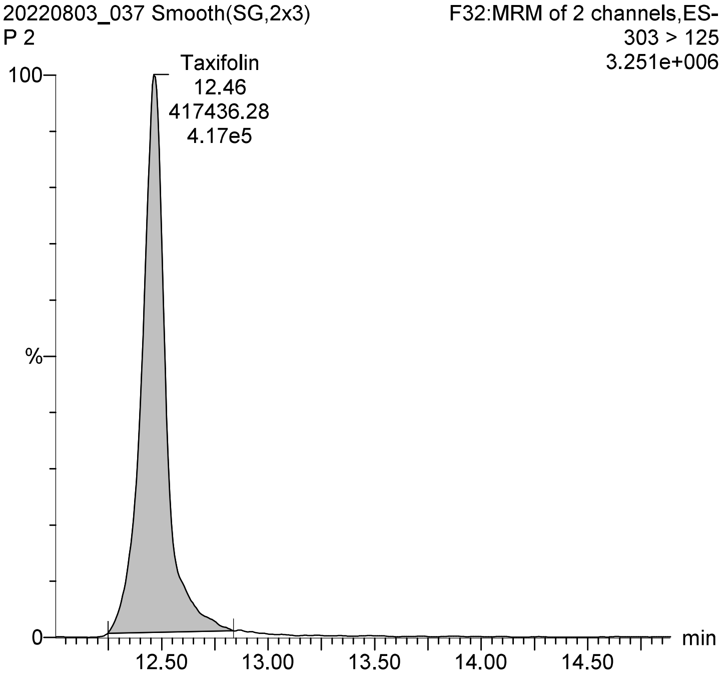

Cyanidin

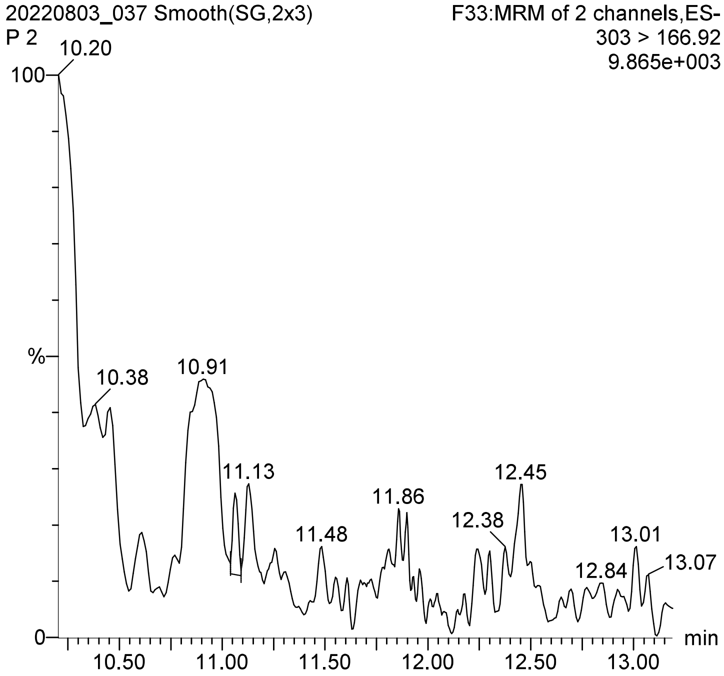

Cyanidin

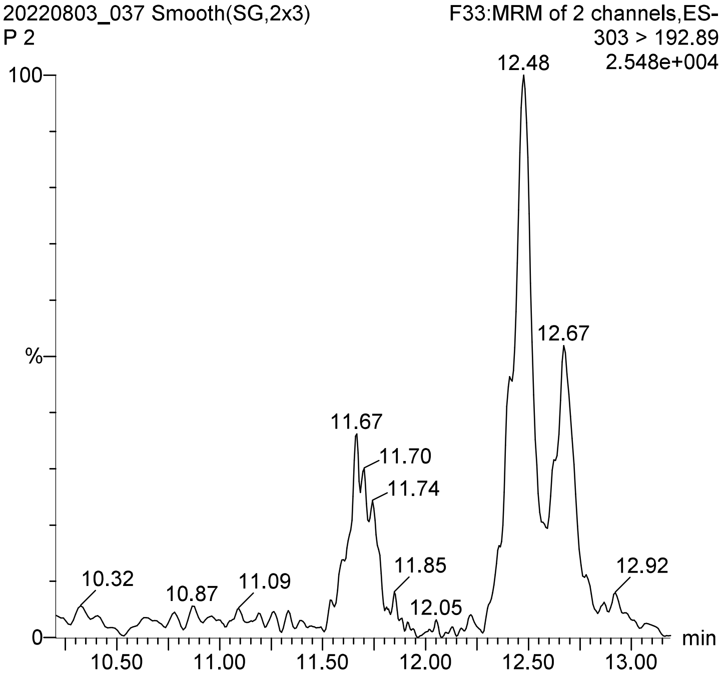

Isorhamnetin

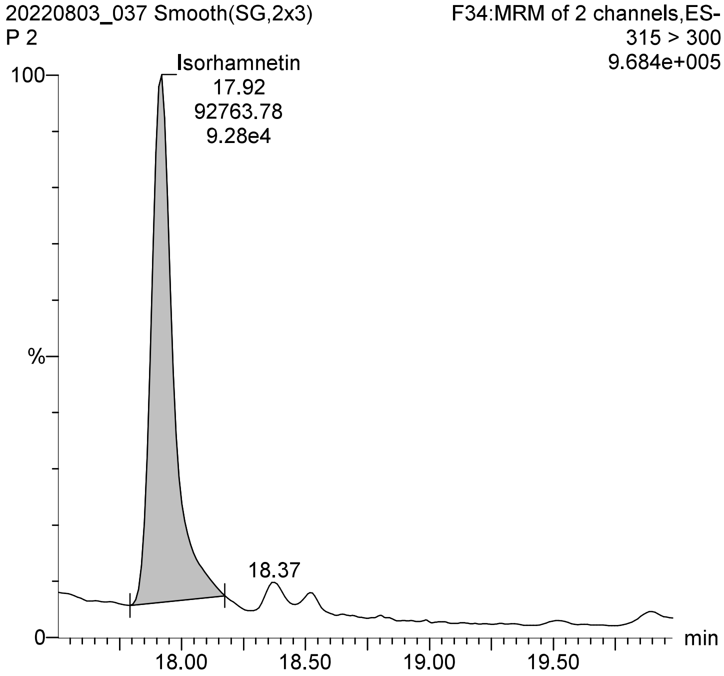

Dataset: W:\QACL\personeel\Stijn\MEET@ALL\Polyfenolen\TargetLynx\Resultaten\20220803 Toufik.qld

Last Altered: Monday, August 08, 2022 08:07:15 Romance (zomertijd)
Printed: Thursday, November 17, 2022 11:26:49 Romance (standaardtijd)

Name: 20220803_037, Date: 04-Aug-2022, Time: 05:26:14, ID: , Description: P 2

Isorhamnetin

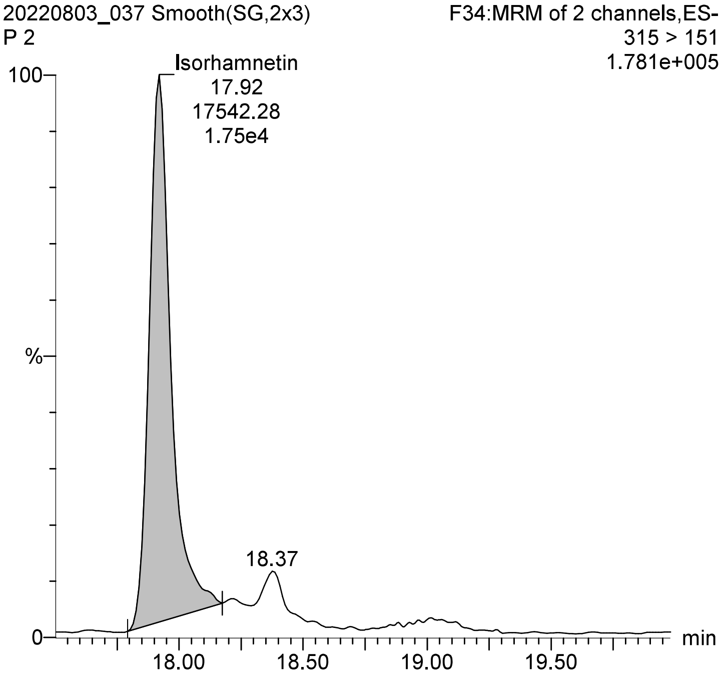

Chlorogenic acid

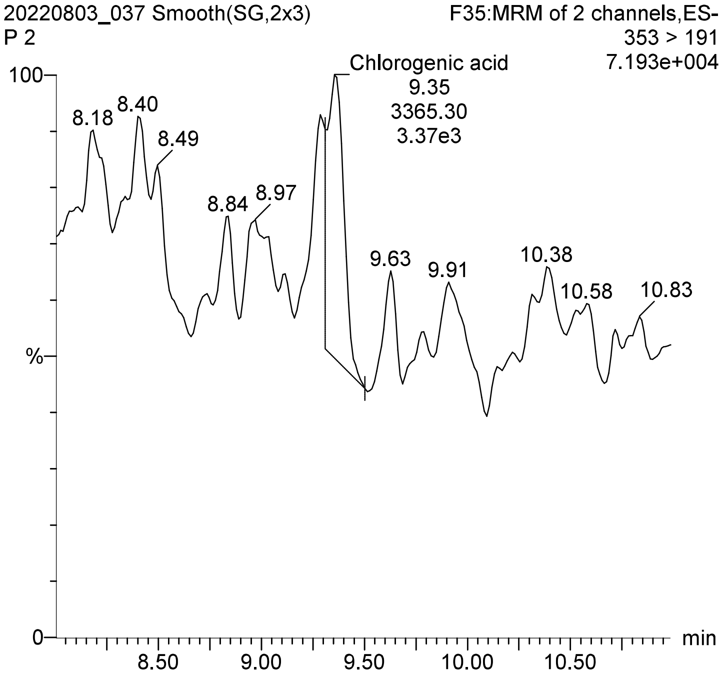

Chlorogenic acid

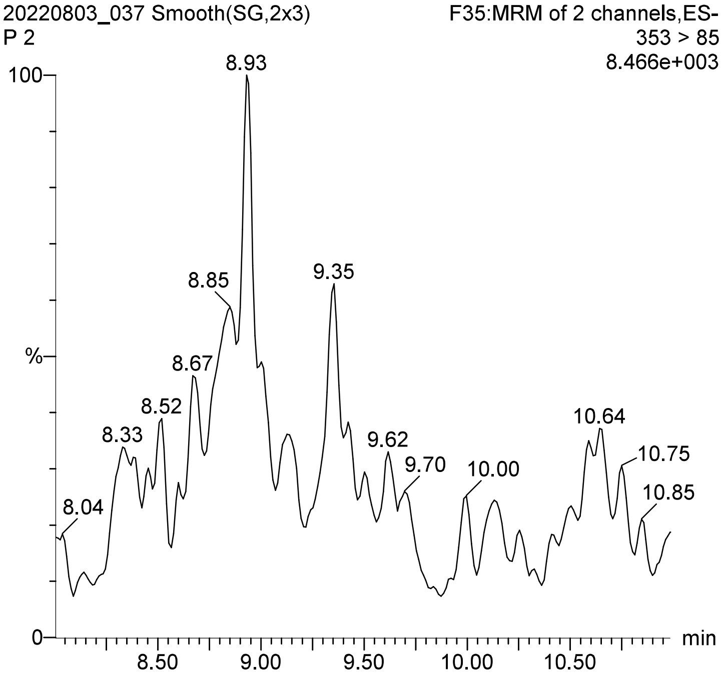

Apigetrin

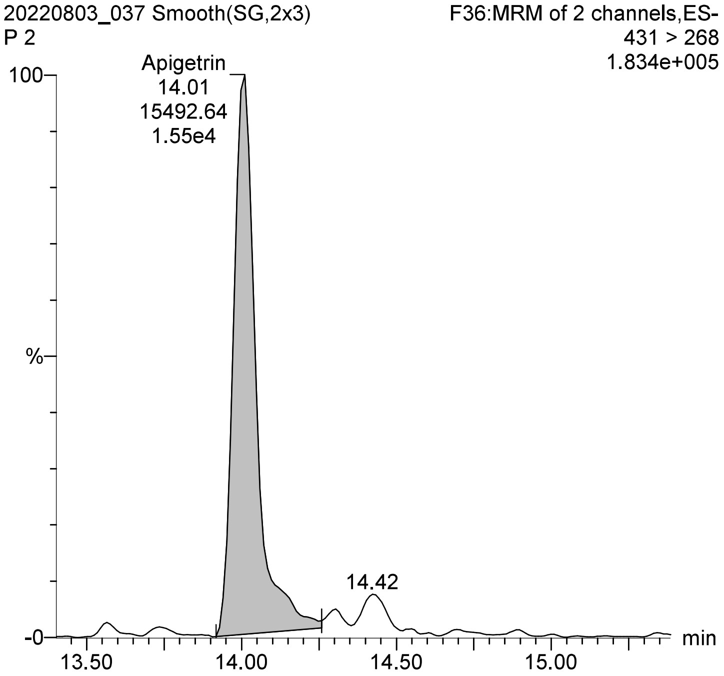

Dataset: W:\QACL\personeel\Stijn\MEET@ALL\Polyfenolen\TargetLynx\Resultaten\20220803 Toufik.qld

Last Altered: Monday, August 08, 2022 08:07:15 Romance (zomertijd)
Printed: Thursday, November 17, 2022 11:26:49 Romance (standaardtijd)

Name: 20220803_037, Date: 04-Aug-2022, Time: 05:26:14, ID: , Description: P 2

Apigetrin

20220803_037 Smooth(SG,2x3) F36:MRM of 2 channels,ES-
P 2 431 > 107
1.161e+004

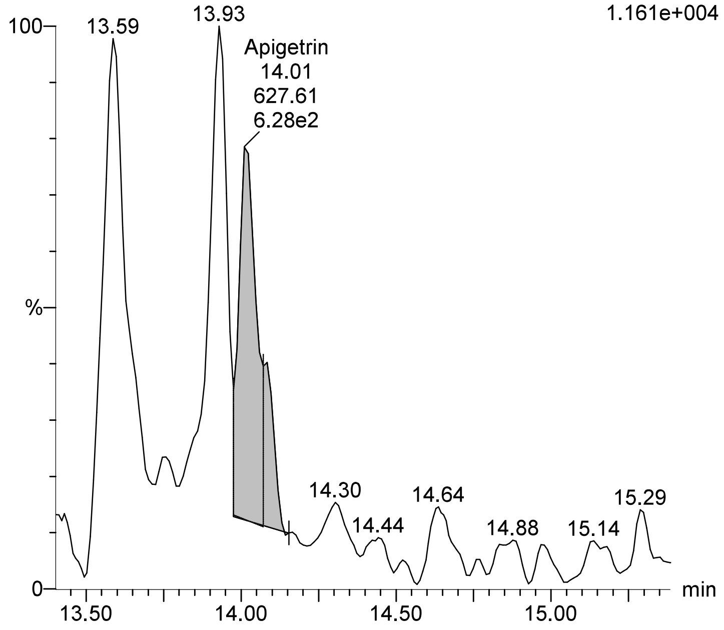

Avicularin

20220803_037 Smooth(SG,2x3) F37:MRM of 2 channels,ES-
P 2 433 > 300
1.968e+007

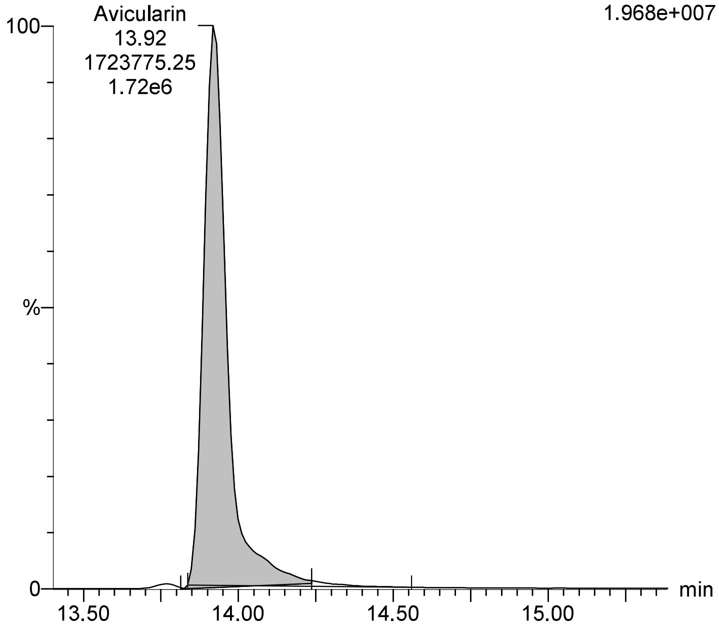

Avicularin

20220803_037 Smooth(SG,2x3) F37:MRM of 2 channels,ES-
P 2 433 > 271
1.230e+007

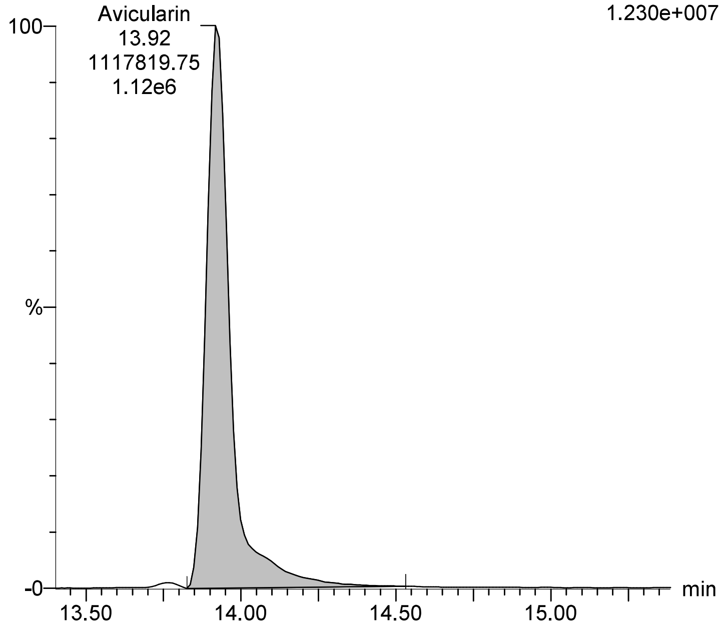

Phloridzin

20220803_037 Smooth(SG,2x3) F38:MRM of 2 channels,ES-
P 2 435 > 273
8.449e+006

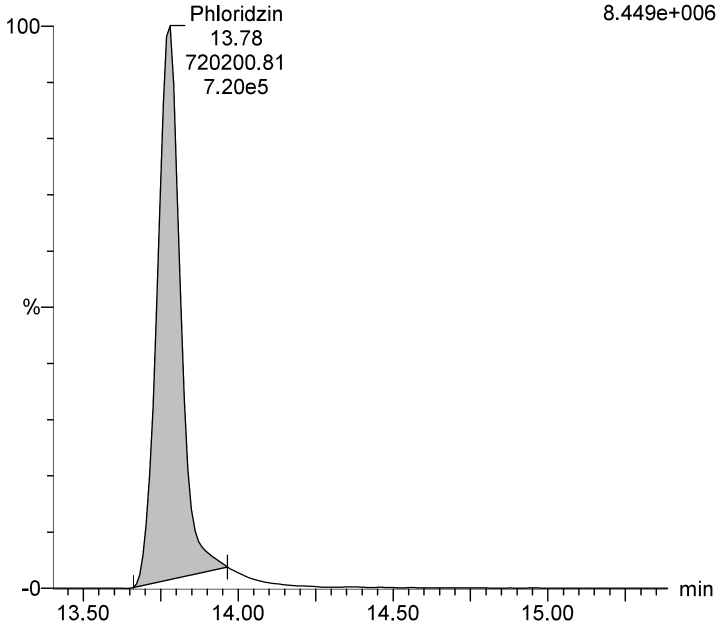

Name: 20220803_037, Date: 04-Aug-2022, Time: 05:26:14, ID: , Description: P 2

Phloridzin

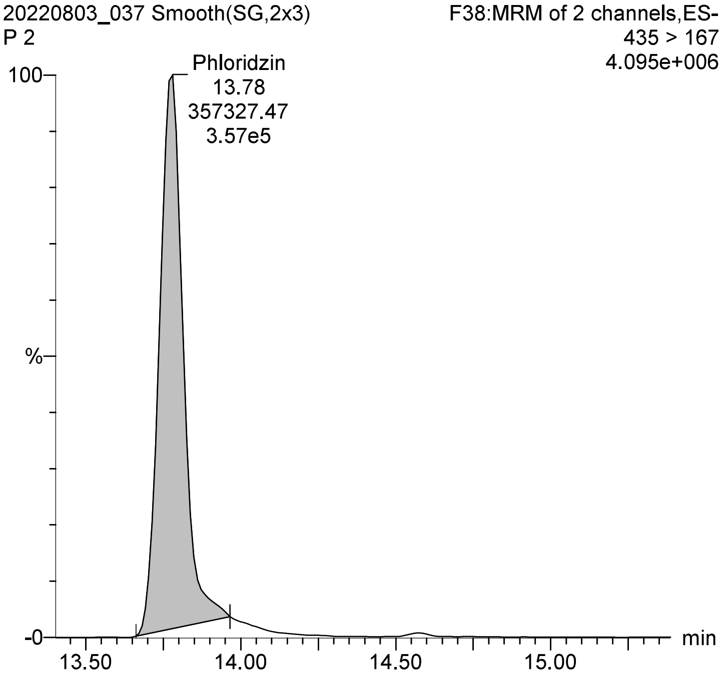

Cynaroside

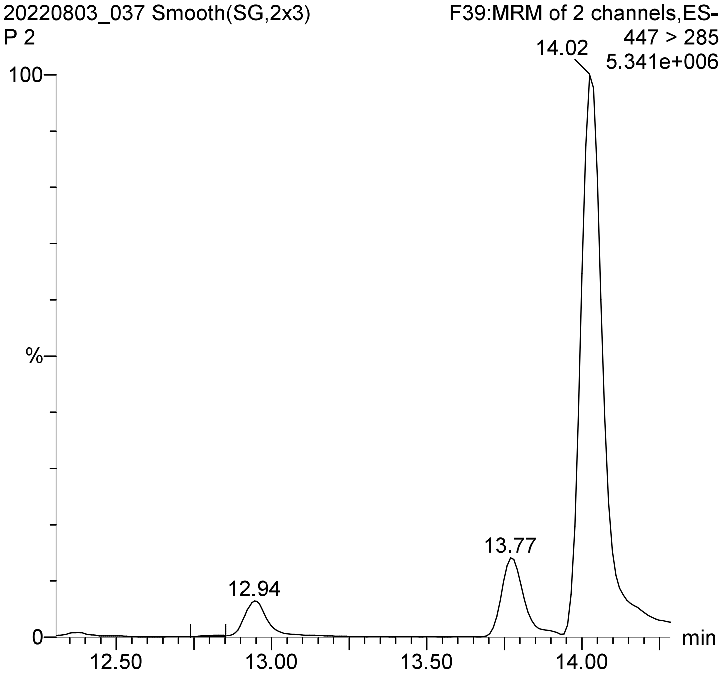

Cynaroside

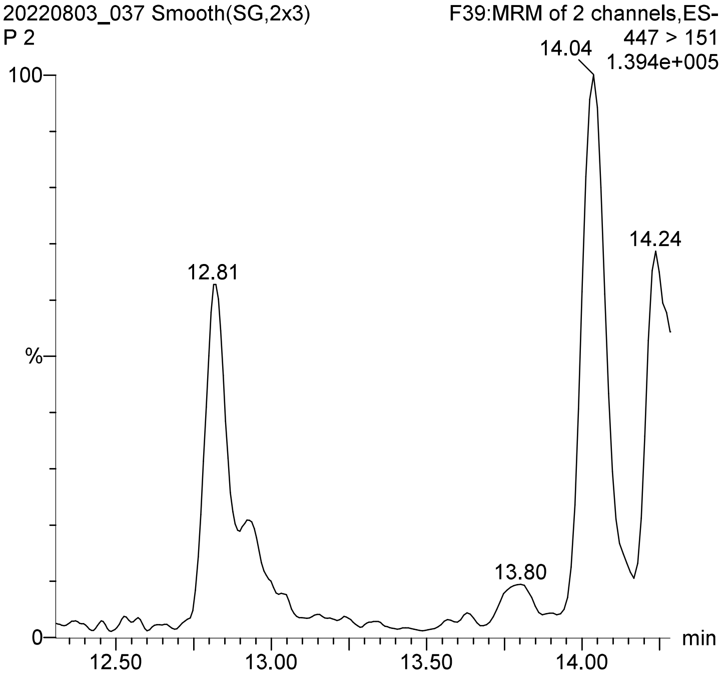

Astragalin

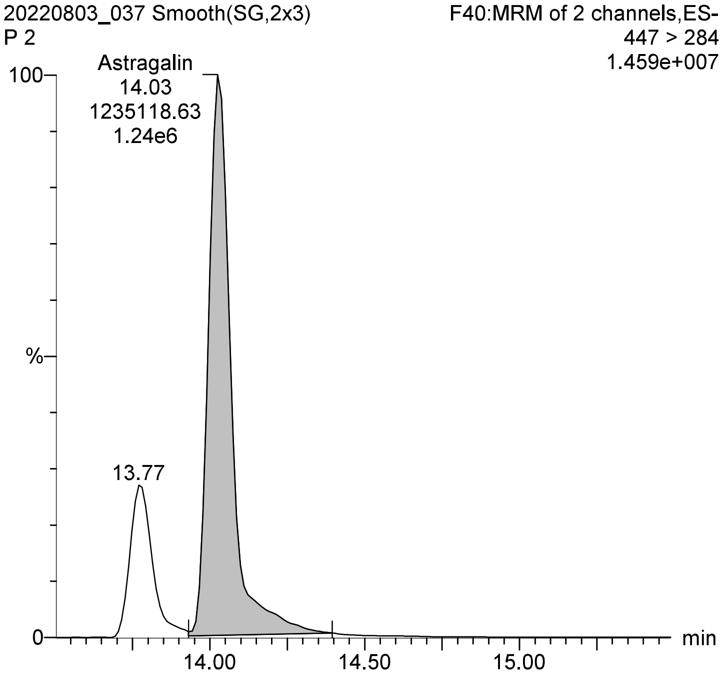

Dataset: W:\QACL\personeel\Stijn\MEET@ALL\Polyfenolen\TargetLynx\Resultaten\20220803 Toufik.qld

Last Altered: Monday, August 08, 2022 08:07:15 Romance (zomertijd)
Printed: Thursday, November 17, 2022 11:26:49 Romance (standaardtijd)

Name: 20220803_037, Date: 04-Aug-2022, Time: 05:26:14, ID: , Description: P 2

Astragalin

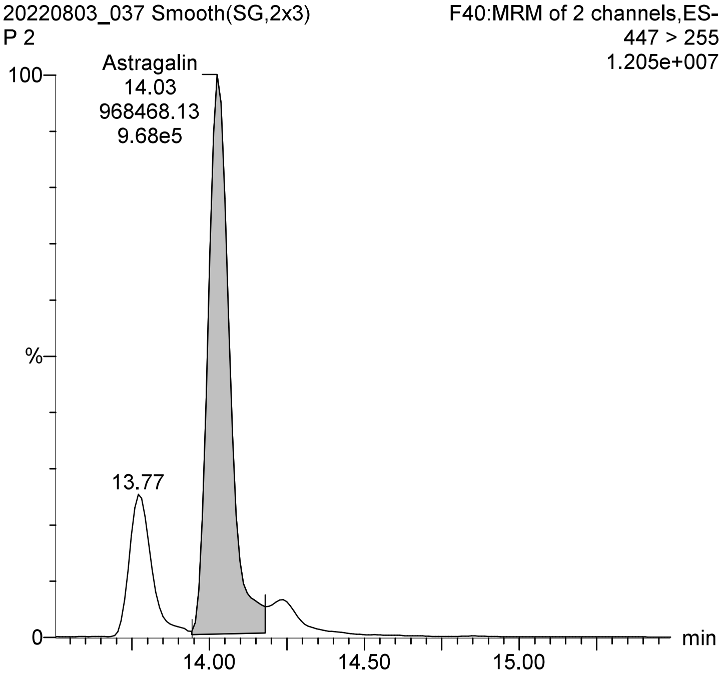

Isoquercetin

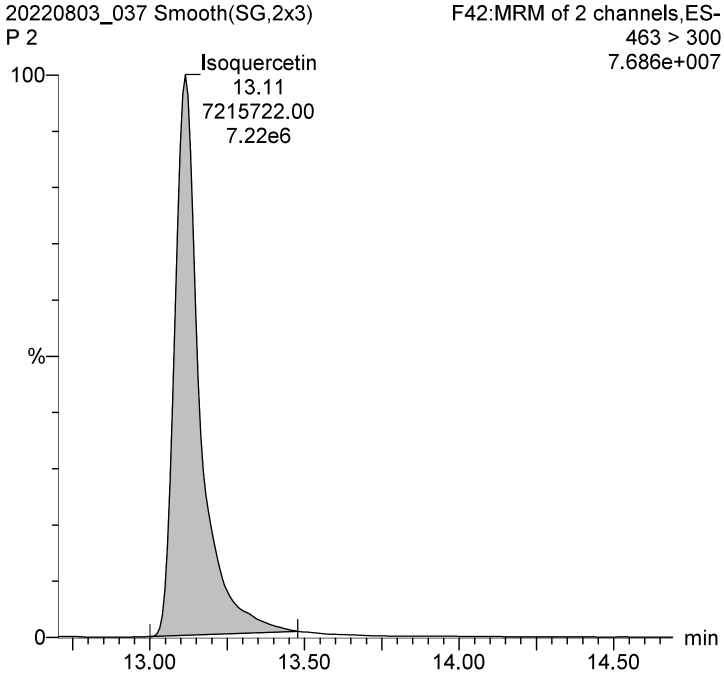

Isoquercetin

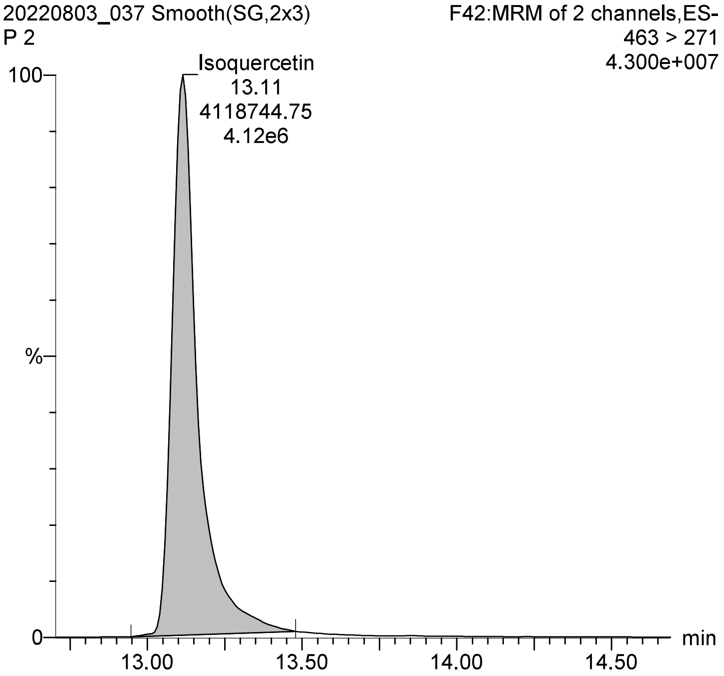

Quercetrin

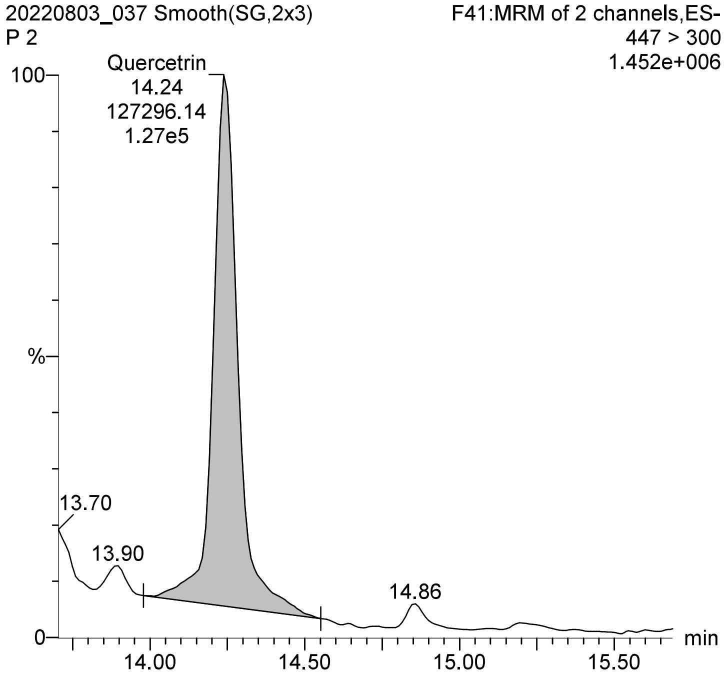

Dataset: W:\QACL\personeel\Stijn\MEET@ALL\Polyfenolen\TargetLynx\Resultaten\20220803 Toufik.qld

Last Altered: Monday, August 08, 2022 08:07:15 Romance (zomertijd)
Printed: Thursday, November 17, 2022 11:26:49 Romance (standaardtijd)

Name: 20220803_037, Date: 04-Aug-2022, Time: 05:26:14, ID: , Description: P 2

Quercetrin

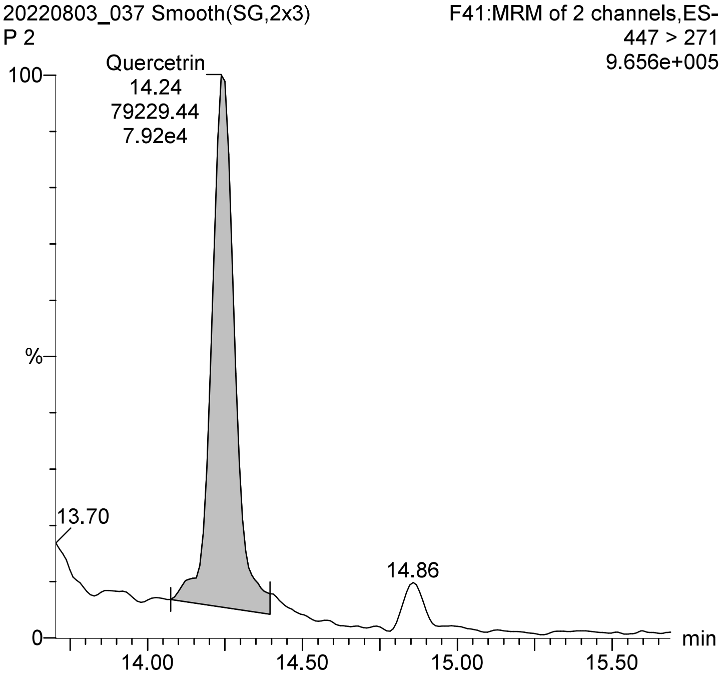

Chicoric acid

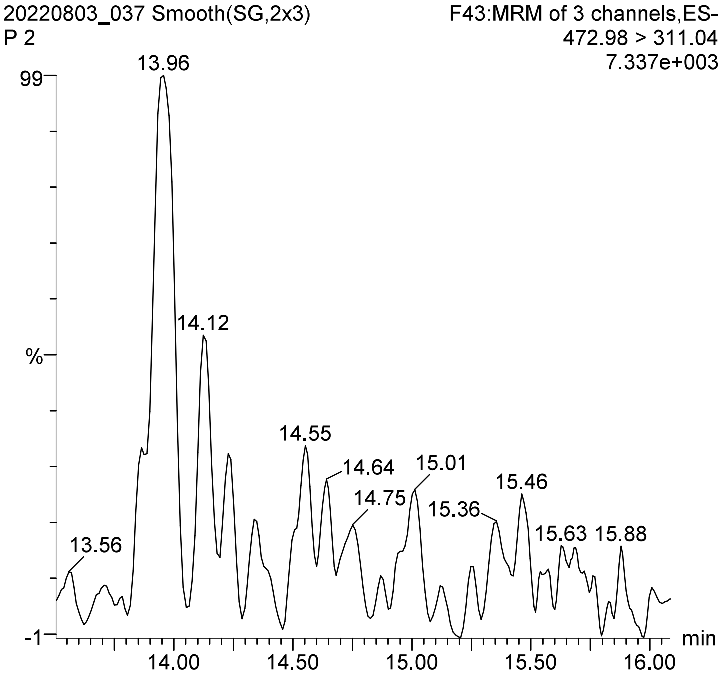

Chicoric acid

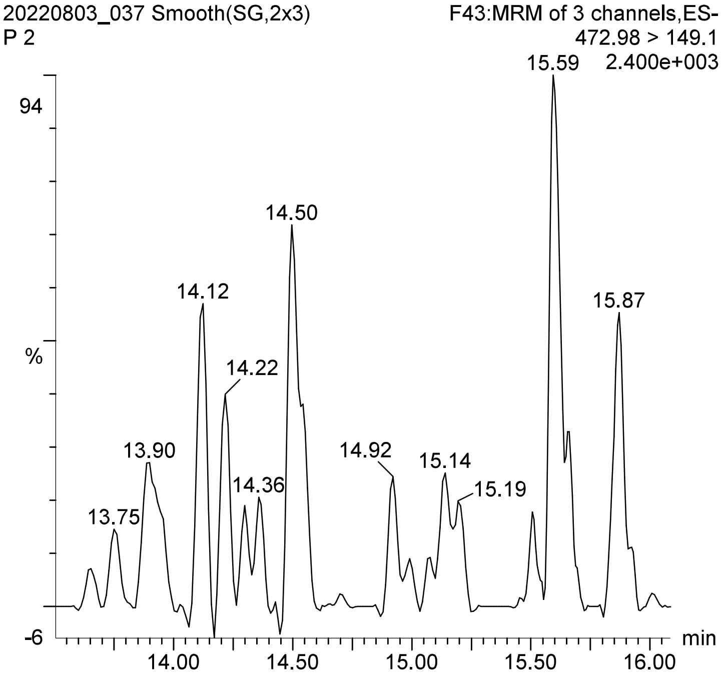

Quercetin-3-O-glucuronide

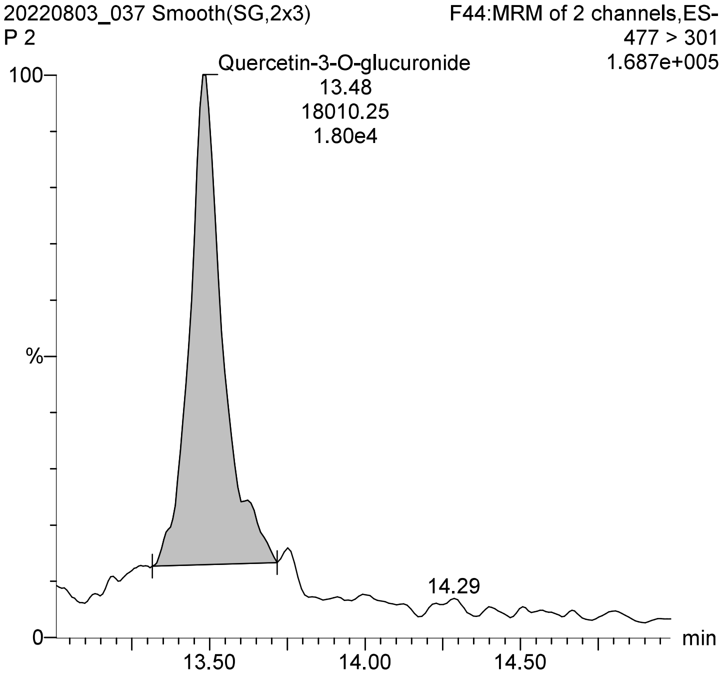

Dataset: W:\QACL\personeel\Stijn\MEET@ALL\Polyfenolen\TargetLynx\Resultaten\20220803 Toufik.qld

Last Altered: Monday, August 08, 2022 08:07:15 Romance (zomertijd)
Printed: Thursday, November 17, 2022 11:26:49 Romance (standaardtijd)

Name: 20220803_037, Date: 04-Aug-2022, Time: 05:26:14, ID: , Description: P 2

Quercetin-3-O-glucuronide

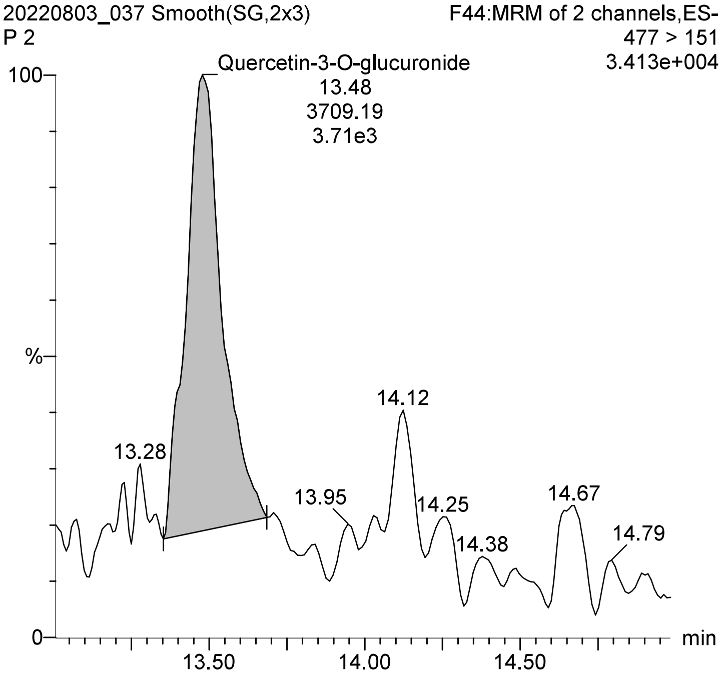

Procyanidin B2

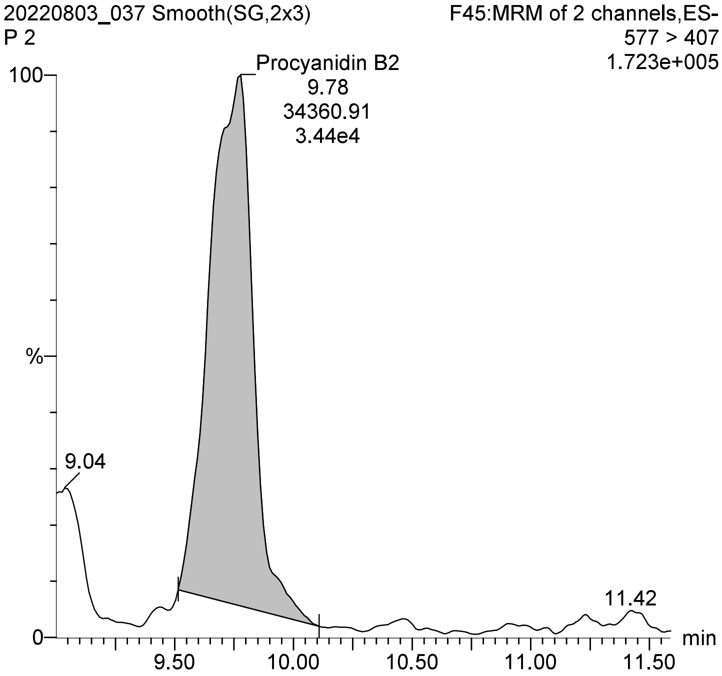

Procyanidin B2

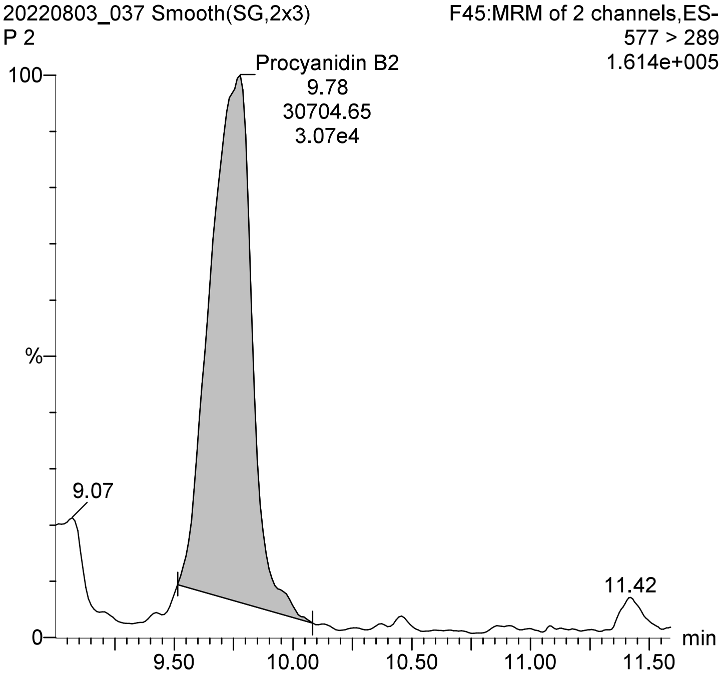

Naringin

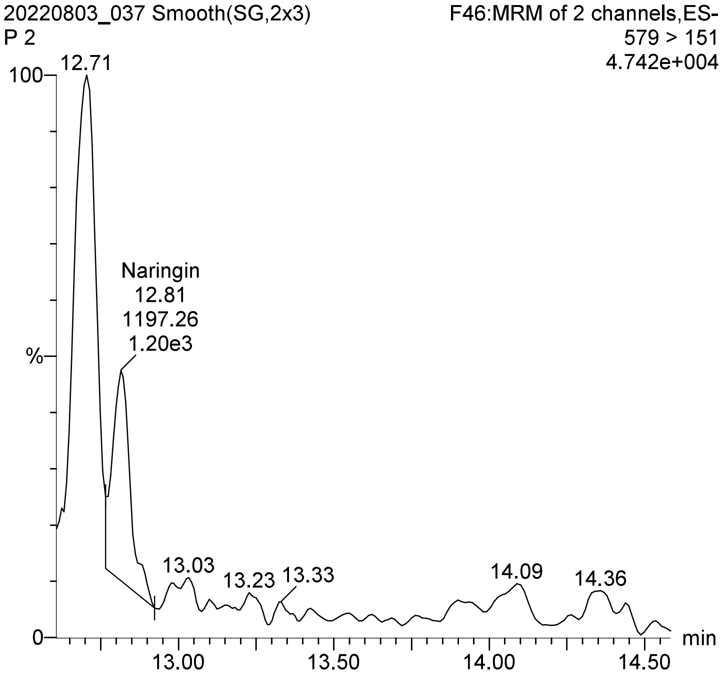

Dataset: W:\QACL\personeel\Stijn\MEET@ALL\Polyfenolen\TargetLynx\Resultaten\20220803 Toufik.qld

Last Altered: Monday, August 08, 2022 08:07:15 Romance (zomertijd)
Printed: Thursday, November 17, 2022 11:26:49 Romance (standaardtijd)

Name: 20220803_037, Date: 04-Aug-2022, Time: 05:26:14, ID: , Description: P 2

Naringin

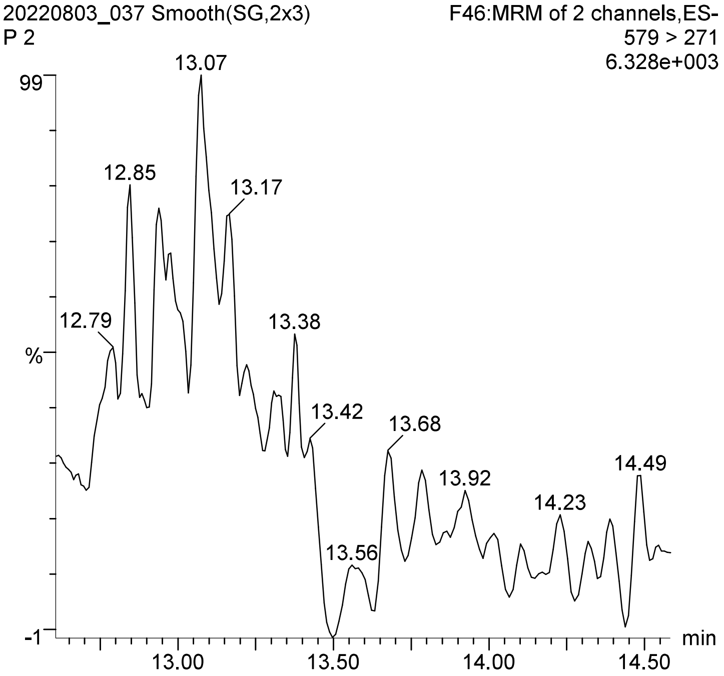

Hesperidin

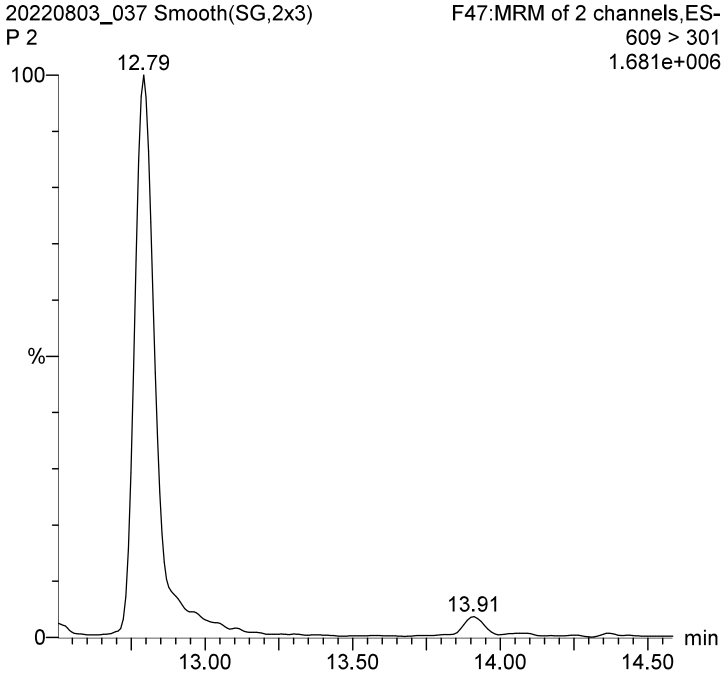

Hesperidin

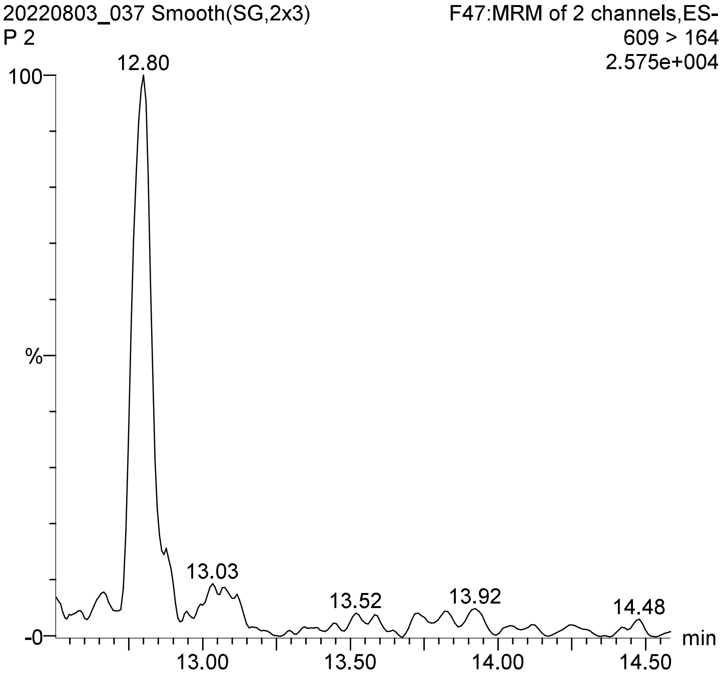

Rutin

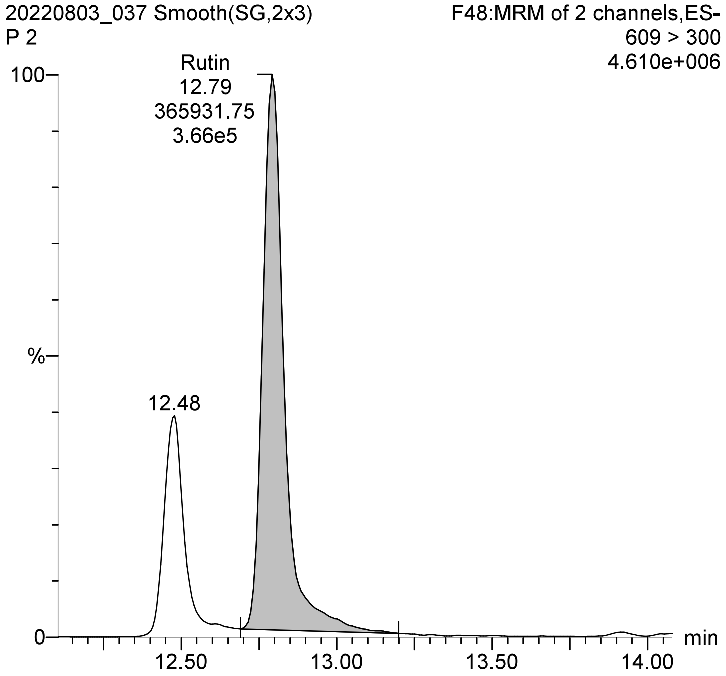

Dataset: W:\QACL\personeel\Stijn\MEET@ALL\Polyfenolen\TargetLynx\Resultaten\20220803 Toufik.qld

Last Altered: Monday, August 08, 2022 08:07:15 Romance (zomertijd)
Printed: Thursday, November 17, 2022 11:26:49 Romance (standaardtijd)

Name: 20220803_037, Date: 04-Aug-2022, Time: 05:26:14, ID: , Description: P 2

Rutin

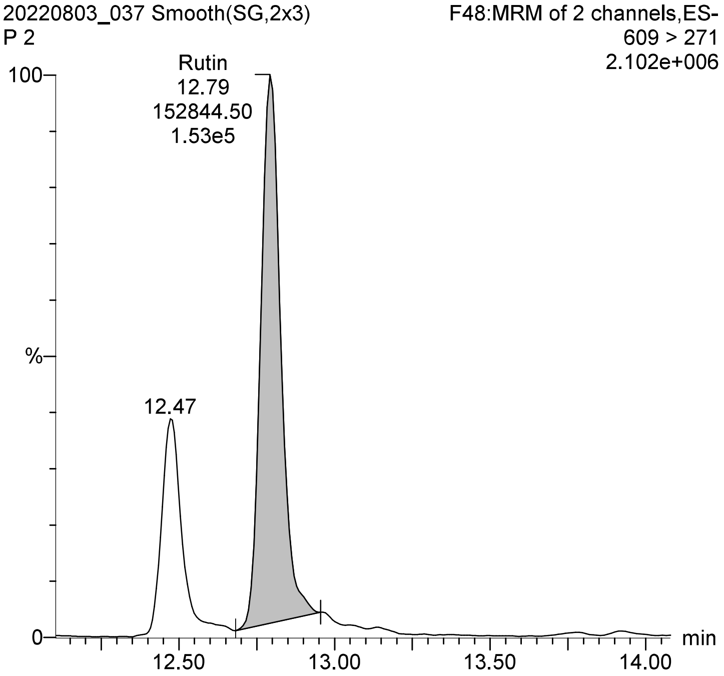

Supplement: Supplementary file 1 — Supplementary Information 1. [file 41598_2023_50031_MOESM1_ESM.pdf]
