# Supplementary material for: Ionomic analysis, polyphenols characterization, analgesic, antiinflammatory and antioxidant capacities of Cistus laurifolius leaves: in vitro, in vivo, and in silico investigations
Source: Sci Rep. 2023 Dec 21;13:22890. doi: 10.1038/s41598-023-50031-5 (PMC10739726; doi:10.1038/s41598-023-50031-5)
Supplement: Supplementary file 2 — Supplementary Tables. [file 41598_2023_50031_MOESM2_ESM.docx]

Ionomic Analysis, Polyphenols Characterization, Analgesic, Antiinflammatory and Antioxidant Capacities of *Cistus laurifolius* Leaves: *in vitro*, *in vivo*, and in silico Investigations

**Supplementary material**

**Table S1**: The retention times and optimized mass spectrometry parameters were determined for the phenolic compounds detected using Multiple Reaction Monitoring (MRM) transitions.

| Compound Number | Analyte | Retention time (min) | Precursor  Ion (m/z) | Product Ions (m/z) | Ionization Mode (+/-) |
| --- | --- | --- | --- | --- | --- |
| I | Gallic acid | 4.40 | 169  169 | 125  79 | ES- |
| II | Protocatechuic acid | 6.28 | 153  153 | 109 | ES- |
| III | Isoquercetin | 13.11 | 463  463 | 300  271 | ES- |
| IV | 4-OH-phenylacetic acid | 7.34 | 151  137 | 107 | ES- |
| V | P- Coumaric acid | 11.51 | 163  163 | 119 | ES- |
| VI | Vanillic acid | 8.94 | 167  167 | 152 | ES- |
| VII | Avicularin | 13.92 | 433  433 | 300  271 | ES- |
| VIII | Quinic acid | 2.17 | 191  191 | 85  93 | ES- |
| IX | Taxifolin | 12.46 | 303  303 | 285  125 | ES- |
| X | Astragalin | 14.03 | 447  447 | 284  255 | ES- |
| XI | Aromadendrin | 14.15 | 287  287 | 259  125 | ES- |
| XII | Ferulic acid | 11.95 | 193  193 | 134  178 | ES- |
| XIII | Rutin | 12.79 | 609  609 | 300  271 | ES- |
| XIV | Quercetin | 16.53 | 301  301 | 151  179 | ES- |
| XV | Phloridzin | 13.78 | 435  435 | 273  167 | ES- |
| XVI | Gentisic acid | 8.10 | 153  153 | 109 | ES- |
| XVII | Epicatechin | 9.82 | 289  289 | 245  109 | ES- |
| XVIII | Catechin | 8.85 | 289  289 | 245  109 | ES- |
| XIX | Procyanidin B2 | 9.78 | 577  577 | 407  289 | ES- |
| XX | Quercetrin | 14.24 | 447  447 | 300  271 | ES- |
| XXI | Hydroferulic acid | 10.11 | 195  195 | 121  93 | ES- |
| XXII | Kaempferol | 17.99 | 285  285 | 93  146 | ES- |
| XXIII | Caffeic acid | 9.59 | 179  179 | 135  107 | ES- |
| XXIV | Naringenin | 16.74 | 271  271 | 151  119 | ES- |
| XXV | Salicylic acid | 12.43 | 137  137 | 93 | ES- |
| XXVI | Apigenin | 17.74 | 269  269 | 117  149 | ES- |
| XXVII | Luteolin | 16.48 | 285  285 | 133  107 | ES- |
| XXVIII | 3,4,5-Trimethoxycinnamic acid | 14.69 | 237  237 | 102  132 | ES- |
| XXIX | Isorhamnetin | 17.92 | 315  315 | 300  151 | ES- |
| XXX | Quercetin-3-O-glucuronide | 13.48 | 477  477 | 301  151 | ES- |
| XXXI | Sinapinic acid | 12.01 | 223  223 | 208  164 | ES- |
| XXXII | Resveratrol | 15.49 | 227  227 | 142  158 | ES- |
| XXXIII | Apigetrin | 14.01 | 431  431 | 268  107 | ES- |
| XXXIV | Phloretin | 16.85 | 273  273 | 167  123 | ES- |

ES-: Negative Electrospray

**Table S2** Interactive interaction of molecular docking with all the ligands and target protein 1R4U.

|  |  | **1R4U** |  |
| --- | --- | --- | --- |
| **Ligand**  **Code** | **Chemical Name** | **Binding Score** | **Interactions** |
| 1 | **Gallic acid** | -6.3 | GLU31(2.27)  THR74(2.81)  GLU31(2.14)  GLU31(2.78)  HIS104(2.46)  CYS103(3.82)  MET32(5.66)  TYR30 (4.89)  PRO76(5.37) |
| 2 | **Protocatechuic acid** | -6.2 | GLU31(2.64)  PRO76(3.62)  CYS103(3.72)  MET32(5.40)  TYR30 (4.92)  PRO76(5.26) |
| 3 | **Isoquercetin** | -8.8 | THR74(2.40)  ARG128(2.97)  ASP205(2.21)  PRO76(3.79)  PRO76(3.16)  CYS103(3.67)  MET32(5.26)  TYR30 (5.58)  TYR30 (4.99)  CYS103(4.33)  PRO76(5.46)  ARG105(4.28) |
| 4 | **4-OH-phenylacetic acid** | -5.3 | TRP106(2.14)  VAL73(2.39)  CYS103(3.93)  MET32(5.77)  TYR30 (4.87) |
| 5 | **P- Coumaric acid** | -5.8 | TRP106(1.89)  VAL73(1.78)  CYS103(3.67)  MET32(5.26)  TYR30 (4.95)  PRO76(5.31) |
| 6 | **Vanillic acid** | -5.9 | GLU31(2.29)  ARG128(2.84)  GLU31(2.25)  PRO76(3.51)  VAL29(3.69)  CYS103(3.76)  MET32(5.51)  TYR30 (4.89)  TYR30 (5.19)  PRO76(5.18) |
| 7 | **Avicularin** | -8.6 | THR74(2.37)  ARG128(2.97)  ASP205(2.75)  CYS103(3.71)  MET32(5.38)  TYR30 (5.57)  TYR30 (4.95)  CYS103(4.46)  PRO76(5.42)  ARG105(4.34) |
| 8 | **Quinic acid (AP)** | -5.9 | GLU31(2.70)  HIS104(1.70)  TRP106(2.87)  HIS104(3.14) |
| 9 | **Taxifolin** | -8.7 | THR74(2.37)  THR107(2.48)  PRO76(3.69)  CYS103(3.61)  MET32(5.32)  TYR30 (5.05)  PRO76(5.38)  ARG105(4.41) |
| 10 | **Astragalin** | -8.5 | THR74(3.06)  CYS103(3.01)  ASP205(3.99)  CYS103(3.88)  MET32(5.60)  TYR30 (4.92)  TYR30 (4.81)  CYS103(5.20)  PRO76(5.48) |
| 11 | **Aromadendrin** | -8.3 | THR74(2.49)  VAL73(2.65)  PRO76(3.33)  CYS103(3.65)  MET32(5.33)  TYR30 (5.02)  PRO76(5.44)  ARG105(4.29) |
| 12 | **Ferulic acid** | -5.6 | GLU31(2.26)  GLU31(2.42)  THR74(2.85)  HIS104(2.10)  TYR30 (4.95)  CYS103(3.79)  TYR30 (5.14) |
| 13 | **Rutin** | -8.8 | THR74(2.33)  ARG105(2.78)  ARG128(2.94)  PRO76(3.52)  CYS103(3.61)  MET32(5.26)  TYR30(5.63)  TYR30 (5.04)  CYS103(4.30)  PRO76(5.39)  ARG105(4.47) |
| 14 | **Quercetin** | -8.5 | ARG128(2.50)  VAL73(1.94)  THR107(2.29)  ASP205(4.13)  CYS103(3.97)  MET32(5.76)  TYR30 (5.04)  TYR30 (4.77)  CYS103(5.25) |
| 15 | **Phloridzin** | -7.3 | GLU31(2.10)  TRP106(2.52)  ARG128(2.32)  GLU31(2.64)  VAL29(3.00)  ARG105(3.83)  ARG128(5.32) |
| 16 | **Gentisic acid** | -6.0 | GLU31(2.94)  HIS104(1.79)  PRO76(3.57)  CYS103(3.75)  MET32(5.45)  TYR30(4.90)  PRO76(4.93) |
| 17 | **Epicatechin** | -8.4 | THR107(2.44)  PRO76(3.20)  CYS103(3.68)  MET32(5.42)  TYR30 (5.00)  PRO76(5.44)  ARG105(4.56) |
| 18 | **Catechin** | -8.2 | HIS104(2.32)  VAL73(2.33)  CYS103(3.95)  MET32(5.89)  TYR30 (4.91) |
| 19 | **Procyanidin B2** | -9.4 | GLU31(2.21)  ASP205(4.12)  CYS103(3.87)  MET32(5.85)  TYR30 (4.92)  PRO76(5.46) |
| 20 | **Quercetrin** | -9.3 | THR74(2.49)  ARG128(2.87)  PRO76(3.67)  PRO76(3.35)  CYS103(3.59)  MET32(5.29)  TYR30 (5.07)  CYS103(4.14)  PRO76(5.41)  ARG105(4.18) |
| 21 | **Hydroferulic acid** | -5.4 | ARG105(2.14)  ARG128(2.70)  TRP106(3.20)  ARG105(3.99)  CYS103(3.87)  TYR30 (5.37) |
| 22 | **Kaempferol** | -8.5 | THR74(2.44)  THR74(2.54)  THR107(2.31)  VAL73(2.45)  CYS103(3.69)  MET32(5.41)  TYR30 (4.99)  CYS103(4.43)  PRO76(5.48)  ARG105(4.19) |
| 23 | **Caffeic acid** | -6.5 | GLU31(2.27)  ARG128(2.60)  GLU31(2.24)  HIS104(2.91)  ASP205(2.63)  ASP205(2.60)  CYS103(3.64)  MET32(5.30)  TYR30 (4.98)  PRO76(5.20) |
| 24 | **Naringenin** | -8.4 | THR74(2.47)  CYS103(3.65)  MET32(5.38)  TYR30 (5.03)  PRO76(5.45)  ARG105(4.30) |
| 25 | **Salicylic acid** | -5.7 | CYS103(3.67)  MET32(5.16)  TYR30 (4.94)  PRO76(5.35) |
| 26 | **Apigenin** | -8.6 | THR107(2.94)  VAL73(2.56)  GLU31(2.28)  THR28(3.87)  ARG105(4.82) |
| 27 | **Luteolin** | -8.9 | THR74(2.52)  TRP106(2.39)  VAL73(2.81)  PRO76(3.23)  CYS103(3.67)  MET32(5.52)  TYR30 (5.04)  CYS103(4.39)  PRO76(5.41)  ARG105(4.38) |
| 28 | **3,4,5 trimethoxycinnamic acid** | -5.9 | THR74(3.04)  TRP106(2.11)  VAL73(2.00)  PRO76(3.28)  ARG105(3.38)  CYS103(3.55)  ARG105(4.29)  CYS103(4.61)  ARG105(4.05)  ARG128(4.48)  TYR30 (4.85) |
| 29 | **Isorhamnetin** | -8.4 | THR74(2.46)  THR74(2.50)  THR107(3.39)  CYS103(3.71)  MET32(5.47)  TYR30 (5.61)  TYR30 (4.99)  CYS103(4.51)  PRO76(5.48)  ARG105(4.38) |
| 30 | **Quercetin-3-O-glucuronide** | -9.0 | THR74(2.47)  TRP106(2.67)  THR107(2.86)  ARG128(2.86)  ASP205(2.36)  PRO76(3.63)  CYS103(3.60)  MET32(5.28)  TYR30 (5.80)  TYR30 (5.06)  CYS103(4.15)  PRO76(5.39)  ARG105(4.20) |
| 31 | **Sinapinic acid** | -6.1 | GLU31(2.38)  GLU31(1.98)  THR74(2.48)  ASP205(2.16)  TYR30 (4.93)  MET32(3.75)  CYS103(3.80)  TYR30 (5.39)  CYS103(5.00) |
| 32 | **Resveratrol** | -6.7 | THR107(2.26)  ARG105(4.86)  CYS103(3.79)  MET32(5.49)  TYR30 (4.88)  ARG105(4.47)  PRO76(5.38) |
| 33 | **Apigetrin** | -8.7 | GLU31(2.02)  GLU31(2.17)  THR28(3.85)  ARG105(4.81) |
| 34 | **Phloretin** | -7.4 | TRP106(2.26)  VAL73(2.11)  HIS104(2.75)  CYS103(3.72)  TYR30 (5.18)  PRO76(4.95)  ARG105(3.96) |

**Table S3** Interactive interaction of molecular docking with all the ligands and target protein 3LN1.

|  |  | **3LN1** |  |
| --- | --- | --- | --- |
| **Ligand**  **Code** | **Chemical Name** | **Binding Score** | **Interactions** |
| 1 | **Gallic acid** | -6.1 | TYR371(2.53)  MET508(2.63)  VAL335(2.39)  SER516(2.32)  GLY512(3.43)  LEU338(3.81)  VAL335(5.24)  ALA513(4.86) |
| 2 | **Protocatechuic acid** | -6.2 | THR192(2.82)  HIS374 (5.32)  ALA188(5.14) |
| 3 | **Isoquercetin** | -8.1 | HIS193(2.78)  HIS193(2.18)  ASN368(2.27)  HIS374(2.70)  GLN275(2.30)  PHE196(2.73)  THR198(2.99)  PHE196(2.18)  VAL277(3.25)  LYS197(3.50)  THR198(5.20)  VAL277(5.33)  VAL277(4.66) |
| 4 | **4-OH-phenylacetic acid** | -6.3 | HIS374(3.45)  HIS374(3.99)  HIS374 (5.63)  ALA188(4.70) |
| 5 | **P- Coumaric acid** | -6.6 | ALA185(2.18)  HIS374(5.37)  ALA188(4.81)  ALA188(4.96) |
| 6 | **Vanillic acid** | -5.8 | VAL335(2.69)  GLY512(3.29)  LEU338(3.92)  VAL335(4.50)  LEU338(5.05)  TYR334 (5.44)  TYR371 (5.32)  VAL509(5.01) |
| 7 | **Avicularin** | -7.8 | HIS200(2.45)  GLN275(2.33)  HIS374(3.06)  GLN440(2.75)  ASN368(2.92)  HIS193(3.65)  HIS372(4.28)  HIS372(2.92)  VAL277(3.92)  HIS193 (5.39)  HIS372 (4.69)  VAL277(5.23) |
| 8 | **Quinic acid (AP)** | -6.5 | GLN189(2.89)  HIS193(2.98)  TRP373(2.68)  HIS374(2.67)  ALA185(2.22)  HIS374(3.26) |
| 9 | **Taxifolin** | -7.3 | THR198(2.89)  GLN275(1.87)  HIS372(2.34)  HIS372(2.07)  GLN440(2.49)  HIS200(3.50)  HIS200(4.31)  VAL433(4.89)  ALA436(5.38)  LYS197(5.44)  VAL277(5.29) |
| 10 | **Astragalin** | -7.6 | GLN336(2.01)  LYS344(2.98)  PHE566(1.85)  TYR341(2.32)  GLY340(2.20)  HIS337(3.75)  LYS344(4.82) |
| 11 | **Aromadendrin** | -6.8 | THR198(2.47)  THR198(2.41)  HIS372(2.93)  GLN440(2.51)  HIS200(3.44)  HIS200(4.12)  VAL433(5.17)  LYS197(5.38)  VAL277(4.84) |
| 12 | **Ferulic acid** | -6.6 | TYR371(2.66)  SER339(3.31)  VAL335(5.18)  TYR341(4.56)  LEU338(4.89)  VAL509(4.18)  ALA513(5.01) |
| 13 | **Rutin** | -9.2 | HIS372(2.72)  HIS372(2.23)  HIS374(1.79)  GLN440(2.47)  GLN440(2.32)  ASN368(2.46)  VAL433(3.96)  VAL433(3.77)  HIS372(3.80)  VAL433(5.20)  VAL433(4.84)  ALA436(5.07) |
| 14 | **Quercetin** | -8.2 | HIS337(2.43)  PHE566(2.18)  ASN567(3.05)  ASP333(1.83)  GLN336(2.09) |
| 15 | **Phloridzin** | -6.8 | GLN189(1.98)  HIS200(2.50)  HIS372(2.86)  ASN368(2.41)  VAL433(2.02)  VAL433(5.13)  VAL433(4.52)  ALA436(4.18) |
| 16 | **Gentisic acid** | -6.6 | THR192(2.73)  TRP373(2.52)  TYR371(2.77)  HIS374 (4.99)  TRP373(3.81)  ALA188(5.43)  LEU376(5.46) |
| 17 | **Epicatechin** | -7.4 | GLN178(2.36)  GLY340(3.62) |
| 18 | **Catechin** | -6.8 | TYR341(2.77)  HIS337(3.61) |
| 19 | **Procyanidin B2** | -8.9 | THR198(2.61)  HIS374(2.37)  GLU276(2.52)  HIS200(3.60)  HIS193(3.82)  HIS193 (4.73)  HIS372 (5.38)  VAL433(4.95)  VAL430(5.17)  VAL277(3.86) |
| 20 | **Quercetrin** | -8.2 | HIS337(2.41)  PHE566(2.28)  TYR341(2.26) |
| 21 | **Hydroferulic acid** | -5.3 | GLU384(2.93)  GLN415(2.50)  ILE416(2.19)  ASN382(2.61)  GLU384(1.97)  ARG414(5.14) |
| 22 | **Kaempferol** | -8.7 | HIS75(2.17)  LEU338(3.70)  SER339(3.59)  VAL509(3.85)  VAL509(3.32)  LEU338(4.94)  ALA513(5.35)  VAL335(4.95) |
| 23 | **Caffeic acid** | -6.8 | LEU338(2.09)  GLY512(4.14)  LEU338(5.25)  VAL509(5.43) |
| 24 | **Naringenin** | -7.5 | HIS193(2.61)  THR198(2.64)  LYS197(3.51)  GLN275(3.21)  HIS193 (5.10)  LYS197(4.52)  VAL277(4.94) |
| 25 | **Salicylic acid** | -6.3 | TYR371(2.61)  HIS374 (5.00)  TRP373(3.82)  ALA188(5.43)  LEU376(5.45) |
| 26 | **Apigenin** | -8.4 | THR198(2.30)  ASN368(3.05)  THR198(2.21)  HIS193(4.36)  HIS193(3.51)  HIS374(3.26)  HIS372 (5.65)  VAL433(5.10) |
| 27 | **Luteolin** | -7.7 | GLN336(2.34)  HIS337(2.51)  ASP333(2.42)  HIS337(3.71) |
| 28 | **3,4,5 trimethoxycinnamic acid** | -5.9 | HIS200(2.00)  HIS200(3.55)  PHE196(3.53)  GLU276(4.82)  VAL277(4.65)  VAL277(4.83)  HIS193 (4.28)  HIS200 (5.20)  HIS200 (4.87)  LYS197(5.23)  VAL277(5.17) |
| 29 | **Isorhamnetin** | -7.2 | ARG208(2.40)  GLN275(1.92)  HIS372(2.20)  HIS372(2.31)  GLN275(2.30)  HIS200(3.03)  HIS200(4.21)  HIS200 (5.06)  HIS200 (5.00)  HIS372 (5.09) |
| 30 | **Quercetin-3-O-glucuronide** | -8.5 | HIS193(2.83)  HIS193(2.48)  THR198(2.36)  THR198(1.99)  THR198(2.01)  PHE196(3.34)  VAL277(3.54)  VAL277(5.29)  LEU280(5.38)  VAL277(4.65) |
| 31 | **Sinapinic acid** | -5.8 | HIS200(2.22)  PHE196(3.59)  VAL277(4.43)  HIS193 (4.36)  HIS200 (5.31)  LYS197(5.28) |
| 32 | **Resveratrol** | -6.3 | TYR341(2.78)  ASP333(4.56)  HIS337(3.69) |
| 33 | **Apigetrin** | -8.5 | GLN178(2.71)  HIS337(2.28)  GLN178(2.13)  HIS337(3.19)  PRO500(3.15) |
| 34 | **Phloretin** | -6.6 | HIS193(2.26)  THR198(2.38)  GLU276(2.20)  ASN368(2.21)  HIS372(4.50)  HIS193(2.43)  VAL277(4.55) |
